# Associations of Sarcopenia and Sarcopenic Obesity Using Different Obesity Definitions With Memory Decline

**DOI:** 10.1111/1753-0407.70210

**Published:** 2026-03-12

**Authors:** Ying Yue Huang, Wei Sen Zhang, Jiao Wang, Ya Li Jin, Kar Keung Cheng, Tai Hing Lam, Lin Xu

**Affiliations:** ^1^ School of Public Health, Sun Yat‐Sen University Guangzhou China; ^2^ Greater Bay Area Public Health Research Collaboration Guangdong China; ^3^ Guangzhou Center for Disease Control and Prevention Guangzhou China; ^4^ Guangzhou Twelfth People's Hospital Guangzhou China; ^5^ Department of Applied Health Sciences School of Health Sciences, College of Medicine and Health, University of Birmingham Birmingham UK; ^6^ School of Public Health, the University of Hong Kong Hong Kong China

**Keywords:** memory, mendelian randomization, sarcopenia, sarcopenic obesity, waist‐to‐hip‐to‐height ratio

## Abstract

**Aims:**

To explore the associations of sarcopenia and sarcopenic obesity (SO) with memory.

**Materials and Methods:**

Data from the Guangzhou Biobank Cohort Study (GBCS) were used to conduct both cross‐sectional (*n* = 6390) and longitudinal (*n* = 3979) analyses. Sarcopenia was defined according to the Asian Working Group for Sarcopenia 2019, while obesity was defined by 20 obesity indicators. Memory was assessed by the Delayed Word Recall Test (DWRT). Linear regression was used to analyze these associations. Two‐sample Mendelian randomization (2SMR) was used to investigate potential causal associations of sarcopenia‐related traits with memory.

**Results:**

Compared to participants without sarcopenia, those with sarcopenia had lower baseline and follow‐up DWRT score, and greater decrease in mean annual change (MAC) and MAC rate of DWRT score, with adjusted βs (95% CIs) being −0.24 (−0.33 to −0.14), −0.10 (−0.20 to −0.004), −0.03 (−0.06 to −0.001) and −0.79 (−1.57 to −0.01), respectively. Among all the SO definitions, the associations of SO defined by waist‐to‐hip‐to‐height ratio (WHHR) appeared to be the strongest (−0.36 [−0.50 to −0.22], −0.31 [−0.45 to −0.17], −0.09 [−0.14 to −0.05] and −2.70 [−3.81 to −1.59]), and stronger than those with sarcopenia only or obesity only. In 2SMR, walking pace (one of the sarcopenia indicators) was positively associated with working memory (1.61 [0.59, 2.62]).

**Conclusions:**

Sarcopenia and SO were associated with faster memory decline. Using the central obesity indicator WHHR to define SO may better predict late‐life memory.

## Highlights

Middle‐aged and older people with sarcopenia and sarcopenic obesity (SO) showed poorer memory function and faster memory decline, with no clear sarcopenia‐obesity interaction, and SO defined by the central obesity indicator WHHR could be the best for SO definition to predict late‐life memory. Our findings emphasize the need to manage sarcopenia, regardless of obesity status, as well as control central obesity, to potentially mitigate memory decline in later life. Further research on preventive strategies and interventions is needed.

## Graphical abstract

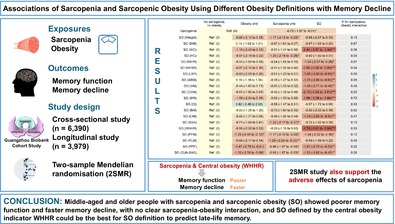

## 1 Introduction

Sarcopenia, a syndrome defined by the loss of skeletal muscle mass and strength, is common in older populations, which may adversely affect brain health, as contracting skeletal muscle is an important source of neurotrophic factors [1]. However, reports on the association between sarcopenia and memory [1], one of the most common symptoms of MCI and dementia, are limited. Although emerging evidence suggests that sarcopenia may be associated with MCI and a higher risk of dementia [2, 3], most studies reported on global cognitive function, which comprises multiple domains such that the specific effects of sarcopenia on memory are unclear.

Sarcopenia can coexist with obesity, especially in older people. The co‐occurrence, termed sarcopenic obesity (SO), may exacerbate both physical and cognitive decline, further complicating the associations. However, how to define SO remains controversial, due to no definition of obesity accepted for all populations [4]. Moreover, most previous studies on SO‐cognition association defined obesity using BMI [5, 6], which likely contributes to “obesity paradox” [7] and underestimates of the detrimental effect of obesity and SO. Thus, it is crucial to study the associations of SO with memory using different definitions of SO.

Addressing these gaps could enhance our understanding of sarcopenia and SO, and further provide evidence for better memory decline prevention strategies that integrate the management of muscle and fat mass, which has not yet been recognized by current dementia report [8]. Results could support development of optimal definitions of SO for advancing screening accuracy and clinical judgment. Moreover, most studies to date have been cross‐sectional, and the temporal progression of these conditions is unclear. Longitudinal studies, particularly those based on the general populations rather than patient cohorts, are less subjective to selection bias. Furthermore, due to the nature of observational studies, both cross‐sectional and longitudinal studies cannot make causal inferences. Mendelian randomization (MR) has emerged as an effective approach for estimating causal associations that are less likely to be confounded [9].

In this study, we used data from the Guangzhou Biobank Cohort Study (GBCS) to conduct both cross‐sectional and longitudinal analyses, examining the associations of sarcopenia and SO from various definitions with memory function and memory decline in middle‐aged and older Chinese adults. Moreover, we conducted a two‐sample MR study to investigate potential causal associations of sarcopenia‐related traits and memory decline.

## 2 Materials and Methods

### 2.1 Conventional Observational Study

#### 2.1.1 Study Participants

The GBCS is a three‐way collaboration involving the Guangzhou Twelfth People's Hospital and the University of Hong Kong, China, and the University of Birmingham, UK. Details of the methods have been reported previously [10, 11]. The study was approved by the Guangzhou Medical Ethics Committee of the Chinese Medical Association (No. 2020044), and all participants provided written informed consent prior to participation.

### 2.2 Exposures

Sarcopenia and SO at baseline were exposure variables. Sarcopenia was defined as low muscle strength or low physical performance based on the 2019 criteria recommended by the Asian Working Group for Sarcopenia (AWGS). Muscle strength was assessed by grip strength. Low muscle strength was classified as less than 28 kg for men and less than 18 kg for women. Physical performance was assessed using gait speed, measured by the timed up‐and‐go test (TUGT). Gait speed was defined as low if it was less than 1.0 m/s, as per AWGS criteria. Details of the grip strength and TUGT measurements have been reported in our previous papers [12, 13].

Obesity was assessed using 20 obesity indicators including weight, BMI, WC, hip circumference (HC), waist‐to‐hip ratio (WHR), waist‐to‐height ratio (WHtR), lipid accumulation product (LAP), a body shape index (ABSI), visceral adiposity index (VAI), Chinese VAI (CVAI), body roundness index (BRI), conicity index, body adiposity index (BAI), cardiometabolic index (CMI), body surface area (BSA), waist‐to‐hip‐to‐height ratio (WHHR), predicted fat mass (PFM), predicted lean mass (PLM), predicted percent fat (PPF) and Clínica Universidad de Navarra‐Body Adiposity Estimator (CUN‐BAE). Indicators reflecting general adiposity or overall body size included weight, BMI, HC, BAI, BSA, PFM, PLM, PPF, and CUN‐BAE. Indicators reflecting central adiposity and/or visceral adipose dysfunction included WC, WHR, WHtR, LAP, ABSI, VAI, CVAI, BRI, conicity index, CMI, and WHHR. In particular, LAP, VAI/CVAI and CMI incorporate lipid parameters and have been used to approximate visceral adiposity‐related metabolic risk, whereas WHHR integrates waist, hip and height information to characterize central adiposity/body shape in a manner that may be clinically relevant for risk stratification. Detailed description of the searching process and measurements of obesity indicators are shown in Supplementary Methods. Obesity was defined by the following cutoffs: high BMI (≥ 27.5 kg/m^2^) [14]; high WC (≥ 90 cm in men and ≥ 80 cm in women) [15]; high WHR (men ≥ 0.9 and women ≥ 0.8) [16]; high WHtR (≥ 0.5) [17]; or the highest sex‐specific tertile for the other obesity indicators.

SO was defined as the coexistence of sarcopenia and obesity. Based on the presence or absence of these conditions, participants were categorized into four subgroups: no sarcopenia or obesity, sarcopenia only, obesity only, and SO.

### 2.3 Outcomes

Memory function was assessed using the delayed 10‐word recall test (DWRT) at both baseline (2003–2008) and follow‐up (2008–2012), as reported in previous GBCS papers [18, 19]. Then, according to previous GBCS papers [18, 19], memory decline was calculated by mean annual change and mean annual rate of change in DWRT score. Mean annual change = (follow‐up score—baseline score)/follow‐up time, and mean annual rate of change = (mean annual change/baseline score) * 100. For categorical outcomes, memory impairment was defined by DWRT score < 4, corresponding to one standard deviation (SD) below the mean (mean ± SD: 5.9 ± 2.0), while memory decline was defined by change in DWRT score < 0.

### 2.4 Mendelian Randomization

#### 2.4.1 Genetic Associations With Exposures

Due to the lack of a published genome‐wide association study (GWAS) specifically for sarcopenia, based on current diagnostic criteria [20], we selected proxy measures for muscle strength, physical performance and muscle mass for the MR analysis. These included grip strength as a measure of muscle strength, walking pace as a measure of physical performance, and appendicular lean mass (ALM) and whole‐body lean mass (WBLM) as measures of muscle mass. Genetic associations with these traits were obtained from the largest and most recent publicly available GWAS data from the UK Biobank. Hand grip strength (left or right) was assessed using a Jamar J00105 hydraulic hand dynamometer. Walking pace was self‐reported, with participants selecting from “slow,” “steady/average,” or “brisk”, which were coded as 0, 1 and 2, respectively. ALM and WBLM were measured using the Tanita BC 418ma body fat analyzer. Additionally, GWAS data for muscle weakness (grip strength < 30 kg for men; < 20 kg for women) were obtained from a meta‐analysis of 22 independent cohorts. Detailed information on the GWAS datasets used for sarcopenia‐related traits is presented in Table S1.

#### 2.4.2 Genetic Associations With Outcome

Working memory was assessed using the N‐back task (two‐back design) to obtain the discriminability index (d′), with a higher d′ indicating better working memory performance. Genetic associations for working memory were derived from the GWAS from the Age 24 Clinic of the Avon Longitudinal Study of Parents and Children (ALSPAC). Data on memory loss were obtained from the FinnGen database. Table S1 shows the detailed information of these GWAS data for these outcomes.

### 2.5 Statistical Analysis

#### 2.5.1 Conventional Observational Study

Chi‐square test and analysis of variance were used to compare baseline characteristics of categorical and continuous variables according to the presence or absence of sarcopenia. In cross‐sectional analyses, multivariable linear regression was used to analyze the associations of sarcopenia and SO with DWRT score at baseline. In longitudinal analyses, multivariable linear regression and generalized estimating equation (GEE) were used to analyze the associations of sarcopenia and SO with follow‐up DWRT score, mean annual change, and mean annual rate of change in DWRT score. Results were presented as regression coefficients (βs) with 95% confidence intervals (CIs). Additionally, interactions between sarcopenia and obesity, and interactions of SO with sex, age, and baseline memory status were analyzed by fitting models with and without the interaction term, with statistical significance determined by the likelihood ratio test of the difference between the two models. In addition, we conducted receiver operating characteristic (ROC) analyses for the obesity indicator that demonstrated the strongest association with memory outcomes. The optimal cutoff value for the selected indicator was identified using the Youden index to provide preliminary insights into its potential clinical applicability.

#### 2.5.2 Mendelian Randomization

The causal associations of sarcopenia‐related traits with memory were analyzed using two‐sample MR. First, we obtained single nucleotide polymorphisms (SNPs) strongly associated with exposures with a *p*‐value < 5 × 10^−8^. Second, linkage disequilibrium (LD) between SNPs was identified using “ld_clump” R package, and those highly correlated SNPs (*r* ^2^ ≥ 0.001) with higher *p* values were excluded. Third, the effect alleles for the outcomes were aligned to be consistent with those of the exposures. Moreover, F‐statistics of the instruments were calculated by the square of SNP‐exposure association divided by its variance, with the mean F statistics used to evaluate instrument strength. In the primary analyses, we used the inverse‐variance weighted (IVW) method, given its efficiency in generating causal effect estimates under the assumption of valid instrumental variables. Moreover, as different MR methods incorporate varying assumptions regarding pleiotropy and instrument validity, we further conducted sensitivity analyses using the weighted median estimator (WM), MR‐Egger regression, and the MR pleiotropy residual sum and outlier test (MR‐PRESSO) to assess the robustness of the findings. A zero intercept from MR‐Egger (*p* > 0.05) indicated no potential horizontal pleiotropy.

All statistical analyses were done using Stata version 16.0 (StataCorp LP, College Station, TX) and R version 4.4.1 (R Foundation for Statistical Computing, Vienna, Austria). The “TwoSampleMR”, “MendelianRandomization”, “MRPRESSO” and “ComplexHeatmap” packages were used. All tests were two‐sided, with *p* < 0.05 as statistically significant.

## 3 Results

### 3.1 Characteristics of Participants

Of 10 088 participants recruited from 2006 to 2008, after excluding those with duplicate information (*N* = 39) and missing information on sarcopenia (*N* = 3094), obesity indicators (*N* = 119), DWRT score (*N* = 231), and potential confounders (*N* = 922), 6390 participants with all variables of interest were included in cross‐sectional analyses. Among these, 4085 participants returned for the first follow‐up examination (2008–2012), with an average follow‐up duration of 3.56 (SD = 0.69) years. After excluding those with missing information on DWRT score in 2008–2012 (*N* = 106), 3979 participants were included in the longitudinal analyses.

Table S2 shows that in cross‐sectional analyses, among the 20 obesity indicators, 16 were significantly higher in participants with sarcopenia, including BMI, WC, WHR, WHtR, LAP, ABSI, VAI, CVAI, BRI, conicity index, BAI, CMI, WHHR, PFM, PPF and CUN‐BAE (*p* from < 0.001 to 0.01). In contrast, weight, BSA, and PLM were lower in participants with sarcopenia (*p* from < 0.001 to 0.007). Moreover, participants with sarcopenia had lower DWRT score. Similar patterns were observed among participants in longitudinal analyses. Table S3 shows that the lost‐to‐follow‐up populations were older, had higher proportion of those with lower education and lower personal income, and current smokers (*p* from < 0.001 to 0.004), but lower proportion of those with good health status (*p* = 0.001). Moreover, among the 20 obesity indicators, 12 were significantly higher in the lost‐to‐follow‐up populations, including WC, WHR, WHtR, LAP, ABSI, VAI, CVAI, BRI, conicity index, BAI, CMI and WHHR (*p* values from < 0.001 to 0.04).

### 3.2 Sarcopenia and Memory

#### 3.2.1 Conventional Observational Study

**FIGURE 1:**
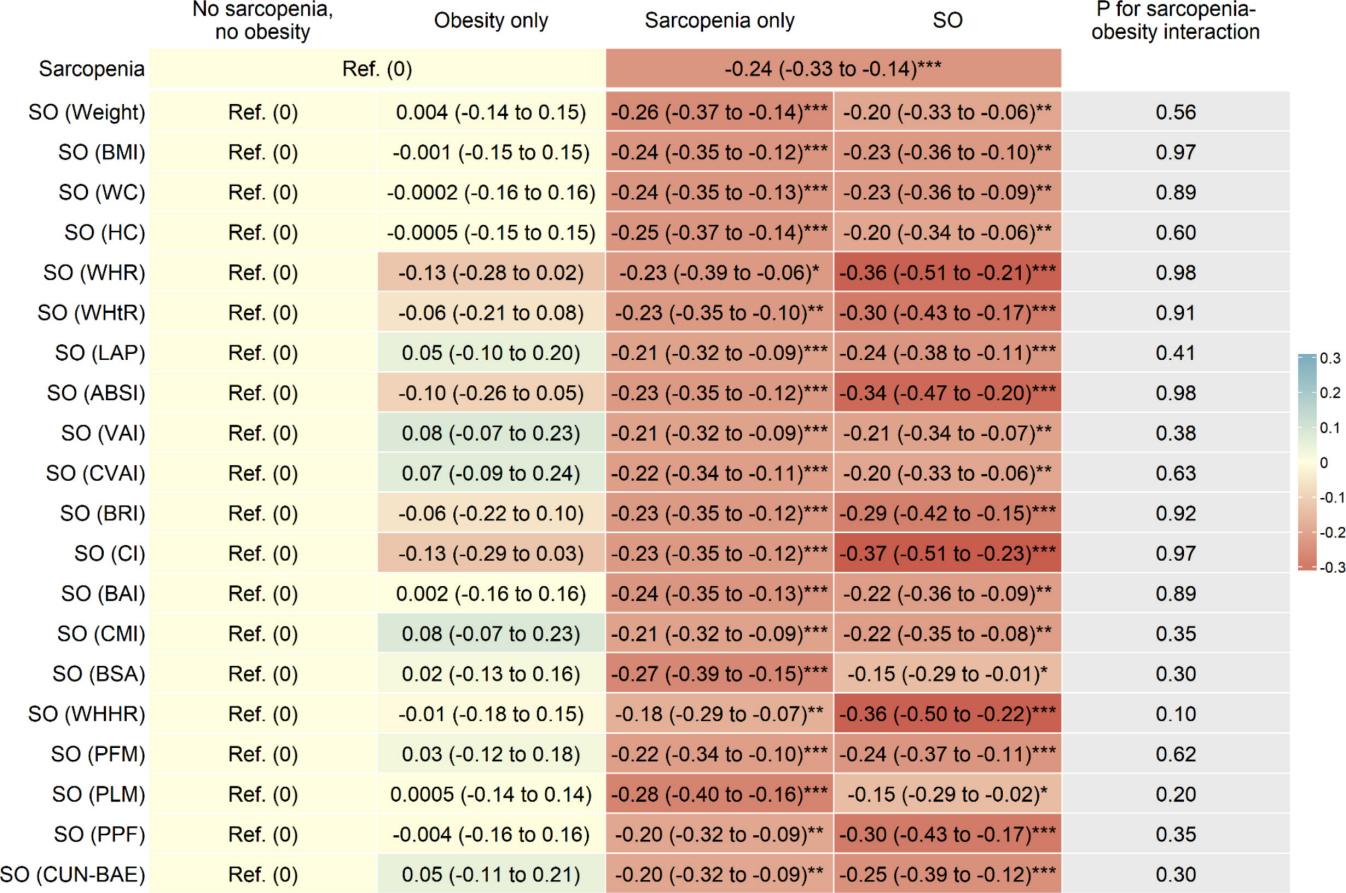
Associations of baseline sarcopenia status and sarcopenic obesity with memory function at baseline, and sarcopenia‐obesity interaction. ABSI, A body shape index; BAI, Body adiposity index; BMI, Body mass index; BRI, Body roundness index; BSA, Body surface area; CI, conicity index; CMI, Cardiometabolic index; CUN‐BAE, Clínica Universidad de Navarra‐Body Adiposity Estimator; CVAI, Chinese visceral adiposity index; DWRT, Delayed Word Recall Test; HC, hip circumference; LAP, Lipid accumulation product; PFM, Predicted fat mass; PLM, Predicted lean mass; PPF, Predicated percent fat; Ref, reference; SO, sarcopenic obesity; VAI, Visceral adiposity index; WC, Waist circumference; WHHR, Waist‐to‐hip‐to‐height ratio; WHR, Waist‐to‐hip ratio; WHtR, Waist‐to‐height ratio. Adjusted β (95% CI): Adjusted for sex, age, education, occupation, personal income, physical activity, drinking, smoking, and self‐rated health. *p < 0.05, **p < 0.01, ***p < 0.001.

**FIGURE 2:**
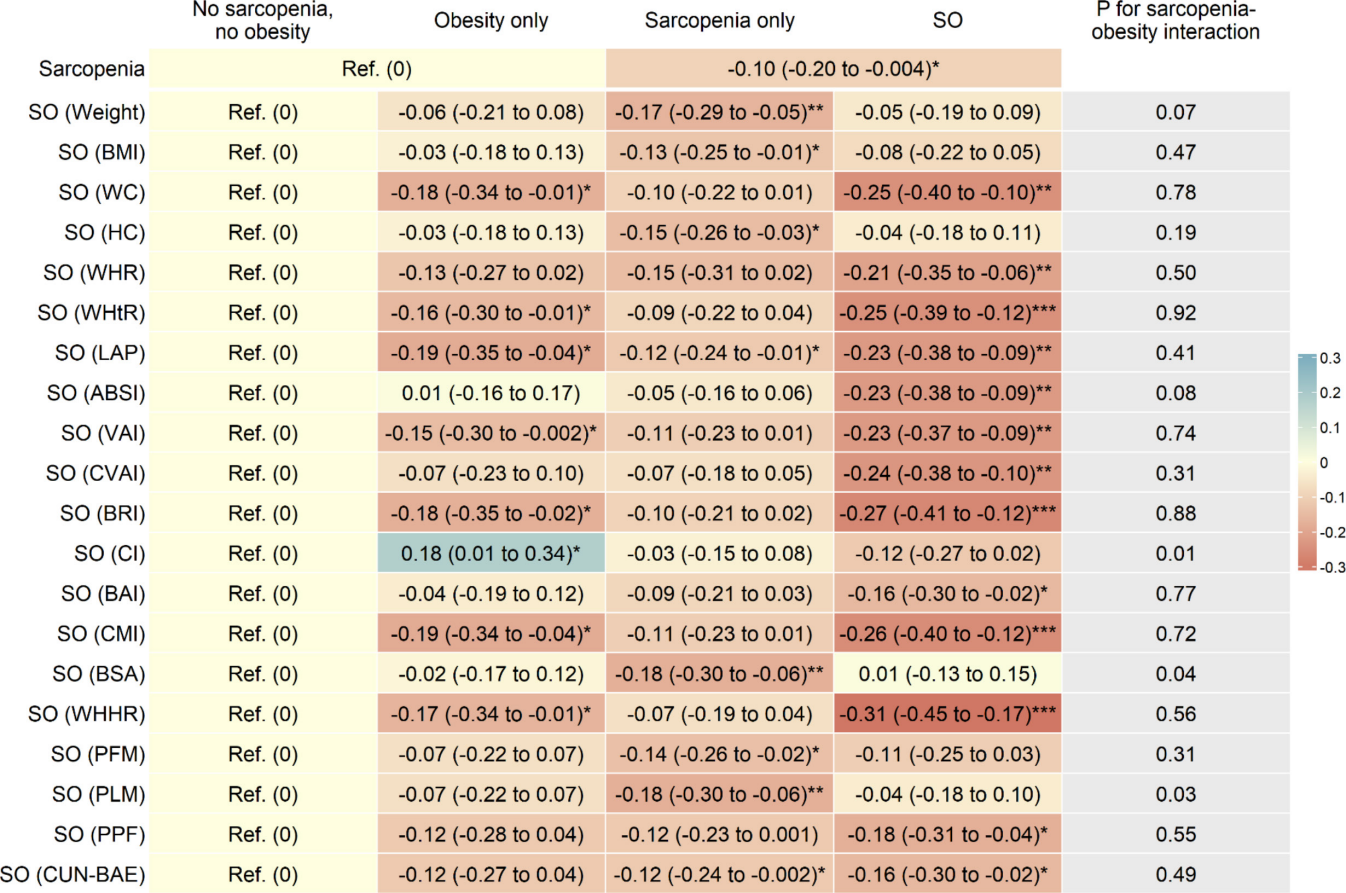
Associations of baseline sarcopenia status and sarcopenic obesity with memory function at follow‐up, and sarcopenia‐obesity interaction. ABSI, A body shape index; BAI, Body adiposity index; BMI, Body mass index; BRI, Body roundness index; BSA, Body surface area; CI, conicity index; CMI, Cardiometabolic index; CUN‐BAE, Clínica Universidad de Navarra‐Body Adiposity Estimator; CVAI, Chinese visceral adiposity index; DWRT, Delayed Word Recall Test; HC, hip circumference; LAP, Lipid accumulation product; PFM, Predicted fat mass; PLM, Predicted lean mass; PPF, Predicated percent fat; Ref, reference; SO, sarcopenic obesity; VAI, Visceral adiposity index; WC, Waist circumference; WHHR, Waist‐to‐hip‐to‐height ratio; WHR, Waist‐to‐hip ratio; WHtR, Waist‐to‐height ratio. Adjusted β (95% CI): Adjusted for sex, age, education, occupation, personal income, physical activity, drinking, smoking, self‐rated health and baseline DWRT score *p < 0.05, **p < 0.01, ***p < 0.001.

**FIGURE 3:**
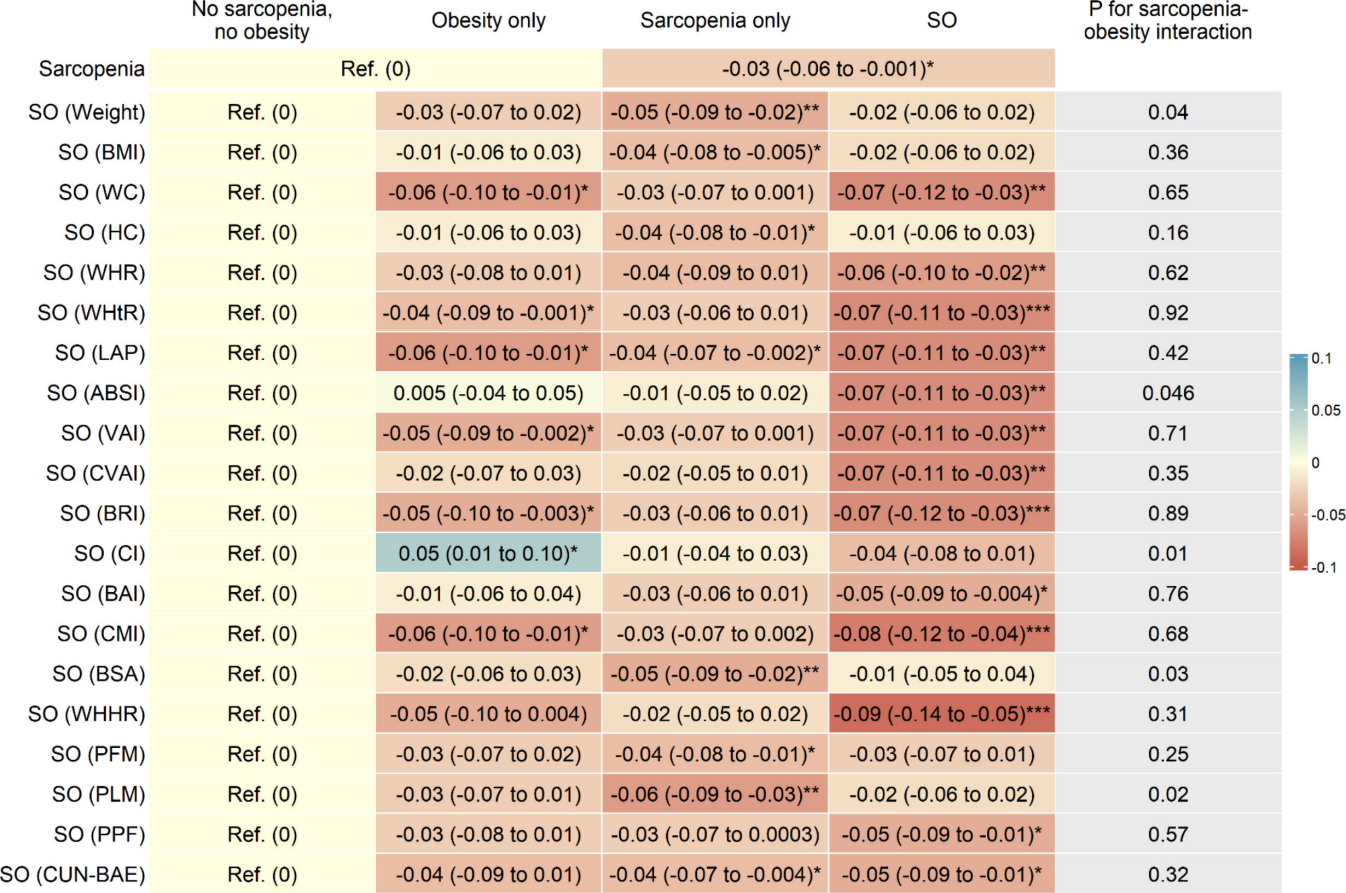
Associations of baseline sarcopenia status and sarcopenic obesity with mean annual change of memory function, and sarcopenia‐obesity interaction. ABSI, A body shape index; BAI, Body adiposity index; BMI, Body mass index; BRI, Body roundness index; BSA, Body surface area; CI, conicity index; CMI, Cardiometabolic index; CUN‐BAE, Clínica Universidad de Navarra‐Body Adiposity Estimator; CVAI, Chinese visceral adiposity index; DWRT, Delayed Word Recall Test; HC, hip circumference; LAP, Lipid accumulation product; PFM, Predicted fat mass; PLM, Predicted lean mass; PPF, Predicated percent fat; Ref, reference; SO, sarcopenic obesity; VAI, Visceral adiposity index; WC, Waist circumference; WHHR, Waist‐to‐hip‐to‐height ratio; WHR, Waist‐to‐hip ratio; WHtR, Waist‐to‐height ratio. Adjusted β (95% CI): Adjusted for sex, age, education, occupation, personal income, physical activity, drinking, smoking, self‐rated health and baseline DWRT score. *p < 0.05, **p < 0.01, ***p < 0.001.

**FIGURE 4:**
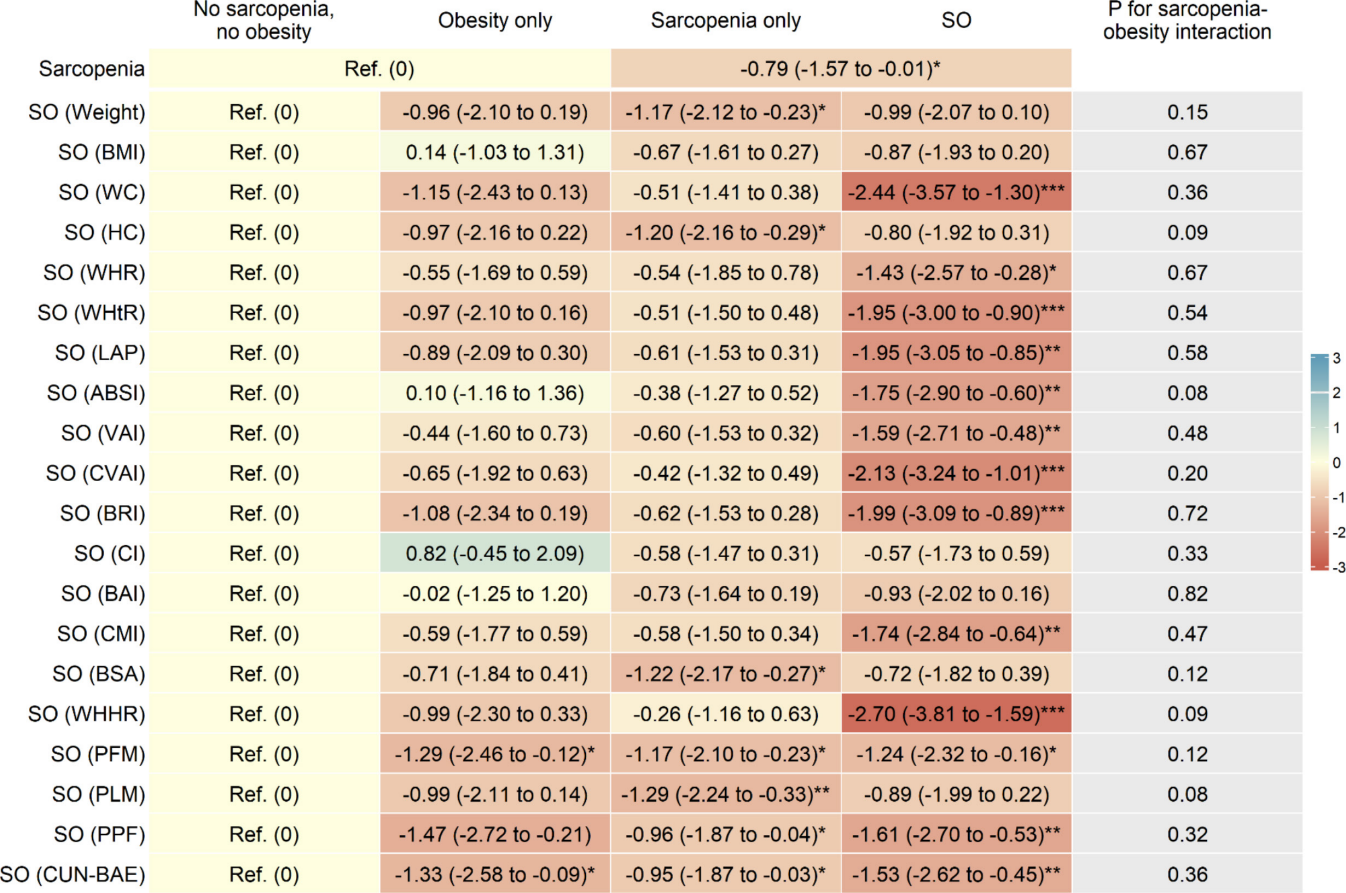
Associations of baseline sarcopenia status and sarcopenic obesity with mean annual change rate of memory function, and sarcopenia‐obesity interaction. ABSI, A body shape index; BAI, Body adiposity index; BMI, Body mass index; BRI, Body roundness index; BSA, Body surface area; CI, conicity index; CMI, Cardiometabolic index; CUN‐BAE, Clínica Universidad de Navarra‐Body Adiposity Estimator; CVAI, Chinese visceral adiposity index; DWRT, Delayed Word Recall Test; HC, hip circumference; LAP, Lipid accumulation product; PFM, Predicted fat mass; PLM, Predicted lean mass; PPF, Predicated percent fat; Ref, reference; SO, sarcopenic obesity; VAI, Visceral adiposity index; WC, Waist circumference; WHHR, Waist‐to‐hip‐to‐height ratio; WHR, Waist‐to‐hip ratio; WHtR, Waist‐to‐height ratio. Adjusted β (95% CI): Adjusted for sex, age, education, occupation, personal income, physical activity, drinking, smoking, self‐rated health and baseline DWRT score.*p < 0.05, **p < 0.01, ***p < 0.001. Our findings emphasize the need to manage sarcopenia, regardless of obesity status, as well as control central obesity, to potentially mitigate memory decline in later life. Further research on preventive strategies and interventions is needed.

Figure 1, Table S4 and Figure S1 show that after adjusting for sex, age, education, occupation, personal income, physical activity, drinking, smoking and self‐rated health, compared to those without sarcopenia, participants with sarcopenia had lower baseline DWRT score, with adjusted β (95% CI) being −0.24 (−0.33 to −0.14). After additionally adjusting for baseline DWRT score, sarcopenia was associated with lower follow‐up DWRT score (β (95% CI): −0.10 (−0.20 to −0.004)) (Figure 2, Table S5 and Figure S2). The results were consistent when analyzed using GEE model (β (95% CI): −0.13 (−0.19 to −0.07)) (Table S6). Moreover, sarcopenia was associated with greater decrease in the mean annual change (Figure 3, Table S7 and Figure S3) and the annual rate of change in DWRT score (βs (95% CIs): −0.03 (−0.06 to −0.001) and −0.79 (−1.57 to −0.01), respectively) (Figure 4, Table S8 and Figure S4).

#### 3.2.2 Mendelian Randomization

The number of SNPs associated with hand grip strength (left), hand grip strength (right), muscle mass, walking pace, ALM and WBLM at genome‐wide significance (*p*‐value < 5 × 10^−8^) and without high LD (*r* ^2^ ≥ 0.001) was 157, 176, 17, 65, 690 and 556, respectively. Of these, 146, 166, 13, 60, 588 and 528 SNPs were found in working memory dataset, and subsequently 3, 8, 1, 1, 17 and 17 SNPs were excluded due to being palindromic, respectively. Similarly, 148, 169, 16, 60, 622 and 544 SNPs were found in memory loss dataset, with 3, 8, 1, 2, 27, and 24 SNPs excluded due to palindromic status. Figures S5 and S6 show the selection process of genetic instruments for sarcopenia‐related traits.

Table 1 shows no association of hand grip strength (left), hand grip strength (right), muscle mass, ALM and WBLM using any methods. Genetically determined walking pace was positively associated with working memory using IVW (β (95% CI): 1.61 (0.59 to 2.62)), though no association was found with memory loss. The MR‐Egger intercept suggested no evidence of directional horizontal pleiotropy (all *p* > 0.05) except for walking pace on memory. However, similar trends of walking pace on working memory were found using WM, MR Egger and MR‐PRESSO.

**TABLE 1:** Associations of genetically determined sarcopenia‐related traits with memory.

Exposures	Outcomes	Mendelian randomization method	SNPs used	Mean F‐statistic	β/OR	95% confidence interval	*p*	Cochran's Q (*I* ^2^)	MR‐Egger intercept (*p*)	Outliers from MR PRESSO
Hand grip strength (left)	Working memory	IVW	143	47	−0.06	−0.56 to 0.44	0.81	171.2 (17.1%)	−0.02 (0.11)	rs13107325
		WM			−0.46	−1.15 to 0.23	0.19			
		MR Egger			1.47	−0.49 to 3.42	0.14			
		MR‐PRESSO			−0.17	−0.65 to 0.31	0.48			
	Memory loss	IVW	145	48	1.35	0.84 to 2.20	0.22	188.4 (23.6%)	0.003 (0.78)	NA
		WM			1.79	0.93 to 3.44	0.08			
		MR Egger			1.05	0.17 to 6.59	0.96			
		MR‐PRESSO			1.36	0.84 to 2.20	0.22			
Hand grip strength (right)	Working memory	IVW	158	46	−0.02	−0.45 to 0.42	0.95	154.9 (0.0%)	−0.02 (0.06)	NA
		WM			−0.05	−0.72 to 0.61	0.88			
		MR Egger			1.51	−0.14 to 3.17	0.07			
		MR‐PRESSO			−0.01	−0.45 to 0.42	0.95			
	Memory loss	IVW	161	47	1.17	0.76 to 1.81	0.47	186.0 (14.0%)	−0.007 (0.47)	NA
		WM			1.10	0.60 to 2.03	0.76			
		MR Egger			2.06	0.42 to 10.10	0.37			
		MR‐PRESSO			1.17	0.76 to 1.81	0.47			
Muscle weakness	Working memory	IVW	12	40	−0.12	−0.63 to 0.40	0.66	24.6 (55.3%)	0.07 (0.19)	rs13107325
		WM			0.27	−0.20 to 0.74	0.26			
		MR Egger			−1.31	−3.17 to 0.55	0.17			
		MR‐PRESSO			0.11	−0.20 to 0.41	0.51			
	Memory loss	IVW	15	40	0.79	0.58 to 1.07	0.12	15.2 (7.9%)	0.02 (0.44)	NA
		WM			0.77	0.51 to 1.15	0.20			
		MR Egger			0.53	0.18 to 1.54	0.24			
		MR‐PRESSO			0.79	0.58 to 1.07	0.14			
Walking pace	Working memory	IVW	59	41	**1.61**	**0.59 to 2.62**	**0.002**	75.8 (23.5%)	−0.06 (0.001)	NA
		WM			**1.88**	**0.54 to 3.23**	**0.006**			
		MR Egger			**8.05**	**4.14 to 11.95**	**< 0.001**			
		MR‐PRESSO			**1.61**	**0.59 to 2.62**	**0.003**			
	Memory loss	IVW	58	41	0.87	0.36 to 2.06	0.74	54.7 (0.0%)	0.05 (0.03)	NA
		WM			0.86	0.25 to 3.00	0.81			
		MR Egger			0.01	0.0001 to 0.50	0.02			
		MR‐PRESSO			0.87	0.37 to 2.03	0.74			
Appendicular lean mass	Working memory	IVW	571	100	0.05	−0.07 to 0.17	0.43	556.7 (0.0%)	−0.001 (0.76)	NA
		WM			0.07	−0.13 to 0.27	0.51			
		MR Egger			0.09	−0.20 to 0.38	0.54			
		MR‐PRESSO			0.05	−0.07 to 0.17	0.43			
	Memory loss	IVW	595	99	1.05	0.94 to 1.17	0.42	582.1 (0.0%)	0.001 (0.90)	NA
		WM			1.07	0.89 to 1.29	0.46			
		MR Egger			1.03	0.80 to 1.34	0.81			
		MR‐PRESSO			1.05	0.94 to 1.17	0.42			
Whole‐body lean mass	Working memory	IVW	511	84	−0.10	−0.31 to 0.12	0.37	472.8 (0.0%)	0.002 (0.65)	NA
		WM			−0.24	−0.58 to 0.09	0.16			
		MR Egger			−0.21	−0.74 to 0.31	0.43			
		MR‐PRESSO			−0.10	−0.31 to 0.11	0.35			
	Memory loss	IVW	520	85	0.89	0.73 to 1.10	0.28	539.1 (3.7%)	−0.001 (0.98)	NA
		WM			0.94	0.69 to 1.29	0.72			
		MR Egger			0.90	0.56 to 1.45	0.67			
		MR‐PRESSO			0.89	0.73 to 1.10	0.28			

*Note:* Bold: *p* < 0.05.

Abbreviations: IVW, inverse‐variance weighted; MR‐PRESSO, Mendelian randomization pleiotropy residual sum and outlier; SNP, single nucleotide polymorphism; WM, weighted median method.

### 3.3 Sarcopenic Obesity and Memory

#### 3.3.1 Baseline DWRT

Figure 1, Table S4 and Figure S1 show that compared to those without sarcopenia and obesity, obesity only and sarcopenia only were generally associated with lower baseline DWRT scores, although some associations were nonsignificant; all 20 SO phenotypes were significantly associated with lower baseline DWRT scores. Among them, the strongest three associations were observed for SO defined by conicity index, WHHR and WHR, with βs (95% CIs) being −0.37 (−0.51 to −0.23), −0.36 (−0.50 to −0.22) and −0.36 (−0.51 to −0.21), respectively, although the 95% CIs overlapped. These effect sizes appeared stronger than those with obesity only or sarcopenia only. There was no interaction between sarcopenia and obesity on baseline DWRT score (*p* for interaction from 0.10 to 0.98).

#### 3.3.2 Follow‐Up DWRT

Figure 2, Table S5 and Figure S2 show that 13 SO phenotypes defined by WHHR, BRI, CMI, WC, WHtR, CVAI, LAP, ABSI, VAI, WHR, PPF, BAI and CUN‐BAE were significantly associated with lower follow‐up DWRT scores. Among them, the associations for SO defined by WHHR, BRI and CMI appeared to be strongest, with βs (95% CIs) being −0.31 (−0.45 to −0.17), −0.27 (−0.41 to −0.12) and −0.26 (−0.40 to −0.12), respectively. These associations appeared stronger than those for obesity only or sarcopenia only. The results were similar when analyzed using GEE models (Table S6). Moreover, Figure 2 and Table S5 show significant interactions between sarcopenia and three obesity indicators (conicity index, PLM and BSA) on follow‐up DWRT score (*p* for interaction from 0.01 to 0.04). While sarcopenia only was significantly associated with lower follow‐up DWRT score, no significant association for SO defined by conicity index, PLM and BSA was found, with βs (95% CIs) being −0.12 (−0.27 to 0.02), −0.04 (−0.18 to 0.10) and 0.01 (−0.13 to 0.15), respectively.

#### 3.3.3 Annual Change of DWRT

Figure 3, Table S7 and Figure S3 show that 13 SO phenotypes defined by WHHR, CMI, WHtR, BRI, WC, LAP, ABSI, VAI, CVAI, WHR, BAI, PPF and CUN‐BAE were significantly associated with greater decreases in the mean annual change of DWRT score. Among them, three associations appeared to be strongest for SO defined by WHHR, CMI and WHtR, with βs (95% CIs) being −0.09 (−0.14 to −0.05), −0.08 (−0.12 to −0.04) and −0.07 (−0.11 to −0.03), respectively. These effect sizes appeared stronger than those with obesity only or sarcopenia only. Significant interactions were also observed between sarcopenia and five obesity indicators (conicity index, PLM, BSA, weight and ABSI) on the mean annual change of DWRT score (*p* for interaction from 0.01 to 0.046). While sarcopenia only was significantly associated with greater decreases in mean annual change of DWRT score, no significant associations were observed for SO defined by conicity index, PLM, BSA and weight with mean annual change of DWRT score, with βs (95% CIs) being −0.04 (−0.08 to 0.01), −0.02 (−0.06 to 0.02), −0.01 (−0.05 to 0.04) and −0.02 (−0.06 to 0.02), respectively. Compared to those without sarcopenia and obesity, as defined by ABSI, those with obesity only or sarcopenia only had similar mean annual change of DWRT score (βs (95% CIs): 0.005 (−0.04 to 0.05) and −0.01 (−0.05 to 0.02)), while those with SO had a greater decrease (with 95% CI not overlapping) in mean annual change of DWRT score (β (95% CI): −0.07 (−0.11 to −0.03)).

#### 3.3.4 Annual Change Rate of DWRT

Figure 4, Table S8 and Figure S4 show that 13 SO phenotypes, including those defined by WHHR, WC, CVAI, BRI, WHtR, LAP, ABSI, CMI, PPF, VAI, CUN‐BAE, WHR, and PFM, were significantly associated with greater declines in mean annual change rate of DWRT score. Among them, three associations appeared to be strongest for SO defined by WHHR, WC and CVAI, with βs (95% CIs) being −2.70 (−3.81 to −1.59), −2.44 (−3.57 to −1.30) and −2.13 (−3.24 to −1.01), respectively. These associations appeared stronger than those for obesity only or sarcopenia only. There was no interaction between sarcopenia and obesity on the mean annual rate of change in DWRT score (*p* for interaction from 0.08 to 0.82).

#### 3.3.5 Optimal Cutoff Value for WHHR

As WHHR showed the strongest and most consistent association with memory outcomes, we further conducted ROC analysis to evaluate its potential clinical threshold. Based on the maximum Youden index, the optimal WHHR cutoff for predicting baseline memory was 0.0555, while the optimal WHHR cutoff for predicting follow‐up memory and annual change in memory was 0.0565.

#### 3.3.6 Subgroup Analyses by Sex, Age, and Baseline Memory Status

Tables S9–S20 show that in subgroup analyses by sex, age and baseline memory status, although some interaction terms reached statistical significance, the 95% CIs of estimates for SO across different subgroups generally overlapped.

## 4 Discussion

We have first shown significant associations of sarcopenia and SO with poorer memory function and faster memory decline using both observational (cross‐sectional and longitudinal) and MR analyses, with the strongest and most consistent associations observed for SO defined by WHHR.

Our findings confirm and extend the current literature by demonstrating that sarcopenia was associated with both poorer memory function and faster memory decline. Previous studies showed that sarcopenia was associated with poorer memory function [21, 22, 23, 24, 25]. However, most of these studies were cross‐sectional and limited by relatively small sample sizes (*N* ≤ 1181) [21, 22, 23, 24]. Only the English Longitudinal Study of Aging showed the association of sarcopenia with memory function over 10 years of follow‐up (*N* = 2738) [25]. Our study strengthens the existing evidence by confirming the adverse effect of sarcopenia on memory function in a larger sample, both in cross‐sectional (*N* = 6390) and longitudinal (*N* = 3979) analyses. Moreover, we found that sarcopenia predicted and hence might also exacerbate memory decline, highlighting the importance of early interventions aimed at improving muscle mass and physical function to potentially mitigate memory impairment and delay memory decline in late life. The detrimental effect of sarcopenia on memory performance may be explained by the muscle‐brain axis. Myokines, cytokines, and chemokines produced by skeletal muscle cells may contribute to systemic homeostasis and mediate muscle‐brain crosstalk, which plays a crucial role in supporting memory performance [26].

Our MR analyses, which specifically examined memory apart from broader cognitive performance, did not identify any causal associations between sarcopenia‐related traits and memory, except for a positive association between walking pace and working memory. Notably, the associations of sarcopenia‐related traits with memory have not been explored in previous MR studies, all of which have exclusively focused on cognitive performance. A MR study found that higher genetically proxied lean mass was associated with improved cognitive performance [27]. Another MR study identified a causal association of higher walking speed, ALM, and hand grip strength with better cognitive performance [28], while the other MR study showed positive causal associations for walking pace and ALM, but not for grip strength, with cognitive performance [29]. We found the beneficial effect of faster walking pace on working memory, which might be explained by improved cerebral perfusion and proliferation, differentiation, and survival of neurons in the hippocampus area due to physical activity [30, 31]. Moreover, walking pace was assessed using a self‐reported categorical measure (“slow,” “steady,” or “brisk”), which may be subject to subjective bias, as individuals can differ in their perception of these categories depending on age, fitness level, and health status. Such variability may introduce nondifferential misclassification of the exposure. In MR, this type of measurement error is expected to attenuate estimates toward the null, suggesting that the true protective effect of faster walking pace on working memory may be stronger than what was observed. Although we found no genetic associations of other sarcopenia‐related traits with memory in western populations, further research on Chinese and Asian populations is warranted; the adverse associations of sarcopenia with memory function and memory decline observed in our observational study cannot be overlooked.

We have found quite robust evidence for the adverse effect of SO on memory function and memory decline. Previously, these associations were inconclusive. Two Singaporean studies [32, 33], one Chinese study [34] and one American study [35] found that SO was associated with reduced performance in memory, while another Singaporean study showed that SO was not associated with memory impairment [36]. However, three of them were on patients with type 2 diabetes [32, 33] or Alzheimer's disease [34], and four of them were cross‐sectional [32, 33, 34, 36]. Our study can strengthen and extend the existing evidence to general populations using different SO definitions and both cross‐sectional and longitudinal analyses. Moreover, although there existed loss to follow‐up, baseline comparisons showed that individuals who did not return for follow‐up were generally older, socioeconomically disadvantaged, had unhealthier lifestyles, higher levels of obesity and poorer baseline health, suggesting that any follow‐up bias would likely attenuate, rather than exaggerate, the longitudinal associations.

We have further shown that across all SO definitions, SO defined using WHHR showed the strongest and most consistent associations with memory outcomes. WHHR is a central obesity indicator that incorporates waist, hip and height, thereby capturing body fat distribution/central adiposity more comprehensively than general adiposity measures alone. Given its simplicity and reliance on routine anthropometry, WHHR may have practical clinical utility for identifying individuals with sarcopenia and adverse fat distribution who may warrant closer monitoring and targeted intervention. Moreover, although an optimal WHHR cutoff was identified through ROC analysis, this threshold should be interpreted cautiously. Cutoff values may vary across populations, age ranges, and clinical settings, thus proposed cutoffs require external validation across populations and settings before clinical implementation. These findings could be related to age‐related changes in body fat distribution, as older adults tend to experience increased central adiposity while total body weight remains stable [37]. This shift implies that general and central obesity do not always change concurrently, and relying solely on general obesity may underestimate the adverse health effect of obesity and SO. Moreover, no clear interaction between sarcopenia and obesity on memory was found, highlighting the need for close monitoring of sarcopenia in older people, regardless of their obesity status.

Our study had several limitations. First, in GBCS, memory function was assessed using DWRT, rather than a comprehensive battery of cognitive tests. However, a full cognitive assessment is often impractical in large population‐based studies. The DWRT is straightforward and time‐efficient, and has been validated in developing countries [38], as well as in our previous work [18, 19]. Nonetheless, the DWRT measures only delayed memory and does not capture other memory dimensions such as working memory and episodic memory. Therefore, this single indicator may not fully reflect an individual's memory function, and future studies incorporating multidimensional simple memory assessments are warranted. Second, we focused exclusively on obesity indicators that could be obtained by simple anthropometric examinations or formula transformation. While advanced imaging techniques like CT and MRI can provide more accurate estimates of fat mass and distribution, their application is limited to clinical or laboratory settings and is not feasible for routine obesity assessment [39]. Third, the subgroup analyses were limited by relatively small sample sizes. As a result, the wide and overlapping CIs across subgroups made it difficult to distinguish any clearly high‐risk subgroup. Future studies with larger cohorts are needed to more robustly evaluate potential heterogeneity across subgroups. Fourth, due to the lack of GWAS for clinically defined sarcopenia and SO, we could not directly assess their genetic correlations with memory. Our MR analyses therefore relied on individual sarcopenia‐related traits as proxies, which cannot distinguish between sarcopenia and SO subgroups. Consequently, the MR findings should be interpreted as evidence for the causal relevance of muscle‐related characteristics to memory, rather than for specific sarcopenia or SO phenotypes. Further GWAS and MR studies incorporating clinically defined sarcopenia and SO are needed to enable more precise causal inference. Finally, all participants in our MR study were of European descent, whereas our observational analyses were based on a Chinese population. This ethnic heterogeneity may limit the generalizability of the MR findings to Chinese populations, given potential differences in allele frequencies, linkage disequilibrium structures, and gene–environment interactions across ancestries. Consequently, the causal estimates observed from European GWAS should be interpreted with caution when extrapolated to non‐European populations. Further GWAS and MR studies in Chinese and other non‐European populations are needed to confirm the robustness and trans‐ethnic applicability of our findings.

In conclusion, middle‐aged and older people with sarcopenia and SO showed poorer memory function and faster memory decline, with no clear sarcopenia‐obesity interaction, and SO defined by the central obesity indicator WHHR could be the best for SO definition to predict late‐life memory. Our findings emphasize the need to manage sarcopenia, regardless of obesity status, as well as control central obesity, to potentially mitigate memory decline in later life. Further research on preventive strategies and interventions is needed.

## Author Contributions

All authors have contributed significantly and in keeping with the latest guidelines of the International Committee of Medical Journal Editors. Y.Y.H., W.S.Z., J.W., Y.L.J., K.K.C., T.H.L., and L.X. have made substantial contributions to conception and design, acquisition of funding, data, and interpretation of data; Y.Y.H. and L.X. analyzed the data, Y.Y.H. drafted the article, and L.X., T.H.L., J.W., W.S.Z., Y.L.J., and K.K.C. revised it critically for important intellectual content. All authors read and approved the final manuscript.

## Funding

This work was funded by the National Natural Science Foundation of China (82373661). The Guangzhou Biobank Cohort Study was funded by the University of Hong Kong Foundation for Educational Development and Research (SN/1f/HKUF‐DC; C20400.28505200), the Health Medical Research Fund in Hong Kong (HMRF/13143241), the Guangzhou Public Health Bureau (201102A211004011), the Natural Science Foundation of Guangdong (2018A030313140), Guangzhou Twelfth People's Hospital, the University of Birmingham, UK, School of Public Health, the University of Hong Kong, and Greater Bay Area Public Health Research Collaboration. The funders of the study had no role in the study design; data collection, analysis, and interpretation; or writing of the report.

## Disclosure

Tai Hing Lam is an Editorial Board member of Journal of Diabetes and a co‐author of this article. To minimize bias, he was excluded from all editorial decision‐making related to the acceptance of this article for publication.

## Conflicts of Interest

The authors declare no conflicts of interest.

## Supporting information


**Table S1:** Study details for the genome‐wide association studies of exposures and outcomes.
**Table S2:** Baseline characteristics of the study sample by baseline sarcopenia status.
**Table S3:** Baseline characteristic differences between the lost‐to‐follow‐up and completed‐follow‐up populations.
**Table S4:** Associations of baseline sarcopenia and sarcopenic obesity with memory function at baseline.
**Table S5:** Associations of baseline sarcopenia and sarcopenic obesity with memory function at follow‐up.
**Table S6:** Associations of sarcopenia and sarcopenic obesity with memory function using generalized estimating equation.
**Table S7:** Associations of baseline sarcopenia and sarcopenic obesity with annual change of memory function.
**Table S8:** Associations of baseline sarcopenia and sarcopenic obesity with annual change rate of memory function.
**Table S9:** Associations of baseline sarcopenia and sarcopenic obesity with memory function at baseline by sex.
**Table S10:** Associations of baseline sarcopenia and sarcopenic obesity with memory function at baseline by age.
**Table S11:** Associations of baseline sarcopenia and sarcopenic obesity with memory function at baseline by baseline memory status.
**Table S12:** Associations of baseline sarcopenia and sarcopenic obesity with memory function at follow‐up by sex.
**Table S13:** Associations of baseline sarcopenia and sarcopenic obesity with memory function at follow‐up by age.
**Table S14:** Associations of baseline sarcopenia and sarcopenic obesity with memory function at follow‐up by baseline memory status.
**Table S15:** Associations of baseline sarcopenia and sarcopenic obesity with annual change of memory function by sex.
**Table S16:** Associations of baseline sarcopenia and sarcopenic obesity with annual change of memory function by age.
**Table S17:** Associations of baseline sarcopenia and sarcopenic obesity with annual change of memory function by baseline memory status.
**Table S18:** Associations of baseline sarcopenia and sarcopenic obesity with annual change rate of memory function by sex.
**Table S19:** Associations of baseline sarcopenia and sarcopenic obesity with annual change rate of memory function by age.
**Table S20:** Associations of baseline sarcopenia and sarcopenic obesity with annual change rate of memory function by baseline memory status.
**Figure S1:** Associations of sarcopenia and sarcopenic obesity with memory function at baseline.
**Figure S2:** Associations of sarcopenia and sarcopenic obesity with memory function at follow‐up.
**Figure S3:** Associations of sarcopenia and sarcopenic obesity with mean annual change of memory function.
**Figure S4:** Associations of sarcopenia and sarcopenic obesity with mean annual change rate of memory function.
**Figure S5:** Flowchart showing selection of SNPs related to sarcopenia‐related traits used as instruments in analysis of effects on working memory: (A) Hand grip strength (left) (B) Hand grip strength (right) (C) Muscle weakness (D) Walking pace (E) Appendicular lean mass (F) Whole‐body lean mass.
**Figure S6:** Flowchart showing selection of SNPs related to sarcopenia‐related traits used as instruments in analysis of effects on memory loss: (A) Hand grip strength (left) (B) Hand grip strength (right) (C) Muscle weakness (D) Walking pace (E) Appendicular lean mass (F) Whole‐body lean mass.

## References

[1] S. X. Sui , L. J. Williams , K. L. Holloway‐Kew , N. K. Hyde , and J. A. Pasco , “Skeletal Muscle Health and Cognitive Function: A Narrative Review,” International Journal of Molecular Sciences 22, no. 1 (2020): 255. 33383820 10.3390/ijms22010255 PMC7795998

[2] Y. Yang , M. Xiao , L. Leng , et al., “A Systematic Review and Meta‐Analysis of the Prevalence and Correlation of Mild Cognitive Impairment in Sarcopenia,” Journal of Cachexia, Sarcopenia and Muscle 14, no. 1 (2023): 45–56. 36529141 10.1002/jcsm.13143 PMC9891948

[3] N. Amini , M. Ibn Hach , L. Lapauw , et al., “Meta‐Analysis on the Interrelationship Between Sarcopenia and Mild Cognitive Impairment, Alzheimer's Disease and Other Forms of Dementia,” Journal of Cachexia, Sarcopenia and Muscle 15, no. 4 (2024): 1240–1253. 38715252 10.1002/jcsm.13485 PMC11294028

[4] E. Q. Khor , J. P. Lim , L. Tay , et al., “Obesity Definitions in Sarcopenic Obesity: Differences in Prevalence, Agreement and Association With Muscle Function,” Journal of Frailty and Aging 9, no. 1 (2020): 37–43. 32150212 10.14283/jfa.2019.28 PMC12275721

[5] C. R. Liu , P. Y. Wong , Y. L. Chung , et al., “Deciphering the Obesity Paradox in the Elderly: A Systematic Review and Meta‐Analysis of Sarcopenic Obesity,” Obesity Reviews 24, no. 2 (2023): e13534. 36443946 10.1111/obr.13534

[6] S. Eitmann , P. Matrai , P. Hegyi , et al., “Obesity Paradox in Older Sarcopenic Adults ‐ a Delay in Aging: A Systematic Review and Meta‐Analysis,” Ageing Research Reviews 93 (2024): 102164. 38103840 10.1016/j.arr.2023.102164

[7] N. Ludwig , R. T. Hurt , and K. R. Miller , “The Obesity Paradox: Validity and Clinical Implications,” Current Pulmonology Reports 6, no. 1 (2017): 58–63.

[8] G. Livingston , J. Huntley , K. Y. Liu , et al., “Dementia Prevention, Intervention, and Care: 2024 Report of the Lancet Standing Commission,” Lancet 404 (2024): 572–628. 39096926 10.1016/S0140-6736(24)01296-0

[9] D. A. Lawlor , R. M. Harbord , J. A. C. Sterne , N. Timpson , and G. D. Smith , “Mendelian Randomization: Using Genes as Instruments for Making Causal Inferences in Epidemiology,” Statistics in Medicine 27, no. 8 (2008): 1133–1163. 17886233 10.1002/sim.3034

[10] C. Jiang , G. N. Thomas , T. H. Lam , et al., “Cohort Profile: The Guangzhou Biobank Cohort Study, a Guangzhou‐Hong Kong‐Birmingham Collaboration,” International Journal of Epidemiology 35, no. 4 (2006): 844–852. 16844769 10.1093/ije/dyl131

[11] L. Xu , T. H. Lam , C. Q. Jiang , et al., “Adiposity and Incident Diabetes Within 4 Years of Follow‐Up: The Guangzhou Biobank Cohort Study,” Diabetic Medicine 34, no. 10 (2017): 1400–1406. 28477424 10.1111/dme.13378

[12] T. Lu , W. Zhang , C. Jiang , et al., “Association of Salt Intake With Muscle Strength and Physical Performance in Middle‐Aged to Older Chinese: The Guangzhou Biobank Cohort Study,” Nutrients 15, no. 3 (2023): 516. 36771223 10.3390/nu15030516 PMC9919999

[13] X. Liang , C. Q. Jiang , W. S. Zhang , et al., “Association of a Composite Score of Relative Grip Strength and Timed Up and Go Test With Incident Type 2 Diabetes Mellitus: Guangzhou Biobank Cohort Study,” Aging (Albany NY) 13, no. 14 (2021): 18376–18391. 34273143 10.18632/aging.203285 PMC8351683

[14] Consultation WHOE , “Appropriate Body‐Mass Index for Asian Populations and Its Implications for Policy and Intervention Strategies,” Lancet (London, England) 363, no. 9403 (2004): 157–163. 14726171 10.1016/S0140-6736(03)15268-3

[15] K. G. Alberti , R. H. Eckel , S. M. Grundy , et al., “Harmonizing the Metabolic Syndrome: A Joint Interim Statement of the International Diabetes Federation Task Force on Epidemiology and Prevention; National Heart, Lung, and Blood Institute; American Heart Association; World Heart Federation; International Atherosclerosis Society; and International Association for the Study of Obesity,” Circulation 120, no. 16 (2009): 1640–1645. 19805654 10.1161/CIRCULATIONAHA.109.192644

[16] R. Huxley , W. P. James , F. Barzi , et al., “Ethnic Comparisons of the Cross‐Sectional Relationships Between Measures of Body Size With Diabetes and Hypertension,” Obesity Reviews 9, no. 1 (2008): 53–61. 18307700 10.1111/j.1467-789X.2007.00439.x

[17] L. M. Browning , S. D. Hsieh , and M. Ashwell , “A Systematic Review of Waist‐To‐Height Ratio as a Screening Tool for the Prediction of Cardiovascular Disease and Diabetes: 0.5 Could Be a Suitable Global Boundary Value,” Nutrition Research Reviews 23, no. 2 (2010): 247–269. 20819243 10.1017/S0954422410000144

[18] Y. M. Tian , J. Wang , W. S. Zhang , et al., “Association of Perceived Stress With Memory Decline in Older Chinese: The Guangzhou Biobank Cohort Study,” Journal of Affective Disorders 341 (2023): 256–264. 37634823 10.1016/j.jad.2023.08.122

[19] Y. M. Tian , W. S. Zhang , C. Q. Jiang , et al., “Association of Alcohol Use With Memory Decline in Middle‐Aged and Older Chinese: A Longitudinal Cohort Study,” BMC Psychiatry 22, no. 1 (2022): 673. 36320000 10.1186/s12888-022-04298-z PMC9623936

[20] L. K. Chen , J. Woo , P. Assantachai , et al., “Asian Working Group for Sarcopenia: 2019 Consensus Update on Sarcopenia Diagnosis and Treatment,” Journal of the American Medical Directors Association 21, no. 3 (2020): 300–307.e302. 32033882 10.1016/j.jamda.2019.12.012

[21] H. J. Lee , J. Y. Choi , D. Hong , D. Kim , J. Y. Min , and K. B. Min , “Sex Differences in the Association Between Sarcopenia and Mild Cognitive Impairment in the Older Korean Population,” BMC Geriatrics 23, no. 1 (2023): 332. 37248457 10.1186/s12877-023-03911-4 PMC10227990

[22] Y. C. Lin , Z. J. Chen , H. H. Tung , et al., “Association Between Possible Sarcopenia and Domain‐Specific Cognitive Impairment in Middle‐Aged and Older Adults: Insights From the Gan‐Dau Healthy Longevity Plan,” Experimental Gerontology 194 (2024): 112487. 38879092 10.1016/j.exger.2024.112487

[23] T. Sugimoto , Y. Kuroda , N. Matsumoto , et al., “Cross‐Sectional Associations of Sarcopenia and Its Components With Neuropsychological Performance Among Memory Clinic Patients With Mild Cognitive Impairment and Alzheimer's Disease,” Journal of Frailty and Aging 11, no. 2 (2022): 182–189. 35441196 10.14283/jfa.2022.3

[24] Y. S. Handajani , E. S. Butterfill , A. Hengky , S. P. Sugiyono , V. Lamadong , and Y. Turana , “Sarcopenia and Impairment in Global Cognitive, Delayed Memory, and Olfactory Function, Among Community‐Dwelling Adults, in Jakarta, Indonesia: Active Aging Study,” Tzu Chi Medical Journal 35, no. 2 (2023): 193. 37261297 10.4103/tcmj.tcmj_175_22 PMC10227675

[25] L. Maniscalco , N. Veronese , F. S. Ragusa , et al., “Sarcopenia Using Muscle Mass Prediction Model and Cognitive Impairment: A Longitudinal Analysis From the English Longitudinal Study on Ageing,” Archives of Gerontology and Geriatrics 117 (2024): 105160. 37672877 10.1016/j.archger.2023.105160

[26] B. Arosio , R. Calvani , E. Ferri , et al., “Sarcopenia and Cognitive Decline in Older Adults: Targeting the Muscle‐Brain Axis,” Nutrients 15, no. 8 (2023): 1853. 37111070 10.3390/nu15081853 PMC10142447

[27] I. Daghlas , M. Nassan , and D. Gill , “Genetically Proxied Lean Mass and Risk of Alzheimer's Disease: Mendelian Randomisation Study,” BMJ Medicine 2, no. 1 (2023): e000354. 37564828 10.1136/bmjmed-2022-000354 PMC10410880

[28] C. F. Lu , W. S. Liu , X. M. Cang , et al., “The Bidirectional Associations Between Sarcopenia‐Related Traits and Cognitive Performance,” Scientific Reports 14, no. 1 (2024): 7591. 38555389 10.1038/s41598-024-58416-w PMC10981681

[29] H. Liu , Y. Fan , J. Liang , et al., “A Causal Relationship Between Sarcopenia and Cognitive Impairment: A Mendelian Randomization Study,” PLoS One 19, no. 9 (2024): e0309124. 39240885 10.1371/journal.pone.0309124 PMC11379137

[30] C. M. Stillman , J. Cohen , M. E. Lehman , and K. I. Erickson , “Mediators of Physical Activity on Neurocognitive Function: A Review at Multiple Levels of Analysis,” Frontiers in Human Neuroscience 10 (2016): 626. 28018195 10.3389/fnhum.2016.00626 PMC5161022

[31] M. W. Voss , C. Vivar , A. F. Kramer , and H. van Praag , “Bridging Animal and Human Models of Exercise‐Induced Brain Plasticity,” Trends in Cognitive Sciences 17, no. 10 (2013): 525–544. 24029446 10.1016/j.tics.2013.08.001 PMC4565723

[32] S. Low , T. P. Ng , K. S. Goh , et al., “Reduced Skeletal Muscle Mass to Visceral Fat Area Ratio Is Independently Associated With Reduced Cognitive Function in Type 2 Diabetes Mellitus,” Journal of Diabetes and Its Complications 38, no. 2 (2024): 108672. 38183854 10.1016/j.jdiacomp.2023.108672

[33] S. Low , K. S. Goh , T. P. Ng , et al., “The Prevalence of Sarcopenic Obesity and Its Association With Cognitive Performance in Type 2 Diabetes in Singapore,” Clinical Nutrition 39, no. 7 (2020): 2274–2281. 31744622 10.1016/j.clnu.2019.10.019

[34] X. F. Weng , S. W. Liu , M. Li , et al., “Relationship Between Sarcopenic Obesity and Cognitive Function in Patients With Mild to Moderate Alzheimer's Disease,” Psychogeriatrics 23 (2023): 944–953. 37652079 10.1111/psyg.13015

[35] J. A. Batsis , C. Haudenschild , R. M. Roth , et al., “Incident Impaired Cognitive Function in Sarcopenic Obesity: Data From the National Health and Aging Trends Survey,” Journal of the American Medical Directors Association 22, no. 4 (2021): 865–872.e865. 34248422 10.1016/j.jamda.2020.09.008 PMC8269419

[36] N. X. Tou , S. L. Wee , B. W. J. Pang , et al., “Associations of Fat Mass and Muscle Function but Not Lean Mass With Cognitive Impairment: The Yishun Study,” PLoS One 16, no. 8 (2021): e0256702. 34437646 10.1371/journal.pone.0256702 PMC8389410

[37] Y. Sun , B. Liu , L. G. Snetselaar , et al., “Association of Normal‐Weight Central Obesity With All‐Cause and Cause‐Specific Mortality Among Postmenopausal Women,” JAMA Network Open 2, no. 7 (2019): e197337. 31339542 10.1001/jamanetworkopen.2019.7337 PMC6659146

[38] M. Prince , D. Acosta , H. Chiu , M. Scazufca , M. Varghese , and Dementia Research G , “Dementia Diagnosis in Developing Countries: A Cross‐Cultural Validation Study,” Lancet 361, no. 9361 (2003): 909–917. 12648969 10.1016/S0140-6736(03)12772-9

[39] P. Gonzalez‐Muniesa , M. A. Martinez‐Gonzalez , F. B. Hu , et al., “Obesity,” Nature Reviews Disease Primers 3 (2017): 17034. 10.1038/nrdp.2017.34 28617414

