# Supplementary material for: Associations of Sarcopenia and Sarcopenic Obesity Using Different Obesity Definitions With Memory Decline
Source: J Diabetes. 2026 Mar 12;18(3):e70210. doi: 10.1111/1753-0407.70210 (PMC13098154; doi:10.1111/1753-0407.70210)
Supplement: Supplementary file 1 — Table S1: Study details for the genome‐wide association studies of exposures and outcomes. Table S2: Baseline characteristics of the study sample by baseline sarcopenia status. Table S3: Baseline characteristic differences between the lost‐to‐follow‐up and completed‐follow‐up populations. Table S4: Associations of baseline sarcopenia and sarcopenic obesity with memory function at baseline. Table S5: Associations of baseline sarcopenia and sarcopenic obesity with memory function at follow‐up. Table S6: Associations of sarcopenia and sarcopenic obesity with memory function using generalized estimating equation. Table S7: Associations of baseline sarcopenia and sarcopenic obesity with annual change of memory function. Table S8: Associations of baseline sarcopenia and sarcopenic obesity with annual change rate of memory function. Table S9: Associations of baseline sarcopenia and sarcopenic obesity with memory function at baseline by sex. Table S10: Associations of baseline sarcopenia and sarcopenic obesity with memory function at baseline by age. Table S11: Associations of baseline sarcopenia and sarcopenic obesity with memory function at baseline by baseline memory status. Table S12: Associations of baseline sarcopenia and sarcopenic obesity with memory function at follow‐up by sex. Table S13: Associations of baseline sarcopenia and sarcopenic obesity with memory function at follow‐up by age. Table S14: Associations of baseline sarcopenia and sarcopenic obesity with memory function at follow‐up by baseline memory status. Table S15: Associations of baseline sarcopenia and sarcopenic obesity with annual change of memory function by sex. Table S16: Associations of baseline sarcopenia and sarcopenic obesity with annual change of memory function by age. Table S17: Associations of baseline sarcopenia and sarcopenic obesity with annual change of memory function by baseline memory status. Table S18: Associations of baseline sarcopenia and sarcopenic obesity with annual change [file JDB-18-e70210-s001.docx]

**Supplementary Methods**

*Exposures*

We searched PubMed in title and abstract using keywords of “obesity indicators”, “obesity indices”, “obesity score”, “obesity markers” or “obesity index” up to 23^rd^ January 2024 and identified 1,487 studies. Generally, several indicators have been used to define obesity in the literature, including traditional obesity indicators such as body mass index (BMI), waist circumference (WC) and waist-to-hip ratio (WHR), and novel obesity indicators such as a body shape index (ABSI), lipid accumulation product (LAP) and visceral adiposity index (VAI). Among these indicators, some are obtained by simple anthropometric examinations, some are measured by CT scanning, bioelectrical impedance analysis or dual-energy X-ray absorptiometry, and some are calculated by formula transformation. Specifically, weight, WC, hip circumference (HC), abdominal circumference, neck circumference and calf circumference are the first category, fat mass and body fat percentage are the second category, and BMI, WHR, ABSI, LAP and VAI are the third category. In our study, we would focus on obesity indicators that are simple and easy to generalise broadly, i.e., category I and category III obesity indicators. Guangzhou Biobank Cohort Study (GBCS) included a face-to-face interview by trained nurses using a computer-assisted standardised questionnaire that included demographic characteristics, and assessment of anthropometric parameters and lipids. And anthropometric measures were measured with light indoor clothing and no shoes according to a standard protocol. Height was measured to the nearest 0.1 cm and weight was measured to the nearest 0.1 kg. WC was measured to the nearest 0.1 cm, using a non-stretch tape horizontally around the narrowest part of the torso between the lowest rib and the iliac crest, and HC was measured around the maximal girth of the hips. After careful review, the following 20 indicators could be used in our study: weight, BMI, WC, HC, WHR, waist-to-height ratio (WHtR), LAP (1), ABSI (2), VAI (3), Chinese VAI (CVAI) (4), body roundness index (BRI) (5), conicity index (6), body adiposity index (BAI) (7), cardiometabolic index (CMI) (8), body surface area (BSA) (9), waist-to-hip-to-height ratio (WHHR), predicted fat mass (PFM) (10), predicted lean mass (PLM) (10), predicated percent fat (PPF) (10) and Clínica Universidad de Navarra-Body Adiposity Estimator (CUN-BAE) (11). Except for weight, WC and HC, which could be obtained directly from anthropometric examinations, other indicators would be calculated as follows:

$$BMI=\frac{weight (kg)}{{height (m)}^{2}}$$

$$WHR=\frac{WC (cm)}{HC (cm)}$$

$$WHtR=\frac{WC (cm)}{height (cm)}$$

$${LAP}_{men}=\left[ WC \left( cm \right)-65 \right]\times TG \left( {mmol}/l \right)$$

$${LAP}_{women}=[WC (cm)-58]\times TG (mmol/l)$$

$$ABSI=\frac{WC (m)}{{BMI}^{\frac{2}{3}}\times{height \left( m \right)}^{\frac{1}{2}}}$$

$${VAI}_{men}=(\frac{WC \left( cm \right)}{39.68+1.88\times BMI})\times(\frac{TG \left（ {mmol}/l \right）}{1.03})\times(\frac{1.31}{HDL \left（ {mmol}/l \right）})$$

$${VAI}_{women}=(\frac{WC \left( cm \right)}{36.58+1.89\times BMI})\times(\frac{TG \left（ {mmol}/l \right）}{0.81})\times(\frac{1.52}{HDL \left（ {mmol}/l \right）})$$

$${CVAI}_{men}=-267.93+0.69\times age\left( y \right)+0.03\times BMI+4.00\times WC \left( cm \right)+22.00\times Lg TG \left（ {mmol}/l \right）-16.32\times HDL \left（ {mmol}/l \right）$$

$${CVAI}_{women}=-187.32+1.71\times age\left( y \right)+4.32\times BMI+1.12\times WC \left( cm \right)+39.76\times Lg TG \left（ {mmol}/l \right）-11.66\times HDL \left（ {mmol}/l \right）$$

$$BRI=364.2-365.5\times\left\{ 1-\frac{{[WC {(m)}/{2\pi}]}^{2}}{{[0.5\times height \left( m \right)]}^{2}} \right\}^{\frac{1}{2}}$$

$$conicity index=\frac{WC (m)}{0.109\times\surd\frac{weight (kg)}{height (m)}}$$

$$BAI=\frac{HC (cm)}{{height (m)}^{1.5}}-18$$

$$CMI=\frac{TG \left（ {mmol}/l \right）}{HDL \left（ {mmol}/l \right）}\times WHtR$$

$$BSA=0.007184\times{height (cm)}^{0.725}\times{weight (kg)}^{0.425}$$

$$WHHR=\frac{WHR}{height (cm)}$$

$${PFM}_{men}=-18.592-0.009\times age \left( y \right)-0.080\times height \left( cm \right)+0.226\times weight \left( kg \right)+0.387\times WC \left( cm \right)+0.080\times Mexican-0.188\times Hispanic-0.483\times Black+1.050\times other ethnicity$$

$${PFM}_{women}=11.817+0.041\times age \left( y \right)-0.199\times height \left( cm \right)+0.610\times weight \left( kg \right)+0.044\times WC \left( cm \right)+0.388\times Mexican+0.073\times Hispanic-1.187\times Black+0.325\times other ethnicity$$

$${PLM}_{men}=19.363+0.001\times age \left( y \right)+0.064\times height \left( cm \right)+0.756\times weight \left( kg \right)-0.366\times WC \left( cm \right)-0.066\times Mexican+0.231\times Hispanic+0.432\times Black-1.007\times other ethnicity$$

$${PLM}_{women}=-10.683-0.039\times age \left( y \right)+0.186\times height \left( cm \right)+0.383\times weight \left( kg \right)-0.043\times WC \left( cm \right)-0.359\times Mexican-0.059\times Hispanic+1.085\times Black-0.34\times other ethnicity$$

$${PPF}_{men}=0.02+0.00\times age \left( y \right)-0.07\times height \left( cm \right)-0.08\times weight \left( kg \right)+0.48\times WC \left( cm \right)+0.32\times Mexican+0.02\times Hispanic-0.65\times Black+1.12\times other ethnicity$$

$${PPF}_{women}=50.46+0.07\times age \left( y \right)-0.26\times height \left( cm \right)+0.27\times weight \left( kg \right)+0.10\times WC \left( cm \right)+0.89\times Mexican+0.49\times Hispanic-1.57\times Black+0.43\times other ethnicity$$

$$CUN-BAE=-44.988+\left[ 0.503\times age \left( y \right) \right]+\left( 10.689\times sex \right)+\left( 3.172\times BMI \right)-\left( 0.026\times{BMI}^{2} \right)+\left( 0.181\times BMI\times sex \right)-\left[ 0.02\times BMI\times age \left( y \right) \right]-\left( 0.005\times{BMI}^{2}\times sex \right)+[0.00021\times{BMI}^{2}\times age \left( y \right)]$$

Note: HDL: high-density lipoprotein; TG: triglyceride; men=0 and women=1 for sex

**References**

1. Kahn HS. The "lipid accumulation product" performs better than the body mass index for recognizing cardiovascular risk: a population-based comparison. BMC Cardiovasc Disord. 2005;5:26.

2. Krakauer NY, Krakauer JC. A new body shape index predicts mortality hazard independently of body mass index. PLoS One. 2012;7(7):e39504.

3. Amato MC, Giordano C, Galia M, Criscimanna A, Vitabile S, Midiri M, et al. Visceral Adiposity Index: a reliable indicator of visceral fat function associated with cardiometabolic risk. Diabetes Care. 2010;33(4):920-922.

4. Xia MF, Chen Y, Lin HD, Ma H, Li XM, Aleteng Q, et al. A indicator of visceral adipose dysfunction to evaluate metabolic health in adult Chinese. Sci Rep. 2016;6:38214.

5. Thomas DM, Bredlau C, Bosy-Westphal A, Mueller M, Shen W, Gallagher D, et al. Relationships between body roundness with body fat and visceral adipose tissue emerging from a new geometrical model. Obesity (Silver Spring). 2013;21(11):2264-2271.

6. Valdez R, Seidell JC, Ahn YI, Weiss KM. A new index of abdominal adiposity as an indicator of risk for cardiovascular disease. A cross-population study. Int J Obes Relat Metab Disord. 1993;17(2):77-82.

7. Bergman RN, Stefanovski D, Buchanan TA, Sumner AE, Reynolds JC, Sebring NG, et al. A better index of body adiposity. Obesity (Silver Spring). 2011;19(5):1083-1089.

8. Wakabayashi I, Daimon T. The "cardiometabolic index" as a new marker determined by adiposity and blood lipids for discrimination of diabetes mellitus. Clin Chim Acta. 2015;438:274-278.

9. Du Bois D, Du Bois EF. A formula to estimate the approximate surface area if height and weight be known. 1916. Nutrition. 1989;5(5):303-311; discussion 312-313.

10. Lee DH, Keum N, Hu FB, Orav EJ, Rimm EB, Sun Q, et al. Development and validation of anthropometric prediction equations for lean body mass, fat mass and percent fat in adults using the National Health and Nutrition Examination Survey (NHANES) 1999-2006. Br J Nutr. 2017;118(10):858-866.

11. Gomez-Ambrosi J, Silva C, Galofre JC, Escalada J, Santos S, Millan D, et al. Body mass index classification misses subjects with increased cardiometabolic risk factors related to elevated adiposity. Int J Obes (Lond). 2012;36(2):286-294.

Table S1. Study details for the genome-wide association studies of exposures and outcomes.

| **Phenotype** | **Definition** | **Unit** | **Year** | **Consortium** | **Sample size** | **Mean age** | **Adjust** | **Pubmed ID** | **GWAS ID** |
| --- | --- | --- | --- | --- | --- | --- | --- | --- | --- |
| **Exposures** | | | | | | | | |  |
| Hand grip strength (left) | - | SD kg | 2018 | UK Biobank | 461,026 | 40-69 years | - | - | ukb-b-7478 |
| Hand grip strength (right) | - | SD kg | 2018 | UK Biobank | 461,089 | 40-69 years | - | - | ukb-b-10215 |
| Muscle weakness | Grip strength <30 kg Male; <20 kg Female | - | 2021 | 22 independent cohorts, including UK Biobank, US Health and Retirement Study, Framingham Heart Study, and others | 256,523 | 60 years and over | age, sex, and technical covariates | 33510174 | ebi-a-GCST90007526 |
| Walking pace | “How would you describe your usual walking pace?” (The responses “slow”, “steady/average” and “brisk” were coded as 0, 1 and 2) | - | 2020 | UK Biobank | 450,967 | 40-69 years | age, sex, genotyping array and 20 PCs | 33128006 | https://doi.org/10.6084/m9.figshare.12967088.v1 |
| Appendicular lean mass | The sum of fat-free mass at the arms and legs | SD kg | 2020 | UK Biobank | 450,243 | 48-73 years | AFM, age, age squared, the top 10 PCs, assessment centre (23 levels) and genotyping array (2 levels) | 33097823 | ebi-a-GCST90000025 |
| Whole-body lean mass | The sum of whole-body fat-free mass | SD kg | 2018 | UK Biobank | 454,850 | 40-69 years | - | - | ukb-b-13354 |
| **Outcomes** | | | | | | | | |  |
| Working memory | A numeric n-back task with the primary outcome d’ | - | 2020 | ALSPAC | 2,471 | 24 years | - | - | https://doi.org/10.5523/bris.2ux5exb501kds2pq7wv8o6dv85 |
| Memory loss | - | - | 2024 | FinnGen | 453,733 | 63 years | - | 36653562 | The FinnGen database (https://storage.googleapis.com/finngen-public-data-r11/summary_stats/finngen_R11_MEMLOSS.gz) |

ALSPAC, Age 24 Clinic of the Avon Longitudinal Study of Parents and Children; AFM, appendicular fat mass; PC, principal component; SD, standard deviation

Table S2. Baseline characteristics of the study sample by baseline sarcopenia status.

|  | Cross-sectional analyses | | | |  | Longitudinal analyses | | | |
| --- | --- | --- | --- | --- | --- | --- | --- | --- | --- |
|  | Total | Sarcopenia status | | P-value |  | Total | Sarcopenia status | | P-value |
|  |  | No | Yes |  |  |  | No | Yes |  |
| Number of participants (row percentage %) | 6,390 (100.00) | 2,626 (41.10) | 3,764 (58.90) |  |  | 3,979 (100.00) | 1,800 (45.24) | 2,179 (54.76) |  |
| Sex (%) |  |  |  |  |  |  |  |  |  |
| Men | 25.02 | 26.35 | 24.10 | 0.04 |  | 24.98 | 26.67 | 23.59 | 0.03 |
| Women | 74.98 | 73.65 | 75.90 |  |  | 75.02 | 73.33 | 76.41 |  |
| Age, years, mean (SD) | 60.53 (7.75) | 57.47 (5.68) | 62.67 (8.26) | <0.001 |  | 59.43 (7.19) | 57.18 (5.52) | 61.30 (7.84) | <0.001 |
| Education (%) |  |  |  |  |  |  |  |  |  |
| Primary or below | 37.70 | 24.60 | 46.84 | <0.001 |  | 32.52 | 22.83 | 40.52 | <0.001 |
| Secondary | 53.62 | 64.89 | 45.75 |  |  | 58.23 | 66.94 | 51.03 |  |
| College or above | 8.69 | 10.51 | 7.41 |  |  | 9.25 | 10.22 | 8.44 |  |
| Occupation (%) |  |  |  |  |  |  |  |  |  |
| Manual | 61.47 | 57.12 | 64.51 | <0.001 |  | 60.82 | 57.72 | 63.38 | <0.001 |
| Non-manual | 20.94 | 24.45 | 18.49 |  |  | 21.64 | 24.17 | 19.55 |  |
| Others | 17.59 | 18.43 | 17.00 |  |  | 17.54 | 18.11 | 17.07 |  |
| Personal income (%)^a^ |  |  |  |  |  |  |  |  |  |
| <10,000 RMB/year | 27.92 | 21.55 | 32.36 | <0.001 |  | 26.01 | 20.89 | 30.24 | <0.001 |
| 10,000-14,999 RMB/year | 48.79 | 50.69 | 47.48 |  |  | 50.29 | 51.78 | 49.06 |  |
| ≥15,000 RMB/year | 19.61 | 23.80 | 16.68 |  |  | 19.98 | 23.56 | 17.03 |  |
| Not reported | 3.68 | 3.96 | 3.48 |  |  | 3.72 | 3.78 | 3.67 |  |
| Smoking status (%) |  |  |  |  |  |  |  |  |  |
| Never | 82.30 | 83.47 | 81.48 | 0.12 |  | 83.49 | 83.17 | 83.75 | 0.86 |
| Former | 7.54 | 7.08 | 7.86 |  |  | 7.21 | 7.44 | 7.02 |  |
| Current | 10.16 | 9.44 | 10.65 |  |  | 9.30 | 9.39 | 9.22 |  |
| Alcohol drinking (%) |  |  |  |  |  |  |  |  |  |
| Never | 50.66 | 45.89 | 53.99 | <0.001 |  | 49.23 | 45.50 | 52.32 | <0.001 |
| Former | 6.32 | 6.63 | 6.11 |  |  | 6.36 | 6.61 | 6.15 |  |
| Current | 43.03 | 47.49 | 39.90 |  |  | 44.41 | 47.89 | 41.53 |  |
| Physical activity (%) |  |  |  |  |  |  |  |  |  |
| Inactive | 6.95 | 6.09 | 7.55 | <0.001 |  | 7.09 | 6.28 | 7.76 | 0.06 |
| Moderate | 28.75 | 26.43 | 30.37 |  |  | 27.97 | 27.06 | 28.73 |  |
| Active | 64.30 | 67.48 | 62.09 |  |  | 64.94 | 66.67 | 63.52 |  |
| Self-rated health (%) |  |  |  |  |  |  |  |  |  |
| Poor | 20.38 | 15.99 | 23.43 | <0.001 |  | 19.08 | 15.44 | 22.07 | <0.001 |
| Good | 79.62 | 84.01 | 76.57 |  |  | 80.92 | 84.56 | 77.93 |  |
| Weight, kg, mean (SD) | 58.64 (9.63) | 59.02 (9.14) | 58.37 (9.95) | 0.007 |  | 58.92 (9.59) | 59.10 (9.24) | 58.76 (9.87) | 0.27 |
| BMI, kg/m^2^, mean (SD) | 23.84 (3.30) | 23.56 (3.02) | 24.04 (3.47) | <0.001 |  | 23.88 (3.26) | 23.56 (3.01) | 24.14 (3.43) | <0.001 |
| WC, cm, mean (SD) | 77.52 (9.08) | 76.46 (8.50) | 78.26 (9.40) | <0.001 |  | 77.19 (8.91) | 76.30 (8.41) | 77.93 (9.25) | <0.001 |
| HC, cm, mean (SD) | 89.62 (6.24) | 89.65 (5.81) | 89.60 (6.53) | 0.77 |  | 89.70 (6.15) | 89.67 (5.83) | 89.73 (6.41) | 0.76 |
| WHR, mean (SD) | 0.86 (0.07) | 0.85 (0.07) | 0.87 (0.07) | <0.001 |  | 0.86 (0.07) | 0.85 (0.07) | 0.87 (0.07) | <0.001 |
| WHtR, mean (SD) | 0.50 (0.06) | 0.48 (0.05) | 0.50 (0.06) | <0.001 |  | 0.49 (0.06) | 0.48 (0.05) | 0.50 (0.06) | <0.001 |
| LAP,^b^ mean (SD) | 34.69 (33.11) | 31.79 (30.88) | 36.72 (34.45) | <0.001 |  | 33.91 (31.77) | 31.55 (30.89) | 35.87 (32.35) | <0.001 |
| ABSI, mean (SD) | 0.07 (0.005) | 0.07 (0.005) | 0.08 (0.005) | <0.001 |  | 0.07 (0.005) | 0.074 (0.005) | 0.075 (0.005) | <0.001 |
| VAI, mean (SD) | 2.01 (2.10) | 1.93 (1.99) | 2.06 (2.17) | 0.01 |  | 1.98 (1.96) | 1.91 (1.86) | 2.04 (2.04) | 0.03 |
| CVAI, mean (SD) | 94.60 (42.36) | 86.23 (39.02) | 100.44 (43.61) | <0.001 |  | 92.53 (41.55) | 85.44 (39.14) | 98.40 (42.55) | <0.001 |
| BRI, mean (SD) | 3.33 (1.09) | 3.11 (0.96) | 3.49 (1.15) | <0.001 |  | 3.28 (1.05) | 3.09 (0.93) | 3.43 (1.11) | <0.001 |
| Conicity index, mean (SD) | 0.15 (0.01) | 0.15 (0.01) | 0.16 (0.02) | <0.001 |  | 0.15 (0.01) | 0.150 (0.01) | 0.154 (0.02) | <0.001 |
| BAI, mean (SD) | 27.82 (3.95) | 27.19 (3.62) | 28.27 (4.10) | <0.001 |  | 27.76 (3.88) | 27.17 (3.62) | 28.24 (4.02) | <0.001 |
| CMI, mean (SD) | 0.63 (0.69) | 0.60 (0.65) | 0.65 (0.72) | 0.007 |  | 0.62 (0.64) | 0.59 (0.60) | 0.65 (0.67) | 0.01 |
| BSA, mean (SD) | 1.58 (0.15) | 1.59 (0.14) | 1.57 (0.15) | <0.001 |  | 1.58 (0.14) | 1.60 (0.14) | 1.57 (0.15) | <0.001 |
| WHHR, mean (SD) | 0.006 (0.0005) | 0.005 (0.0004) | 0.006 (0.0005) | <0.001 |  | 0.005 (0.0005) | 0.005 (0.0004) | 0.006 (0.0005) | <0.001 |
| PFM, mean (SD) | 20.08 (6.30) | 19.70 (5.91) | 20.35 (6.55) | <0.001 |  | 20.12 (6.19) | 19.66 (5.89) | 20.49 (6.41) | <0.001 |
| PLM, mean (SD) | 37.29 (7.35) | 38.03 (7.37) | 36.77 (7.29) | <0.001 |  | 37.53 (7.44) | 38.14 (7.49) | 37.03 (7.35) | <0.001 |
| PPF, mean (SD) | 34.31 (7.17) | 33.53 (6.99) | 34.84 (7.25) | <0.001 |  | 34.20 (7.09) | 33.45 (6.99) | 34.82 (7.11) | <0.001 |
| CUN-BAE, mean (SD) | 33.60 (6.74) | 32.65 (6.49) | 34.27 (6.82) | <0.001 |  | 33.49 (6.65) | 32.57 (6.50) | 34.26 (6.68) | <0.001 |
| DWRT score, mean (SD) | 5.92 (1.97) | 6.34 (1.81) | 5.63 (2.03) | <0.001 |  | 6.13 (1.89) | 6.43 (1.77) | 5.87 (1.94) | <0.001 |

ABSI: a body shape index; BAI: body adiposity index; BMI: body mass index; BRI: body roundness index; BSA: body surface area; CMI: cardiometabolic index; CUN-BAE: Clínica Universidad de Navarra-Body Adiposity Estimator; CVAI: Chinese visceral adiposity index; DWRT: Delayed Word Recall Test; HC, hip circumference; LAP: lipid accumulation product; PFM: predicted fat mass; PLM: predicted lean mass; PPF: predicated percent fat; SD, standard deviation; VAI: visceral adiposity index; WC: waist circumference; WHHR: waist-to-hip-to-height ratio; WHR: waist-to-hip ratio; WHtR: waist-to-height ratio

^a^: US $1 ≈ 8 RMB

^b^: Only 6,280 and 3,913 participants with LAP greater than or equal to zero were included in the cross-sectional and longitudinal data, respectively

Table S3. Baseline characteristic differences between the lost-to-follow-up and completed-follow-up populations.

|  | Total | Completed-follow-up | Lost-to-follow-up | P-value |
| --- | --- | --- | --- | --- |
| Number of participants (row percentage %) | 6,390 (100.00) | 3,979 (62.27) | 2,411 (37.73) |  |
| Sex (%) |  |  |  |  |
| Men | 25.02 | 24.98 | 25.09 | 0.92 |
| Women | 74.98 | 75.01 | 74.91 |  |
| Age, years, mean (SD) | 60.53 (7.75) | 59.43 (7.19) | 62.35 (8.29) | <0.001 |
| Education (%) |  |  |  |  |
| Primary or below | 37.70 | 32.52 | 46.25 | <0.001 |
| Secondary | 53.62 | 58.23 | 46.00 |  |
| College or above | 8.69 | 9.25 | 7.76 |  |
| Occupation (%) |  |  |  |  |
| Manual | 61.47 | 60.82 | 62.55 | 0.20 |
| Non-manual | 20.94 | 21.64 | 19.78 |  |
| Others | 17.59 | 17.54 | 17.67 |  |
| Personal income (%)^a^ |  |  |  |  |
| <10,000 RMB/year | 27.92 | 26.01 | 31.07 | <0.001 |
| 10,000-14,999 RMB/year | 48.79 | 50.29 | 46.33 |  |
| ≥15,000 RMB/year | 19.61 | 19.98 | 19.00 |  |
| Not reported | 3.68 | 3.72 | 3.61 |  |
| Smoking status (%) |  |  |  |  |
| Never | 82.30 | 83.49 | 80.34 | 0.004 |
| Former | 7.54 | 7.21 | 8.09 |  |
| Current | 10.16 | 9.30 | 11.57 |  |
| Alcohol drinking (%) |  |  |  |  |
| Never | 50.66 | 49.23 | 52.01 | 0.01 |
| Former | 6.32 | 6.36 | 6.26 |  |
| Current | 43.03 | 44.41 | 40.73 |  |
| Physical activity (%) |  |  |  |  |
| Inactive | 6.95 | 7.09 | 6.72 | 0.21 |
| Moderate | 28.75 | 27.97 | 30.03 |  |
| Active | 64.30 | 64.94 | 63.25 |  |
| Self-rated health (%) |  |  |  |  |
| Poor | 20.38 | 19.08 | 22.52 | 0.001 |
| Good | 79.62 | 80.92 | 77.48 |  |
| Weight, kg, mean (SD) | 58.64 (9.63) | 58.92 (9.59) | 58.17 (9.67) | 0.65 |
| BMI, kg/m^2^, mean (SD) | 23.84 (3.30) | 23.88 (3.26) | 23.78 (3.38) | 0.05 |
| WC, cm, mean (SD) | 77.52 (9.08) | 77.19 (8.91) | 78.06 (9.33) | 0.01 |
| HC, cm, mean (SD) | 89.62 (6.24) | 89.70 (6.15) | 89.49 (6.39) | 0.04 |
| WHR, mean (SD) | 0.86 (0.07) | 0.86 (0.07) | 0.87 (0.07) | 0.04 |
| WHtR, mean (SD) | 0.50 (0.06) | 0.49 (0.06) | 0.50 (0.06) | <0.001 |
| LAP,^b^ mean (SD) | 34.69 (33.11) | 33.91 (31.77) | 35.97 (35.19) | <0.001 |
| ABSI, mean (SD) | 0.07 (0.005) | 0.07 (0.005) | 0.08 (0.005) | 0.003 |
| VAI, mean (SD) | 2.01 (2.10) | 1.98 (1.96) | 2.07 (2.31) | <0.001 |
| CVAI, mean (SD) | 94.60 (42.36) | 92.53 (41.55) | 98.02 (43.46) | 0.01 |
| BRI, mean (SD) | 3.33 (1.09) | 3.28 (1.05) | 3.43 (1.16) | <0.001 |
| Conicity index, mean (SD) | 0.15 (0.01) | 0.15 (0.01) | 0.16 (0.02) | <0.001 |
| BAI, mean (SD) | 27.82 (3.95) | 27.76 (3.88) | 27.94 (4.05) | 0.02 |
| CMI, mean (SD) | 0.63 (0.69) | 0.62 (0.64) | 0.65 (0.77) | <0.001 |
| BSA, mean (SD) | 1.58 (0.15) | 1.58 (0.14) | 1.57 (0.15) | 0.85 |
| WHHR, mean (SD) | 0.006 (0.0005) | 0.005 (0.0005) | 0.006 (0.0005) | <0.001 |
| PFM, mean (SD) | 20.08 (6.30) | 20.12 (6.19) | 20.03 (6.49) | 0.01 |
| PLM, mean (SD) | 37.29 (7.35) | 37.53 (7.44) | 36.89 (7.19) | 0.07 |
| PPF, mean (SD) | 34.31 (7.17) | 34.20 (7.09) | 34.48 (7.30) | 0.11 |
| CUN-BAE, mean (SD) | 33.60 (6.74) | 33.49 (6.65) | 33.78 (6.87) | 0.08 |
| DWRT score, mean (SD) | 5.92 (1.97) | 6.13 (1.89) | 5.59 (2.06) | <0.001 |

ABSI: a body shape index; BAI: body adiposity index; BMI: body mass index; BRI: body roundness index; BSA: body surface area; CMI: cardiometabolic index; CUN-BAE: Clínica Universidad de Navarra-Body Adiposity Estimator; CVAI: Chinese visceral adiposity index; DWRT: Delayed Word Recall Test; HC, hip circumference; LAP: lipid accumulation product; PFM: predicted fat mass; PLM: predicted lean mass; PPF: predicated percent fat; SD, standard deviation; VAI: visceral adiposity index; WC: waist circumference; WHHR: waist-to-hip-to-height ratio; WHR: waist-to-hip ratio; WHtR: waist-to-height ratio

^a^: US $1 ≈ 8 RMB

^b^: Only 6,280 participants with LAP greater than or equal to zero were included

Table S4. Associations of baseline sarcopenia and sarcopenic obesity with memory function at baseline.

|  | *N* | Adjusted mean differences β (95% CI) in baseline DWRT score | | |
| --- | --- | --- | --- | --- |
|  |  | Model 1^a^ | Model 2^b^ | Model 3^c^ |
| Sarcopenia |  |  |  |  |
| No | 2,626 | Ref. (0) | Ref. (0) | Ref. (0) |
| Yes | 3,764 | -0.71 (-0.80 to -0.61)^***^ | -0.41 (-0.51 to -0.31)^***^ | -0.24 (-0.33 to -0.14)^***^ |
| Sarcopenic obesity (Weight) |  |  |  |  |
| Without sarcopenia and obesity | 1,762 | Ref. (0) | Ref. (0) | Ref. (0) |
| Only with sarcopenia | 2,510 | -0.78 (-0.90 to -0.66)^***^ | -0.45 (-0.57 to -0.33)^***^ | -0.26 (-0.37 to -0.14)^***^ |
| Only with obesity | 864 | 0.004 (-0.15 to 0.16) | -0.02 (-0.18 to 0.13) | 0.004 (-0.14 to 0.15) |
| Sarcopenic obesity | 1,254 | -0.56 (-0.70 to -0.42)^***^ | -0.36 (-0.50 to -0.22)^***^ | -0.20 (-0.33 to -0.06)^**^ |
| P for interaction |  | 0.04 | 0.27 | 0.56 |
| Sarcopenic obesity (BMI) |  |  |  |  |
| Without sarcopenia and obesity | 1,832 | Ref. (0) | Ref. (0) | Ref. (0) |
| Only with sarcopenia | 2,359 | -0.74 (-0.86 to -0.62)^***^ | -0.42 (-0.54 to -0.30)^***^ | -0.24 (-0.35 to -0.12)^***^ |
| Only with obesity | 794 | -0.07 (-0.23 to 0.09) | -0.06 (-0.22 to 0.10) | -0.001 (-0.15 to 0.15) |
| Sarcopenic obesity | 1,405 | -0.72 (-0.85 to -0.58)^***^ | -0.444 (-0.58 to -0.31)^***^ | -0.23 (-0.36 to -0.10)^**^ |
| P for interaction |  | 0.38 | 0.73 | 0.97 |
| Sarcopenic obesity (WC) |  |  |  |  |
| Without sarcopenia and obesity | 1,988 | Ref. (0) | Ref. (0) | Ref. (0) |
| Only with sarcopenia | 2,500 | -0.70 (-0.81 to -0.58)^***^ | -0.42 (-0.53 to -0.30)^***^ | -0.24 (-0.35 to -0.13)^***^ |
| Only with obesity | 638 | -0.12 (-0.29 to 0.05) | -0.13 (-0.30 to 0.04) | -0.0002 (-0.16 to 0.16) |
| Sarcopenic obesity | 1,264 | -0.82 (-0.96 to -0.68)^***^ | -0.51 (-0.65 to -0.37)^***^ | -0.23 (-0.36 to -0.09)^**^ |
| P for interaction |  | 0.96 | 0.70 | 0.89 |
| Sarcopenic obesity (HC) |  |  |  |  |
| Without sarcopenia and obesity | 1,875 | Ref. (0) | Ref. (0) | Ref. (0) |
| Only with sarcopenia | 2,611 | -0.76 (-0.87 to -0.64)^***^ | -0.45 (-0.56 to -0.33)^***^ | -0.25 (-0.37 to -0.14)^***^ |
| Only with obesity | 751 | -0.02 (-0.19 to 0.14) | -0.05 (-0.21 to 0.11) | -0.0005 (-0.15 to 0.15) |
| Sarcopenic obesity | 1,153 | -0.62 (-0.77 to -0.48)^***^ | -0.39 (-0.53 to -0.24)^***^ | -0.20 (-0.34 to -0.06)^**^ |
| P for interaction |  | 0.15 | 0.28 | 0.60 |
| Sarcopenic obesity (WHR) |  |  |  |  |
| Without sarcopenia and obesity | 931 | Ref. (0) | Ref. (0) | Ref. (0) |
| Only with sarcopenia | 966 | -0.61 (-0.79 to -0.44)^***^ | -0.38 (-0.55 to -0.20)^***^ | -0.23 (-0.39 to -0.06)^*^ |
| Only with obesity | 1,695 | -0.16 (-0.32 to -0.01)^*^ | -0.20 (-0.35 to -0.05)^*^ | -0.13 (-0.28 to 0.02) |
| Sarcopenic obesity | 2,798 | -0.88 (-1.03 to -0.74)^***^ | -0.61 (-0.76 to -0.46)^***^ | -0.36 (-0.51 to -0.21)^***^ |
| P for interaction |  | 0.33 | 0.72 | 0.98 |
| Sarcopenic obesity (WHtR) |  |  |  |  |
| Without sarcopenia and obesity | 1,659 | Ref. (0) | Ref. (0) | Ref. (0) |
| Only with sarcopenia | 1,858 | -0.65 (-0.78 to -0.52)^***^ | -0.40 (-0.53 to -0.27)^***^ | -0.23 (-0.35 to -0.10)^**^ |
| Only with obesity | 967 | -0.28 (-0.43 to -0.13)^**^ | -0.19 (-0.34 to -0.04)^*^ | -0.06 (-0.21 to 0.08) |
| Sarcopenic obesity | 1,906 | -0.97 (-1.10 to -0.84)^***^ | -0.58 (-0.71 to -0.45)^***^ | -0.30 (-0.43 to -0.17)^***^ |
| P for interaction |  | 0.66 | 0.91 | 0.91 |
| Sarcopenic obesity (LAP) |  |  |  |  |
| Without sarcopenia and obesity | 1,857 | Ref. (0) | Ref. (0) | Ref. (0) |
| Only with sarcopenia | 2,404 | -0.68 (-0.80 to -0.57)^***^ | -0.39 (-0.50 to -0.27)^***^ | -0.21 (-0.32 to -0.09)^***^ |
| Only with obesity | 769 | -0.12 (-0.29 to 0.04) | -0.06 (-0.22 to 0.09) | 0.05 (-0.10 to 0.20) |
| Sarcopenic obesity | 1,360 | -0.85 (-0.99 to -0.72)^***^ | -0.52 (-0.65 to -0.38)^***^ | -0.24 (-0.38 to -0.11)^***^ |
| P for interaction |  | 0.67 | 0.52 | 0.41 |
| Sarcopenic obesity (ABSI) |  |  |  |  |
| Without sarcopenia and obesity | 1,925 | Ref. (0) | Ref. (0) | Ref. (0) |
| Only with sarcopenia | 2,335 | -0.61 (-0.73 to -0.49)^***^ | -0.39 (-0.51 to -0.27)^***^ | -0.23 (-0.35 to -0.12)^***^ |
| Only with obesity | 701 | -0.32 (-0.48 to -0.15)^***^ | -0.20 (-0.36 to -0.04)^*^ | -0.10 (-0.26 to 0.05) |
| Sarcopenic obesity | 1,429 | -1.09 (-1.22 to -0.96)^***^ | -0.64 (-0.78 to -0.50)^***^ | -0.34 (-0.47 to -0.20)^***^ |
| P for interaction |  | 0.12 | 0.63 | 0.98 |
| Sarcopenic obesity (VAI) |  |  |  |  |
| Without sarcopenia and obesity | 1,806 | Ref. (0) | Ref. (0) | Ref. (0) |
| Only with sarcopenia | 2,454 | -0.67 (-0.80 to -0.56)^***^ | -0.38 (-0.50 to -0.26)^***^ | -0.21 (-0.32 to -0.09)^***^ |
| Only with obesity | 820 | -0.04 (-0.20 to 0.12) | 0.01 (-0.15 to 0.16) | 0.08 (-0.07 to 0.23) |
| Sarcopenic obesity | 1,310 | -0.80 (-0.93 to -0.66)^***^ | -0.47 (-0.61 to -0.33)^***^ | -0.21 (-0.34 to -0.07)^**^ |
| P for interaction |  | 0.46 | 0.32 | 0.38 |
| Sarcopenic obesity (CVAI) |  |  |  |  |
| Without sarcopenia and obesity | 1,964 | Ref. (0) | Ref. (0) | Ref. (0) |
| Only with sarcopenia | 2,296 | -0.65 (-0.77 to -0.53)^***^ | -0.40 (-0.51 to -0.28)^***^ | -0.22 (-0.34 to -0.11)^***^ |
| Only with obesity | 662 | -0.22 (-0.39 to -0.05)^*^ | -0.03 (-0.20 to 0.14) | 0.07 (-0.09 to 0.24) |
| Sarcopenic obesity | 1,468 | -0.94 (-1.07 to -0.81)^***^ | -0.47 (-0.61 to -0.33)^***^ | -0.20 (-0.33 to -0.06)^**^ |
| P for interaction |  | 0.50 | 0.72 | 0.63 |
| Sarcopenic obesity (BRI) |  |  |  |  |
| Without sarcopenia and obesity | 1,951 | Ref. (0) | Ref. (0) | Ref. (0) |
| Only with sarcopenia | 2,310 | -0.66 (-0.78 to -0.55)^***^ | -0.41 (-0.52 to -0.29)^***^ | -0.23 (-0.35 to -0.12)^***^ |
| Only with obesity | 675 | -0.30 (-0.47 to -0.14)^***^ | -0.19 (-0.36 to -0.02)^*^ | -0.06 (-0.22 to 0.10) |
| Sarcopenic obesity | 1,454 | -0.98 (-1.11 to -0.85)^***^ | -0.57 (-0.71 to -0.43)^***^ | -0.29 (-0.42 to -0.15)^***^ |
| P for interaction |  | 0.91 | 0.80 | 0.92 |
| Sarcopenic obesity (Conicity index) |  |  |  |  |
| Without sarcopenia and obesity | 1,951 | Ref. (0) | Ref. (0) | Ref. (0) |
| Only with sarcopenia | 2,309 | -0.57 (-0.68 to -0.45)^***^ | -0.37 (-0.49 to -0.25)^***^ | -0.23 (-0.35 to -0.12)^***^ |
| Only with obesity | 675 | -0.34 (-0.51 to -0.17)^***^ | -0.20 (-0.37 to -0.03)^*^ | -0.13 (-0.29 to 0.03) |
| Sarcopenic obesity | 1,455 | -1.16 (-1.29 to -1.03)^***^ | -0.69 (-0.83 to -0.55)^***^ | -0.37 (-0.51 to -0.23)^***^ |
| P for interaction |  | 0.02 | 0.26 | 0.97 |
| Sarcopenic obesity (BAI) |  |  |  |  |
| Without sarcopenia and obesity | 1,926 | Ref. (0) | Ref. (0) | Ref. (0) |
| Only with sarcopenia | 2,335 | -0.69 (-0.81 to -0.58)^***^ | -0.42 (-0.54 to -0.30)^***^ | -0.24 (-0.35 to -0.13)^***^ |
| Only with obesity | 700 | -0.18 (-0.35 to -0.01)^*^ | -0.11 (-0.27 to 0.06) | 0.002 (-0.16 to 0.16) |
| Sarcopenic obesity | 1,429 | -0.86 (-0.99 to -0.72)^***^ | -0.48 (-0.61 to -0.34)^***^ | -0.22 (-0.36 to -0.09)^**^ |
| P for interaction |  | 0.88 | 0.62 | 0.89 |
| Sarcopenic obesity (CMI) |  |  |  |  |
| Without sarcopenia and obesity | 1,840 | Ref. (0) | Ref. (0) | Ref. (0) |
| Only with sarcopenia | 2,420 | -0.68 (-0.80 to -0.56)^***^ | -0.38 (-0.50 to -0.26)^***^ | -0.21 (-0.32 to -0.09)^***^ |
| Only with obesity | 786 | -0.07 (-0.23 to 0.10) | -0.01 (-0.17 to 0.14) | 0.08 (-0.07 to 0.23) |
| Sarcopenic obesity | 1,344 | -0.81 (-0.95 to -0.68)^***^ | -0.49 (-0.63 to -0.35)^***^ | -0.22 (-0.35 to -0.08)^**^ |
| P for interaction |  | 0.51 | 0.35 | 0.35 |
| Sarcopenic obesity (BSA) |  |  |  |  |
| Without sarcopenia and obesity | 1,686 | Ref. (0) | Ref. (0) | Ref. (0) |
| Only with sarcopenia | 2,574 | -0.78 (-0.90 to -0.66)^***^ | -0.46 (-0.59 to -0.34)^***^ | -0.27 (-0.39 to -0.15)^***^ |
| Only with obesity | 940 | 0.08 (-0.08 to 0.23) | 0.02 (-0.13 to 0.17) | 0.02 (-0.13 to 0.16) |
| Sarcopenic obesity | 1,190 | -0.46 (-0.60 to -0.31)^***^ | -0.30 (-0.44 to -0.16)^***^ | -0.15 (-0.29 to -0.01)^*^ |
| P for interaction |  | 0.02 | 0.14 | 0.30 |
| Sarcopenic obesity (WHHR) |  |  |  |  |
| Without sarcopenia and obesity | 2,018 | Ref. (0) | Ref. (0) | Ref. (0) |
| Only with sarcopenia | 2,242 | -0.54 (-0.66 to -0.43)^***^ | -0.33 (-0.45 to -0.22)^***^ | -0.18 (-0.29 to -0.07)^**^ |
| Only with obesity | 608 | -0.29 (-0.47 to -0.12)^**^ | -0.15 (-0.32 to 0.02) | -0.01 (-0.18 to 0.15) |
| Sarcopenic obesity | 1,522 | -1.12 (-1.25 to -0.99)^***^ | -0.68 (-0.81 to -0.54)^***^ | -0.36 (-0.50 to -0.22)^***^ |
| P for interaction |  | 0.01 | 0.07 | 0.10 |
| Sarcopenic obesity (PFM) |  |  |  |  |
| Without sarcopenia and obesity | 1,835 | Ref. (0) | Ref. (0) | Ref. (0) |
| Only with sarcopenia | 2,425 | -0.72 (-0.84 to -0.61)^***^ | -0.41 (-0.53 to -0.29)^***^ | -0.22 (-0.34 to -0.10)^***^ |
| Only with obesity | 791 | -0.05 (-0.21 to 0.12) | -0.02 (-0.18 to 0.13) | 0.03 (-0.12 to 0.18) |
| Sarcopenic obesity | 1,339 | -0.72 (-0.86 to -0.58)^***^ | -0.44 (-0.58 to -0.30)^***^ | -0.24 (-0.37 to -0.11)^***^ |
| P for interaction |  | 0.63 | 0.94 | 0.62 |
| Sarcopenic obesity (PLM) |  |  |  |  |
| Without sarcopenia and obesity | 1,658 | Ref. (0) | Ref. (0) | Ref. (0) |
| Only with sarcopenia | 2,602 | -0.80 (-0.92 to -0.68)^***^ | -0.48 (-0.60 to -0.35)^***^ | -0.28 (-0.40 to -0.16)^***^ |
| Only with obesity | 968 | 0.08 (-0.07 to 0.24) | -0.0002 (-0.15 to 0.15) | 0.0005 (-0.14 to 0.14) |
| Sarcopenic obesity | 1,162 | -0.40 (-0.55 to -0.26)^***^ | -0.30 (-0.44 to -0.15)^***^ | -0.15 (-0.29 to -0.02)^*^ |
| P for interaction |  | 0.002 | 0.07 | 0.20 |
| Sarcopenic obesity (PPF) |  |  |  |  |
| Without sarcopenia and obesity | 1,966 | Ref. (0) | Ref. (0) | Ref. (0) |
| Only with sarcopenia | 2,294 | -0.63 (-0.75 to -0.52)^***^ | -0.38 (-0.49 to -0.26)^***^ | -0.20 (-0.32 to -0.09)^**^ |
| Only with obesity | 660 | -0.19 (-0.36 to -0.02)^*^ | -0.08 (-0.24 to 0.09) | -0.004 (-0.16 to 0.16) |
| Sarcopenic obesity | 1,470 | -0.95 (-1.08 to -0.82)^***^ | -0.54 (-0.68 to -0.40)^***^ | -0.30 (-0.43 to -0.17)^***^ |
| P for interaction |  | 0.26 | 0.42 | 0.35 |
| Sarcopenic obesity (CUN-BAE) |  |  |  |  |
| Without sarcopenia and obesity | 1,959 | Ref. (0) | Ref. (0) | Ref. (0) |
| Only with sarcopenia | 2,301 | -0.65 (-0.77 to -0.54)^***^ | -0.39 (-0.51 to -0.27)^***^ | -0.20 (-0.32 to -0.09)^**^ |
| Only with obesity | 667 | -0.15 (-0.33 to 0.02) | -0.01 (-0.18 to 0.15) | 0.05 (-0.11 to 0.21) |
| Sarcopenic obesity | 1,463 | -0.89 (-1.03 to -0.76)^***^ | -0.47 (-0.60 to -0.33)^***^ | -0.25 (-0.39 to -0.12)^***^ |
| P for interaction |  | 0.43 | 0.54 | 0.30 |

ABSI: a body shape index; BAI: body adiposity index; BMI: body mass index; BRI: body roundness index; BSA: body surface area; CI: confidence interval; CMI: cardiometabolic index; CUN-BAE: Clínica Universidad de Navarra-Body Adiposity Estimator; CVAI: Chinese visceral adiposity index; DWRT: Delayed Word Recall Test; HC, hip circumference; LAP: lipid accumulation product; PFM: predicted fat mass; PLM: predicted lean mass; PPF: predicated percent fat; Ref, reference; VAI: visceral adiposity index; WC: waist circumference; WHHR: waist-to-hip-to-height ratio; WHR: waist-to-hip ratio; WHtR: waist-to-height ratio

^a^: Unadjusted

^b^: Adjusted for sex and age

^c^: Additionally adjusted for education, occupation, personal income, physical activity, drinking, smoking and self-rated health

^*^P <0.05, ^**^P <0.01, ^***^P <0.001

Table S5. Associations of baseline sarcopenia and sarcopenic obesity with memory function at follow-up.

|  | *N* | Adjusted mean differences β (95% CI) in follow-up DWRT score | | |
| --- | --- | --- | --- | --- |
|  |  | Model 1^a^ | Model 2^b^ | Model 3^c^ |
| Sarcopenia |  |  |  |  |
| No | 1,800 | Ref. (0) | Ref. (0) | Ref. (0) |
| Yes | 2,179 | -0.27 (-0.37 to -0.16)^***^ | -0.17 (-0.28 to -0.07)^**^ | -0.10 (-0.20 to -0.004)^*^ |
| Sarcopenic obesity (Weight) |  |  |  |  |
| Without sarcopenia and obesity | 1,198 | Ref. (0) | Ref. (0) | Ref. (0) |
| Only with sarcopenia | 1,415 | -0.33 (-0.46 to -0.20)^***^ | -0.24 (-0.36 to -0.11)^***^ | -0.17 (-0.29 to -0.05)^**^ |
| Only with obesity | 602 | -0.08 (-0.23 to 0.08) | -0.07 (-0.22 to 0.09) | -0.06 (-0.21 to 0.08) |
| Sarcopenic obesity | 764 | -0.23 (-0.38 to -0.08)^**^ | -0.13 (-0.27 to 0.02) | -0.05 (-0.19 to 0.09) |
| P for interaction |  | 0.11 | 0.10 | 0.07 |
| Sarcopenic obesity (BMI) |  |  |  |  |
| Without sarcopenia and obesity | 1,249 | Ref. (0) | Ref. (0) | Ref. (0) |
| Only with sarcopenia | 1,339 | -0.29 (-0.42 to -0.16)^***^ | -0.20 (-0.33 to -0.07)^**^ | -0.13 (-0.25 to -0.01)^*^ |
| Only with obesity | 551 | -0.08 (-0.24 to 0.08) | -0.04 (-0.20 to 0.12) | -0.03 (-0.18 to 0.13) |
| Sarcopenic obesity | 840 | -0.30 (-0.44 to -0.15)^***^ | -0.16 (-0.31 to -0.02)^*^ | -0.08 (-0.22 to 0.05) |
| P for interaction |  | 0.52 | 0.44 | 0.47 |
| Sarcopenic obesity (WC) |  |  |  |  |
| Without sarcopenia and obesity | 1,374 | Ref. (0) | Ref. (0) | Ref. (0) |
| Only with sarcopenia | 1,471 | -0.25 (-0.38 to -0.13)^***^ | -0.17 (-0.29 to -0.05)^*^ | -0.10 (-0.22 to 0.01) |
| Only with obesity | 426 | -0.25 (-0.43 to -0.07)^**^ | -0.17 (-0.34 to 0.003) | -0.18 (-0.34 to -0.01)^*^ |
| Sarcopenic obesity | 708 | -0.49 (-0.65 to -0.34)^***^ | -0.32 (-0.47 to -0.16)^***^ | -0.25 (-0.40 to -0.10)^**^ |
| P for interaction |  | 0.94 | 0.81 | 0.78 |
| Sarcopenic obesity (HC) |  |  |  |  |
| Without sarcopenia and obesity | 1,274 | Ref. (0) | Ref. (0) | Ref. (0) |
| Only with sarcopenia | 1,491 | -0.32 (-0.44 to -0.19)^***^ | -0.22 (-0.34 to -0.10)^***^ | -0.15 (-0.26 to -0.03)^*^ |
| Only with obesity | 526 | -0.06 (-0.23 to 0.10) | -0.04 (-0.20 to 0.12) | -0.03 (-0.18 to 0.13) |
| Sarcopenic obesity | 688 | -0.22 (-0.37 to -0.06)^**^ | -0.11 (-0.26 to 0.04) | -0.04 (-0.18 to 0.11) |
| P for interaction |  | 0.14 | 0.16 | 0.19 |
| Sarcopenic obesity (WHR) |  |  |  |  |
| Without sarcopenia and obesity | 658 | Ref. (0) | Ref. (0) | Ref. (0) |
| Only with sarcopenia | 587 | -0.24 (-0.42 to -0.06)^*^ | -0.19 (-0.37 to -0.02)^*^ | -0.15 (-0.31 to 0.02) |
| Only with obesity | 1,142 | -0.19 (-0.35 to -0.04)^*^ | -0.16 (-0.31 to -0.005)^*^ | -0.13 (-0.27 to 0.02) |
| Sarcopenic obesity | 1,592 | -0.46 (-0.62 to -0.30)^***^ | -0.31 (-0.47 to -0.16)^***^ | -0.21 (-0.35 to -0.06)^**^ |
| P for interaction |  | 0.79 | 0.71 | 0.50 |
| Sarcopenic obesity (WHtR) |  |  |  |  |
| Without sarcopenia and obesity | 1,147 | Ref. (0) | Ref. (0) | Ref. (0) |
| Only with sarcopenia | 1,081 | -0.23 (-0.36 to -0.09)^**^ | -0.15 (-0.29 to -0.02)^*^ | -0.09 (-0.22 to 0.04) |
| Only with obesity | 653 | -0.26 (-0.42 to -0.10)^**^ | -0.18 (-0.34 to -0.03)^*^ | -0.16 (-0.30 to -0.01)^*^ |
| Sarcopenic obesity | 1,098 | -0.52 (-0.66 to -0.38)^***^ | -0.35 (-0.49 to -0.21)^***^ | -0.25 (-0.39 to -0.12)^***^ |
| P for interaction |  | 0.74 | 0.89 | 0.92 |
| Sarcopenic obesity (LAP) |  |  |  |  |
| Without sarcopenia and obesity | 1,279 | Ref. (0) | Ref. (0) | Ref. (0) |
| Only with sarcopenia | 1,411 | -0.27 (-0.40 to -0.15)^***^ | -0.19 (-0.31 to -0.07)^**^ | -0.12 (-0.24 to -0.01)^*^ |
| Only with obesity | 521 | -0.25 (-0.41 to -0.08)^**^ | -0.19 (-0.35 to -0.03)^*^ | -0.19 (-0.35 to -0.04)^*^ |
| Sarcopenic obesity | 768 | -0.46 (-0.61 to -0.31)^***^ | -0.31 (-0.45 to -0.16)^***^ | -0.23 (-0.38 to -0.09)^**^ |
| P for interaction |  | 0.58 | 0.51 | 0.41 |
| Sarcopenic obesity (ABSI) |  |  |  |  |
| Without sarcopenia and obesity | 1,351 | Ref. (0) | Ref. (0) | Ref. (0) |
| Only with sarcopenia | 1,426 | -0.20 (-0.32 to -0.07)^**^ | -0.12 (-0.24 to 0.0003) | -0.05 (-0.16 to 0.06) |
| Only with obesity | 449 | -0.08 (-0.26 to 0.09) | -0.02 (-0.19 to 0.15) | 0.01 (-0.16 to 0.17) |
| Sarcopenic obesity | 753 | -0.50 (-0.66 to -0.35)^***^ | -0.32 (-0.48 to -0.17)^***^ | -0.23 (-0.38 to -0.09)^**^ |
| P for interaction |  | 0.05 | 0.11 | 0.08 |
| Sarcopenic obesity (VAI) |  |  |  |  |
| Without sarcopenia and obesity | 1,241 | Ref. (0) | Ref. (0) | Ref. (0) |
| Only with sarcopenia | 1,434 | -0.27 (-0.40 to -0.15)^***^ | -0.19 (-0.31 to -0.06)^**^ | -0.11 (-0.23 to 0.01) |
| Only with obesity | 559 | -0.19 (-0.35 to -0.03)^*^ | -0.14 (-0.30 to 0.02) | -0.15 (-0.30 to -0.002)^*^ |
| Sarcopenic obesity | 745 | -0.43 (-0.58 to -0.28)^***^ | -0.28 (-0.43 to -0.13)^***^ | -0.23 (-0.37 to -0.09)^**^ |
| P for interaction |  | 0.74 | 0.69 | 0.74 |
| Sarcopenic obesity (CVAI) |  |  |  |  |
| Without sarcopenia and obesity | 1,360 | Ref. (0) | Ref. (0) | Ref. (0) |
| Only with sarcopenia | 1,358 | -0.23 (-0.36 to -0.11)^***^ | -0.14 (-0.26 to -0.02)^*^ | -0.07 (-0.18 to 0.05) |
| Only with obesity | 440 | -0.14 (-0.31 to 0.04) | -0.07 (-0.24 to 0.11) | -0.07 (-0.23 to 0.10) |
| Sarcopenic obesity | 821 | -0.45 (-0.60 to -0.30)^***^ | -0.30 (-0.45 to -0.15)^***^ | -0.24 (-0.38 to -0.10)^**^ |
| P for interaction |  | 0.47 | 0.41 | 0.31 |
| Sarcopenic obesity (BRI) |  |  |  |  |
| Without sarcopenia and obesity | 1,355 | Ref. (0) | Ref. (0) | Ref. (0) |
| Only with sarcopenia | 1,356 | -0.24 (-0.37 to -0.12)^***^ | -0.16 (-0.28 to -0.04)^*^ | -0.10 (-0.21 to 0.02) |
| Only with obesity | 445 | -0.27 (-0.44 to -0.09)^**^ | -0.19 (-0.36 to -0.02)^*^ | -0.18 (-0.35 to -0.02)^*^ |
| Sarcopenic obesity | 823 | -0.52 (-0.67 to -0.37)^***^ | -0.35 (-0.49 to -0.20)^***^ | -0.27 (-0.41 to -0.12)^***^ |
| P for interaction |  | 0.93 | 0.96 | 0.88 |
| Sarcopenic obesity (Conicity index) |  |  |  |  |
| Without sarcopenia and obesity | 1,364 | Ref. (0) | Ref. (0) | Ref. (0) |
| Only with sarcopenia | 1,443 | -0.19 (-0.31 to -0.07)^**^ | -0.11 (-0.23 to 0.01) | -0.03 (-0.15 to 0.08) |
| Only with obesity | 436 | 0.09 (-0.08 to 0.27) | 0.13 (-0.04 to 0.31) | 0.18 (0.01 to 0.34)^*^ |
| Sarcopenic obesity | 736 | -0.37 (-0.53 to -0.21)^***^ | -0.20 (-0.36 to -0.04)^*^ | -0.12 (-0.27 to 0.02) |
| P for interaction |  | 0.02 | 0.05 | 0.01 |
| Sarcopenic obesity (BAI) |  |  |  |  |
| Without sarcopenia and obesity | 1,318 | Ref. (0) | Ref. (0) | Ref. (0) |
| Only with sarcopenia | 1,354 | -0.25 (-0.38 to -0.12)^***^ | -0.16 (-0.28 to -0.03)^*^ | -0.09 (-0.21 to 0.03) |
| Only with obesity | 482 | -0.12 (-0.28 to 0.05) | -0.04 (-0.20 to 0.13) | -0.04 (-0.19 to 0.12) |
| Sarcopenic obesity | 825 | -0.39 (-0.53 to -0.24)^***^ | -0.23 (-0.38 to -0.08)^**^ | -0.16 (-0.30 to -0.02)^*^ |
| P for interaction |  | 0.85 | 0.75 | 0.77 |
| Sarcopenic obesity (CMI) |  |  |  |  |
| Without sarcopenia and obesity | 1,263 | Ref. (0) | Ref. (0) | Ref. (0) |
| Only with sarcopenia | 1,410 | -0.27 (-0.40 to -0.14)^***^ | -0.18 (-0.31 to -0.06)^**^ | -0.11 (-0.23 to 0.01) |
| Only with obesity | 537 | -0.24 (-0.40 to -0.08)^**^ | -0.17 (-0.33 to -0.01)^*^ | -0.19 (-0.34 to -0.04)^*^ |
| Sarcopenic obesity | 769 | -0.47 (-0.62 to -0.32)^***^ | -0.31 (-0.46 to -0.16)^***^ | -0.26 (-0.40 to -0.12)^***^ |
| P for interaction |  | 0.70 | 0.73 | 0.72 |
| Sarcopenic obesity (BSA) |  |  |  |  |
| Without sarcopenia and obesity | 1,150 | Ref. (0) | Ref. (0) | Ref. (0) |
| Only with sarcopenia | 1,462 | -0.34 (-0.47 to -0.21)^***^ | -0.25 (-0.38 to -0.12)^***^ | -0.18 (-0.30 to -0.06)^**^ |
| Only with obesity | 650 | -0.01 (-0.17 to 0.15) | -0.01 (-0.17 to 0.14) | -0.02 (-0.17 to 0.12) |
| Sarcopenic obesity | 717 | -0.14 (-0.29 to 0.01) | -0.05 (-0.20 to 0.10) | 0.01 (-0.13 to 0.15) |
| P for interaction |  | 0.05 | 0.05 | 0.04 |
| Sarcopenic obesity (WHHR) |  |  |  |  |
| Without sarcopenia and obesity | 1,404 | Ref. (0) | Ref. (0) | Ref. (0) |
| Only with sarcopenia | 1,348 | -0.21 (-0.33 to -0.08)^**^ | -0.13 (-0.25 to -0.01)^*^ | -0.07 (-0.19 to 0.04) |
| Only with obesity | 396 | -0.27 (-0.45 to -0.09)^**^ | -0.17 (-0.35 to 0.01) | -0.17 (-0.34 to -0.01)^*^ |
| Sarcopenic obesity | 831 | -0.58 (-0.73 to -0.43)^***^ | -0.39 (-0.54 to -0.24)^***^ | -0.31 (-0.45 to -0.17)^***^ |
| P for interaction |  | 0.36 | 0.42 | 0.56 |
| Sarcopenic obesity (PFM) |  |  |  |  |
| Without sarcopenia and obesity | 1,241 | Ref. (0) | Ref. (0) | Ref. (0) |
| Only with sarcopenia | 1,385 | -0.29 (-0.42 to -0.16)^***^ | -0.20 (-0.33 to -0.08)^**^ | -0.14 (-0.26 to -0.02)^*^ |
| Only with obesity | 559 | -0.10 (-0.26 to 0.06) | -0.08 (-0.24 to 0.08) | -0.07 (-0.22 to 0.07) |
| Sarcopenic obesity | 794 | -0.30 (-0.45 to -0.16)^***^ | -0.19 (-0.33 to -0.04)^*^ | -0.11 (-0.25 to 0.03) |
| P for interaction |  | 0.42 | 0.38 | 0.31 |
| Sarcopenic obesity (PLM) |  |  |  |  |
| Without sarcopenia and obesity | 1,138 | Ref. (0) | Ref. (0) | Ref. (0) |
| Only with sarcopenia | 1,458 | -0.35 (-0.48 to -0.22)^***^ | -0.25 (-0.38 to -0.13)^***^ | -0.18 (-0.30 to -0.06)^**^ |
| Only with obesity | 662 | -0.07 (-0.22 to 0.09) | -0.07 (-0.22 to 0.08) | -0.07 (-0.22 to 0.07) |
| Sarcopenic obesity | 721 | -0.19 (-0.34 to -0.04)^*^ | -0.10 (-0.25 to 0.05) | -0.04 (-0.18 to 0.10) |
| P for interaction |  | 0.03 | 0.03 | 0.03 |
| Sarcopenic obesity (PPF) |  |  |  |  |
| Without sarcopenia and obesity | 1,348 | Ref. (0) | Ref. (0) | Ref. (0) |
| Only with sarcopenia | 1,341 | -0.26 (-0.38 to -0.13)^***^ | -0.18 (-0.30 to -0.05)^**^ | -0.12 (-0.23 to 0.001) |
| Only with obesity | 452 | -0.18 (-0.35 to -0.01)^*^ | -0.14 (-0.31 to 0.03) | -0.12 (-0.28 to 0.04) |
| Sarcopenic obesity | 838 | -0.41 (-0.56 to -0.27)^***^ | -0.27 (-0.42 to -0.12)^***^ | -0.18 (-0.31 to -0.04)^*^ |
| P for interaction |  | 0.83 | 0.70 | 0.55 |
| Sarcopenic obesity (CUN-BAE) |  |  |  |  |
| Without sarcopenia and obesity | 1,339 | Ref. (0) | Ref. (0) | Ref. (0) |
| Only with sarcopenia | 1,317 | -0.27 (-0.39 to -0.14)^***^ | -0.17 (-0.30 to -0.05)^**^ | -0.12 (-0.24 to -0.002)^*^ |
| Only with obesity | 461 | -0.15 (-0.32 to 0.02) | -0.10 (-0.27 to 0.07) | -0.12 (-0.27 to 0.04) |
| Sarcopenic obesity | 862 | -0.38 (-0.53 to -0.23)^***^ | -0.25 (-0.39 to -0.10)^**^ | -0.16 (-0.30 to -0.02)^*^ |
| P for interaction |  | 0.75 | 0.81 | 0.49 |

ABSI: a body shape index; BAI: body adiposity index; BMI: body mass index; BRI: body roundness index; BSA: body surface area; CI: confidence interval; CMI: cardiometabolic index; CUN-BAE: Clínica Universidad de Navarra-Body Adiposity Estimator; CVAI: Chinese visceral adiposity index; DWRT: Delayed Word Recall Test; HC, hip circumference; LAP: lipid accumulation product; PFM: predicted fat mass; PLM: predicted lean mass; PPF: predicated percent fat; Ref, reference; VAI: visceral adiposity index; WC: waist circumference; WHHR: waist-to-hip-to-height ratio; WHR: waist-to-hip ratio; WHtR: waist-to-height ratio

^a^: Adjusted for sex and age

^b^: Additionally adjusted for education, occupation, personal income, physical activity, drinking, smoking and self-rated health

^c^: Additionally adjusted for baseline DWRT score

^*^P <0.05, ^**^P <0.01, ^***^P <0.001

Table S6. Associations of sarcopenia and sarcopenic obesity with memory function using generalised estimating equation.

|  | Adjusted mean differences β (95% CI) in DWRT score | | |
| --- | --- | --- | --- |
|  | Model 1^a^ | Model 2^b^ | Model 3^c^ |
| Sarcopenia |  |  |  |
| No | Ref. (0) | Ref. (0) | Ref. (0) |
| Yes | -0.36 (-0.42 to -0.29)^***^ | -0.26 (-0.32 to -0.20)^***^ | -0.13 (-0.19 to -0.07)^***^ |
| Sarcopenic obesity (Weight) |  |  |  |
| Without sarcopenia and obesity | Ref. (0) | Ref. (0) | Ref. (0) |
| Only with sarcopenia | -0.39 (-0.46 to -0.31)^***^ | -0.27 (-0.34 to -0.19)^***^ | -0.13 (-0.21 to -0.06)^***^ |
| Only with obesity | 0.02 (-0.07 to 0.10) | -0.01 (-0.10 to 0.07) | 0.01 (-0.07 to 0.10) |
| Sarcopenic obesity | -0.26 (-0.36 to -0.16)^***^ | -0.26 (-0.35 to -0.16)^***^ | -0.12 (-0.21 to -0.02)^*^ |
| P for interaction | 0.09 | 0.69 | 0.97 |
| Sarcopenic obesity (BMI) |  |  |  |
| Without sarcopenia and obesity | Ref. (0) | Ref. (0) | Ref. (0) |
| Only with sarcopenia | -0.38 (-0.46 to -0.31)^***^ | -0.26 (-0.34 to -0.18)^***^ | -0.13 (-0.20 to -0.05)^**^ |
| Only with obesity | -0.10 (-0.19 to -0.02)^*^ | -0.09 (-0.17 to -0.002)^*^ | -0.02 (-0.11 to 0.06) |
| Sarcopenic obesity | -0.40 (-0.50 to -0.31)^***^ | -0.34 (-0.43 to -0.24)^***^ | -0.17 (-0.26 to -0.08)^***^ |
| P for interaction | 0.22 | 0.84 | 0.76 |
| Sarcopenic obesity (WC) |  |  |  |
| Without sarcopenia and obesity | Ref. (0) | Ref. (0) | Ref. (0) |
| Only with sarcopenia | -0.41 (-0.49 to -0.32)^***^ | -0.30 (-0.38 to -0.22)^***^ | -0.15 (-0.23 to -0.08)^***^ |
| Only with obesity | -0.20 (-0.28 to -0.12)^***^ | -0.15 (-0.23 to -0.07)^***^ | -0.08 (-0.16 to 0.004) |
| Sarcopenic obesity | -0.50 (-0.59 to -0.41)^***^ | -0.37 (-0.47 to -0.28)^***^ | -0.19 (-0.29 to -0.10)^***^ |
| P for interaction | 0.11 | 0.23 | 0.52 |
| Sarcopenic obesity (HC) |  |  |  |
| Without sarcopenia and obesity | Ref. (0) | Ref. (0) | Ref. (0) |
| Only with sarcopenia | -0.41 (-0.49 to -0.33)^***^ | -0.28 (-0.36 to -0.20)^***^ | -0.14 (-0.22 to -0.06)^**^ |
| Only with obesity | -0.10 (-0.18 to -0.01)^*^ | -0.04 (-0.12 to 0.05) | -0.03 (-0.12 to 0.05) |
| Sarcopenic obesity | -0.39 (-0.49 to -0.30)^***^ | -0.28 (-0.37 to -0.18)^***^ | -0.18 (-0.27 to -0.09)^***^ |
| P for interaction | 0.08 | 0.59 | 0.90 |
| Sarcopenic obesity (WHR) |  |  |  |
| Without sarcopenia and obesity | Ref. (0) | Ref. (0) | Ref. (0) |
| Only with sarcopenia | -0.38 (-0.51 to -0.25)^***^ | -0.27 (-0.39 to -0.15)^***^ | -0.13 (-0.25 to -0.01)^*^ |
| Only with obesity | -0.10 (-0.20 to -0.01)^*^ | -0.09 (-0.18 to 0.01) | -0.03 (-0.13 to 0.07) |
| Sarcopenic obesity | -0.45 (-0.55 to -0.35)^***^ | -0.35 (-0.45 to -0.25)^***^ | -0.17 (-0.27 to -0.07)^**^ |
| P for interaction | 0.71 | 0.88 | 0.91 |
| Sarcopenic obesity (WHtR) |  |  |  |
| Without sarcopenia and obesity | Ref. (0) | Ref. (0) | Ref. (0) |
| Only with sarcopenia | -0.39 (-0.49 to -0.30)^***^ | -0.28 (-0.37 to -0.19)^***^ | -0.13 (-0.22 to -0.04)^**^ |
| Only with obesity | -0.30 (-0.38 to -0.22)^***^ | -0.14 (-0.22 to -0.06)^**^ | -0.08 (-0.16 to 0.01) |
| Sarcopenic obesity | -0.64 (-0.72 to -0.55)^***^ | -0.39 (-0.48 to -0.31)^***^ | -0.22 (-0.31 to -0.13)^***^ |
| P for interaction | 0.38 | 0.67 | 0.91 |
| Sarcopenic obesity (LAP) |  |  |  |
| Without sarcopenia and obesity | Ref. (0) | Ref. (0) | Ref. (0) |
| Only with sarcopenia | -0.37 (-0.45 to -0.29)^***^ | -0.26 (-0.34 to -0.18)^***^ | -0.13 (-0.21 to -0.06)^**^ |
| Only with obesity | -0.20 (-0.29 to -0.11)^***^ | -0.12 (-0.20 to -0.04)^**^ | -0.05 (-0.14 to 0.03) |
| Sarcopenic obesity | -0.56 (-0.65 to -0.46)^***^ | -0.41 (-0.50 to -0.31)^***^ | -0.20 (-0.30 to -0.11)^***^ |
| P for interaction | 0.81 | 0.68 | 0.80 |
| Sarcopenic obesity (ABSI) |  |  |  |
| Without sarcopenia and obesity | Ref. (0) | Ref. (0) | Ref. (0) |
| Only with sarcopenia | -0.42 (-0.51 to -0.33)^***^ | -0.31 (-0.40 to -0.22)^***^ | -0.17 (-0.26 to -0.08)^***^ |
| Only with obesity | -0.37 (-0.45 to -0.29)^***^ | -0.14 (-0.22 to -0.05)^**^ | -0.12 (-0.21 to -0.04)^**^ |
| Sarcopenic obesity | -0.71 (-0.79 to -0.62)^***^ | -0.37 (-0.46 to -0.28)^***^ | -0.24 (-0.34 to -0.15)^***^ |
| P for interaction | 0.20 | 0.21 | 0.42 |
| Sarcopenic obesity (VAI) |  |  |  |
| Without sarcopenia and obesity | Ref. (0) | Ref. (0) | Ref. (0) |
| Only with sarcopenia | -0.38 (-0.46 to -0.31)^***^ | -0.26 (-0.34 to -0.19)^***^ | -0.14 (-0.22 to -0.07)^***^ |
| Only with obesity | -0.12 (-0.21 to -0.04)^**^ | -0.06 (-0.14 to 0.03) | -0.02 (-0.10 to 0.07) |
| Sarcopenic obesity | -0.46 (-0.55 to -0.37)^***^ | -0.33 (-0.42 to -0.24)^***^ | -0.14 (-0.24 to -0.05)^**^ |
| P for interaction | 0.45 | 0.89 | 0.80 |
| Sarcopenic obesity (CVAI) |  |  |  |
| Without sarcopenia and obesity | Ref. (0) | Ref. (0) | Ref. (0) |
| Only with sarcopenia | -0.36 (-0.44 to -0.28)^***^ | -0.26 (-0.34 to -0.18)^***^ | -0.13 (-0.21 to -0.05)^**^ |
| Only with obesity | -0.32 (-0.40 to -0.24)^***^ | -0.09 (-0.17 to -0.003)^*^ | -0.01 (-0.09 to 0.08) |
| Sarcopenic obesity | -0.68 (-0.77 to -0.59)^***^ | -0.37 (-0.46 to -0.28)^***^ | -0.16 (-0.25 to -0.06)^**^ |
| P for interaction | 0.95 | 0.67 | 0.72 |
| Sarcopenic obesity (BRI) |  |  |  |
| Without sarcopenia and obesity | Ref. (0) | Ref. (0) | Ref. (0) |
| Only with sarcopenia | -0.39 (-0.47 to -0.30)^***^ | -0.28 (-0.36 to -0.20)^***^ | -0.14 (-0.22 to -0.06)^**^ |
| Only with obesity | -0.34 (-0.42 to -0.26)^***^ | -0.18 (-0.26 to -0.10)^***^ | -0.10 (-0.18 to -0.02)^*^ |
| Sarcopenic obesity | -0.68 (-0.76 to -0.59)^***^ | -0.43 (-0.52 to -0.34)^***^ | -0.24 (-0.33 to -0.15)^***^ |
| P for interaction | 0.41 | 0.59 | 0.95 |
| Sarcopenic obesity (Conicity index) |  |  |  |
| Without sarcopenia and obesity | Ref. (0) | Ref. (0) | Ref. (0) |
| Only with sarcopenia | -0.34 (-0.42 to -0.26)^***^ | -0.28 (-0.36 to -0.20)^***^ | -0.16 (-0.24 to -0.07)^***^ |
| Only with obesity | -0.38 (-0.47 to -0.30)^***^ | -0.16 (-0.25 to -0.08)^***^ | -0.14 (-0.22 to -0.06)^**^ |
| Sarcopenic obesity | -0.72 (-0.81 to -0.64)^***^ | -0.40 (-0.49 to -0.31)^***^ | -0.25 (-0.34 to -0.16)^***^ |
| P for interaction | 0.97 | 0.51 | 0.50 |
| Sarcopenic obesity (BAI) |  |  |  |
| Without sarcopenia and obesity | Ref. (0) | Ref. (0) | Ref. (0) |
| Only with sarcopenia | -0.39 (-0.47 to -0.30)^***^ | -0.27 (-0.35 to -0.19)^***^ | -0.13 (-0.21 to -0.05)^**^ |
| Only with obesity | -0.28 (-0.36 to -0.20)^***^ | -0.14 (-0.22 to -0.06)^**^ | -0.08 (-0.16 to 0.002) |
| Sarcopenic obesity | -0.62 (-0.71 to -0.53)^***^ | -0.40 (-0.49 to -0.31)^***^ | -0.23 (-0.32 to -0.15)^***^ |
| P for interaction | 0.45 | 0.86 | 0.63 |
| Sarcopenic obesity (CMI) |  |  |  |
| Without sarcopenia and obesity | Ref. (0) | Ref. (0) | Ref. (0) |
| Only with sarcopenia | -0.37 (-0.45 to -0.29)^***^ | -0.25 (-0.33 to -0.17)^***^ | -0.13 (-0.20 to -0.05)^**^ |
| Only with obesity | -0.12 (-0.20 to -0.04)^**^ | -0.05 (-0.13 to 0.03) | 0.01 (-0.08 to 0.09) |
| Sarcopenic obesity | -0.47 (-0.57 to -0.38)^***^ | -0.34 (-0.43 to -0.25)^***^ | -0.14 (-0.23 to -0.05)^**^ |
| P for interaction | 0.76 | 0.52 | 0.75 |
| Sarcopenic obesity (BSA) |  |  |  |
| Without sarcopenia and obesity | Ref. (0) | Ref. (0) | Ref. (0) |
| Only with sarcopenia | -0.38 (-0.46 to -0.30)^***^ | -0.27 (-0.34 to -0.19)^***^ | -0.14 (-0.21 to -0.06)^***^ |
| Only with obesity | 0.12 (0.03 to 0.20)^**^ | 0.05 (-0.03 to 0.13) | 0.04 (-0.04 to 0.13) |
| Sarcopenic obesity | -0.15 (-0.24 to -0.05)^**^ | -0.18 (-0.27 to -0.08)^***^ | -0.07 (-0.17 to 0.03) |
| P for interaction | 0.08 | 0.51 | 0.69 |
| Sarcopenic obesity (WHHR) |  |  |  |
| Without sarcopenia and obesity | Ref. (0) | Ref. (0) | Ref. (0) |
| Only with sarcopenia | -0.31 (-0.39 to -0.23)^***^ | -0.25 (-0.32 to -0.17)^***^ | -0.13 (-0.21 to -0.05)^**^ |
| Only with obesity | -0.39 (-0.48 to -0.31)^***^ | -0.22 (-0.31 to -0.13)^***^ | -0.10 (-0.19 to -0.01)^*^ |
| Sarcopenic obesity | -0.72 (-0.82 to -0.64)^***^ | -0.47 (-0.56 to -0.38)^***^ | -0.23 (-0.32 to -0.14)^***^ |
| P for interaction | 0.72 | 0.99 | 0.96 |
| Sarcopenic obesity (PFM) |  |  |  |
| Without sarcopenia and obesity | Ref. (0) | Ref. (0) | Ref. (0) |
| Only with sarcopenia | -0.37 (-0.45 to -0.30)^***^ | -0.25 (-0.33 to -0.17)^***^ | -0.11 (-0.19 to -0.04)^**^ |
| Only with obesity | -0.11 (-0.20 to -0.03)^**^ | -0.06 (-0.14 to 0.02) | -0.01 (-0.10 to 0.07) |
| Sarcopenic obesity | -0.44 (-0.54 to -0.35)^***^ | -0.34 (-0.43 to -0.24)^***^ | -0.19 (-0.28 to -0.09)^***^ |
| P for interaction | 0.52 | 0.69 | 0.35 |
| Sarcopenic obesity (PLM) |  |  |  |
| Without sarcopenia and obesity | Ref. (0) | Ref. (0) | Ref. (0) |
| Only with sarcopenia | -0.39 (-0.46 to -0.31)^***^ | -0.28 (-0.35 to -0.21)^***^ | -0.16 (-0.23 to -0.08)^***^ |
| Only with obesity | 0.13 (0.05 to 0.22)^**^ | 0.001 (-0.08 to 0.09) | -0.004 (-0.09 to 0.08) |
| Sarcopenic obesity | -0.09 (-0.19 to 0.01) | -0.19 (-0.29 to -0.09)^***^ | -0.08 (-0.18 to 0.02) |
| P for interaction | 0.01 | 0.20 | 0.20 |
| Sarcopenic obesity (PPF) |  |  |  |
| Without sarcopenia and obesity | Ref. (0) | Ref. (0) | Ref. (0) |
| Only with sarcopenia | -0.33 (-0.41 to -0.25)^***^ | -0.23 (-0.31 to -0.15)^***^ | -0.10 (-0.18 to -0.02)^*^ |
| Only with obesity | -0.27 (-0.36 to -0.19)^***^ | -0.09 (-0.17 to -0.003)^*^ | -0.03 (-0.11 to 0.06) |
| Sarcopenic obesity | -0.66 (-0.75 to -0.57)^***^ | -0.39 (-0.48 to -0.30)^***^ | -0.21 (-0.30 to -0.12)^***^ |
| P for interaction | 0.45 | 0.24 | 0.19 |
| Sarcopenic obesity (CUN-BAE) |  |  |  |
| Without sarcopenia and obesity | Ref. (0) | Ref. (0) | Ref. (0) |
| Only with sarcopenia | -0.33 (-0.40 to -0.25)^***^ | -0.23 (-0.31 to -0.15)^***^ | -0.10 (-0.17 to -0.02)^*^ |
| Only with obesity | -0.22 (-0.31 to -0.14)^***^ | -0.06 (-0.14 to 0.03) | 0.002 (-0.08 to 0.09) |
| Sarcopenic obesity | -0.61 (-0.70 to -0.52)^***^ | -0.36 (-0.45 to -0.27)^***^ | -0.19 (-0.28 to -0.10)^***^ |
| P for interaction | 0.37 | 0.24 | 0.13 |

ABSI: a body shape index; BAI: body adiposity index; BMI: body mass index; BRI: body roundness index; BSA: body surface area; CI: confidence interval; CMI: cardiometabolic index; CUN-BAE: Clínica Universidad de Navarra-Body Adiposity Estimator; CVAI: Chinese visceral adiposity index; DWRT: Delayed Word Recall Test; HC, hip circumference; LAP: lipid accumulation product; PFM: predicted fat mass; PLM: predicted lean mass; PPF: predicated percent fat; Ref, reference; VAI: visceral adiposity index; WC: waist circumference; WHHR: waist-to-hip-to-height ratio; WHR: waist-to-hip ratio; WHtR: waist-to-height ratio

^a^: Unadjusted

^b^: Adjusted for sex and age

^c^: Additionally adjusted for education, occupation, personal income, physical activity, drinking, smoking and self-rated health

^*^P <0.05, ^**^P <0.01, ^***^P <0.001

Table S7. Associations of baseline sarcopenia and sarcopenic obesity with annual change of memory function.

|  | *N* | Adjusted mean differences β (95% CI) in annual change of DWRT score | | |
| --- | --- | --- | --- | --- |
|  |  | Model 1^a^ | Model 2^b^ | Model 3^c^ |
| Sarcopenia |  |  |  |  |
| No | 1,800 | Ref. (0) | Ref. (0) | Ref. (0) |
| Yes | 2,179 | 0.03 (-0.01 to 0.07) | 0.02 (-0.02 to 0.05) | -0.03 (-0.06 to -0.001)^*^ |
| Sarcopenic obesity (Weight) |  |  |  |  |
| Without sarcopenia and obesity | 1,198 | Ref. (0) | Ref. (0) | Ref. (0) |
| Only with sarcopenia | 1,415 | 0.01 (-0.04 to 0.05) | -0.01 (-0.05 to 0.04) | -0.05 (-0.09 to -0.02)^**^ |
| Only with obesity | 602 | -0.03 (-0.08 to 0.03) | -0.02 (-0.08 to 0.03) | -0.03 (-0.07 to 0.02) |
| Sarcopenic obesity | 764 | 0.04 (-0.01 to 0.10) | 0.03 (-0.02 to 0.09) | -0.02 (-0.06 to 0.02) |
| P for interaction |  | 0.13 | 0.10 | 0.04 |
| Sarcopenic obesity (BMI) |  |  |  |  |
| Without sarcopenia and obesity | 1,249 | Ref. (0) | Ref. (0) | Ref. (0) |
| Only with sarcopenia | 1,339 | 0.02 (-0.02 to 0.07) | 0.01 (-0.04 to 0.05) | -0.04 (-0.08 to -0.005)^*^ |
| Only with obesity | 551 | 0.003 (-0.05 to 0.06) | 0.001 (-0.06 to 0.06) | -0.01 (-0.06 to 0.03) |
| Sarcopenic obesity | 840 | 0.04 (-0.01 to 0.10) | 0.03 (-0.02 to 0.08) | -0.02 (-0.06 to 0.02) |
| P for interaction |  | 0.64 | 0.59 | 0.36 |
| Sarcopenic obesity (WC) |  |  |  |  |
| Without sarcopenia and obesity | 1,374 | Ref. (0) | Ref. (0) | Ref. (0) |
| Only with sarcopenia | 1,471 | 0.03 (-0.02 to 0.07) | 0.01 (-0.03 to 0.06) | -0.03 (-0.07 to 0.001) |
| Only with obesity | 426 | -0.06 (-0.12 to 0.01) | -0.06 (-0.12 to 0.003) | -0.06 (-0.10 to -0.01)^*^ |
| Sarcopenic obesity | 708 | -0.01 (-0.06 to 0.05) | -0.03 (-0.08 to 0.03) | -0.07 (-0.12 to -0.03)^**^ |
| P for interaction |  | 0.59 | 0.68 | 0.65 |
| Sarcopenic obesity (HC) |  |  |  |  |
| Without sarcopenia and obesity | 1,274 | Ref. (0) | Ref. (0) | Ref. (0) |
| Only with sarcopenia | 1,491 | 0.02 (-0.02 to 0.07) | 0.01 (-0.04 to 0.05) | -0.04 (-0.08 to -0.01)^*^ |
| Only with obesity | 526 | -0.005 (-0.06 to 0.05) | -0.005 (-0.06 to 0.05) | -0.01 (-0.06 to 0.03) |
| Sarcopenic obesity | 688 | 0.04 (-0.01 to 0.10) | 0.03 (-0.02 to 0.09) | -0.01 (-0.06 to 0.03) |
| P for interaction |  | 0.54 | 0.42 | 0.16 |
| Sarcopenic obesity (WHR) |  |  |  |  |
| Without sarcopenia and obesity | 658 | Ref. (0) | Ref. (0) | Ref. (0) |
| Only with sarcopenia | 587 | -0.001 (-0.07 to 0.06) | -0.01 (-0.07 to 0.06) | -0.04 (-0.09 to 0.01) |
| Only with obesity | 1,142 | -0.01 (-0.07 to 0.05) | -0.01 (-0.07 to 0.04) | -0.03 (-0.08 to 0.01) |
| Sarcopenic obesity | 1,592 | 0.03 (-0.02 to 0.09) | 0.02 (-0.04 to 0.07) | -0.06 (-0.10 to -0.02)^**^ |
| P for interaction |  | 0.26 | 0.38 | 0.62 |
| Sarcopenic obesity (WHtR) |  |  |  |  |
| Without sarcopenia and obesity | 1,147 | Ref. (0) | Ref. (0) | Ref. (0) |
| Only with sarcopenia | 1,081 | 0.03 (-0.02 to 0.08) | 0.02 (-0.03 to 0.07) | -0.03 (-0.06 to 0.01) |
| Only with obesity | 653 | -0.02 (-0.07 to 0.04) | -0.02 (-0.08 to 0.03) | -0.04 (-0.09 to -0.001)^*^ |
| Sarcopenic obesity | 1,098 | 0.02 (-0.03 to 0.07) | -0.005 (-0.06 to 0.05) | -0.07 (-0.11 to -0.03)^***^ |
| P for interaction |  | 0.87 | 0.99 | 0.92 |
| Sarcopenic obesity (LAP) |  |  |  |  |
| Without sarcopenia and obesity | 1,279 | Ref. (0) | Ref. (0) | Ref. (0) |
| Only with sarcopenia | 1,411 | 0.02 (-0.02 to 0.07) | 0.01 (-0.04 to 0.05) | -0.04 (-0.07 to -0.002)^*^ |
| Only with obesity | 521 | -0.06 (-0.12 to 0.00003) | -0.06 (-0.12 to -0.01)^*^ | -0.06 (-0.10 to -0.01)^*^ |
| Sarcopenic obesity | 768 | -0.004 (-0.06 to 0.05) | -0.02 (-0.08 to 0.03) | -0.07 (-0.11 to -0.03)^**^ |
| P for interaction |  | 0.40 | 0.41 | 0.42 |
| Sarcopenic obesity (ABSI) |  |  |  |  |
| Without sarcopenia and obesity | 1,351 | Ref. (0) | Ref. (0) | Ref. (0) |
| Only with sarcopenia | 1,426 | 0.05 (0.002 to 0.09)^*^ | 0.04 (-0.01 to 0.08) | -0.01 (-0.05 to 0.02) |
| Only with obesity | 449 | 0.02 (-0.04 to 0.09) | 0.02 (-0.04 to 0.08) | 0.005 (-0.04 to 0.05) |
| Sarcopenic obesity | 753 | 0.01 (-0.04 to 0.07) | -0.01 (-0.06 to 0.05) | -0.07 (-0.11 to -0.03)^**^ |
| P for interaction |  | 0.16 | 0.10 | 0.046 |
| Sarcopenic obesity (VAI) |  |  |  |  |
| Without sarcopenia and obesity | 1,241 | Ref. (0) | Ref. (0) | Ref. (0) |
| Only with sarcopenia | 1,434 | 0.03 (-0.02 to 0.07) | 0.02 (-0.03 to 0.06) | -0.03 (-0.07 to 0.001) |
| Only with obesity | 559 | -0.05 (-0.11 to 0.01) | -0.06 (-0.11 to 0.001) | -0.05 (-0.09 to -0.002)^*^ |
| Sarcopenic obesity | 745 | -0.02 (-0.07 to 0.04) | -0.03 (-0.09 to 0.02) | -0.07 (-0.11 to -0.03)^**^ |
| P for interaction |  | 0.85 | 0.89 | 0.71 |
| Sarcopenic obesity (CVAI) |  |  |  |  |
| Without sarcopenia and obesity | 1,360 | Ref. (0) | Ref. (0) | Ref. (0) |
| Only with sarcopenia | 1,358 | 0.04 (-0.002 to 0.09) | 0.03 (-0.01 to 0.07) | -0.02 (-0.05 to 0.01) |
| Only with obesity | 440 | -0.02 (-0.08 to 0.04) | -0.02 (-0.08 to 0.04) | -0.02 (-0.07 to 0.03) |
| Sarcopenic obesity | 821 | -0.01 (-0.07 to 0.04) | -0.03 (-0.08 to 0.02) | -0.07 (-0.11 to -0.03)^**^ |
| P for interaction |  | 0.41 | 0.33 | 0.35 |
| Sarcopenic obesity (BRI) |  |  |  |  |
| Without sarcopenia and obesity | 1,355 | Ref. (0) | Ref. (0) | Ref. (0) |
| Only with sarcopenia | 1,356 | 0.03 (-0.02 to 0.07) | 0.02 (-0.03 to 0.06) | -0.03 (-0.06 to 0.01) |
| Only with obesity | 445 | -0.04 (-0.10 to 0.02) | -0.05 (-0.11 to 0.02) | -0.05 (-0.10 to -0.003)^*^ |
| Sarcopenic obesity | 823 | 0.004 (-0.05 to 0.06) | -0.02 (-0.07 to 0.03) | -0.07 (-0.12 to -0.03)^***^ |
| P for interaction |  | 0.64 | 0.77 | 0.89 |
| Sarcopenic obesity (Conicity index) |  |  |  |  |
| Without sarcopenia and obesity | 1,364 | Ref. (0) | Ref. (0) | Ref. (0) |
| Only with sarcopenia | 1,443 | 0.06 (0.01 to 0.10)^*^ | 0.05 (0.01 to 0.09)^*^ | -0.01 (-0.04 to 0.03) |
| Only with obesity | 436 | 0.09 (0.03 to 0.15)^**^ | 0.08 (0.02 to 0.15)^**^ | 0.05 (0.01 to 0.10)^*^ |
| Sarcopenic obesity | 736 | 0.04 (-0.01 to 0.10) | 0.01 (-0.04 to 0.07) | -0.04 (-0.08 to 0.01) |
| P for interaction |  | 0.01 | 0.004 | 0.01 |
| Sarcopenic obesity (BAI) |  |  |  |  |
| Without sarcopenia and obesity | 1,318 | Ref. (0) | Ref. (0) | Ref. (0) |
| Only with sarcopenia | 1,354 | 0.03 (-0.01 to 0.08) | 0.02 (-0.02 to 0.06) | -0.03 (-0.06 to 0.01) |
| Only with obesity | 482 | -0.001 (-0.06 to 0.06) | -0.01 (-0.07 to 0.05) | -0.01 (-0.06 to 0.04) |
| Sarcopenic obesity | 825 | 0.02 (-0.03 to 0.08) | 0.004 (-0.05 to 0.06) | -0.05 (-0.09 to -0.004)^*^ |
| P for interaction |  | 0.83 | 0.87 | 0.76 |
| Sarcopenic obesity (CMI) |  |  |  |  |
| Without sarcopenia and obesity | 1,263 | Ref. (0) | Ref. (0) | Ref. (0) |
| Only with sarcopenia | 1,410 | 0.03 (-0.02 to 0.07) | 0.02 (-0.03 to 0.06) | -0.03 (-0.07 to 0.002) |
| Only with obesity | 537 | -0.06 (-0.12 to -0.002)^*^ | -0.07 (-0.13 to -0.01)^*^ | -0.06 (-0.10 to -0.01)^*^ |
| Sarcopenic obesity | 769 | -0.02 (-0.07 to 0.03) | -0.04 (-0.09 to 0.01) | -0.08 (-0.12 to -0.04)^***^ |
| P for interaction |  | 0.80 | 0.78 | 0.68 |
| Sarcopenic obesity (BSA) |  |  |  |  |
| Without sarcopenia and obesity | 1,150 | Ref. (0) | Ref. (0) | Ref. (0) |
| Only with sarcopenia | 1,462 | 0.01 (-0.04 to 0.05) | -0.005 (-0.05 to 0.04) | -0.05 (-0.09 to -0.02)^**^ |
| Only with obesity | 650 | -0.03 (-0.08 to 0.03) | -0.02 (-0.08 to 0.03) | -0.02 (-0.06 to 0.03) |
| Sarcopenic obesity | 717 | 0.04 (-0.01 to 0.10) | 0.03 (-0.02 to 0.09) | -0.01 (-0.05 to 0.04) |
| P for interaction |  | 0.12 | 0.10 | 0.03 |
| Sarcopenic obesity (WHHR) |  |  |  |  |
| Without sarcopenia and obesity | 1,404 | Ref. (0) | Ref. (0) | Ref. (0) |
| Only with sarcopenia | 1,348 | 0.03 (-0.01 to 0.08) | 0.02 (-0.02 to 0.07) | -0.02 (-0.05 to 0.02) |
| Only with obesity | 396 | -0.04 (-0.11 to 0.02) | -0.05 (-0.11 to 0.02) | -0.05 (-0.10 to 0.004) |
| Sarcopenic obesity | 831 | -0.01 (-0.06 to 0.05) | -0.03 (-0.09 to 0.02) | -0.09 (-0.14 to -0.05)^***^ |
| P for interaction |  | 0.90 | 0.77 | 0.31 |
| Sarcopenic obesity (PFM) |  |  |  |  |
| Without sarcopenia and obesity | 1,241 | Ref. (0) | Ref. (0) | Ref. (0) |
| Only with sarcopenia | 1,385 | 0.02 (-0.03 to 0.06) | 0.003 (-0.04 to 0.05) | -0.04 (-0.08 to -0.01)^*^ |
| Only with obesity | 559 | -0.03 (-0.08 to 0.03) | -0.02 (-0.08 to 0.03) | -0.03 (-0.07 to 0.02) |
| Sarcopenic obesity | 794 | 0.03 (-0.02 to 0.08) | 0.02 (-0.03 to 0.07) | -0.03 (-0.07 to 0.01) |
| P for interaction |  | 0.30 | 0.28 | 0.25 |
| Sarcopenic obesity (PLM) |  |  |  |  |
| Without sarcopenia and obesity | 1,138 | Ref. (0) | Ref. (0) | Ref. (0) |
| Only with sarcopenia | 1,458 | 0.01 (-0.04 to 0.06) | -0.01 (-0.05 to 0.04) | -0.06 (-0.09 to -0.03)^**^ |
| Only with obesity | 662 | -0.03 (-0.09 to 0.02) | -0.03 (-0.09 to 0.02) | -0.03 (-0.07 to 0.01) |
| Sarcopenic obesity | 721 | 0.03 (-0.02 to 0.09) | 0.03 (-0.03 to 0.08) | -0.02 (-0.06 to 0.02) |
| P for interaction |  | 0.15 | 0.10 | 0.02 |
| Sarcopenic obesity (PPF) |  |  |  |  |
| Without sarcopenia and obesity | 1,348 | Ref. (0) | Ref. (0) | Ref. (0) |
| Only with sarcopenia | 1,341 | 0.02 (-0.03to 0.06) | 0.01 (-0.04 to 0.05) | -0.03 (-0.07 to 0.0003) |
| Only with obesity | 452 | -0.03 (-0.09 to 0.04) | -0.02 (-0.09 to 0.04) | -0.03 (-0.08 to 0.01) |
| Sarcopenic obesity | 838 | 0.03 (-0.02 to 0.09) | 0.01 (-0.04 to 0.07) | -0.05 (-0.09 to -0.01)^*^ |
| P for interaction |  | 0.32 | 0.43 | 0.57 |
| Sarcopenic obesity (CUN-BAE) |  |  |  |  |
| Without sarcopenia and obesity | 1,339 | Ref. (0) | Ref. (0) | Ref. (0) |
| Only with sarcopenia | 1,317 | 0.01 (-0.03 to 0.06) | -0.001 (-0.05 to 0.04) | -0.04 (-0.07 to -0.004)^*^ |
| Only with obesity | 461 | -0.05 (-0.11 to 0.01) | -0.05 (-0.11 to 0.01) | -0.04 (-0.09 to 0.01) |
| Sarcopenic obesity | 862 | 0.02 (-0.03 to 0.08) | 0.01 (-0.04 to 0.06) | -0.05 (-0.09 to -0.01)^*^ |
| P for interaction |  | 0.12 | 0.11 | 0.32 |

ABSI: a body shape index; BAI: body adiposity index; BMI: body mass index; BRI: body roundness index; BSA: body surface area; CI: confidence interval; CMI: cardiometabolic index; CUN-BAE: Clínica Universidad de Navarra-Body Adiposity Estimator; CVAI: Chinese visceral adiposity index; DWRT: Delayed Word Recall Test; HC, hip circumference; LAP: lipid accumulation product; PFM: predicted fat mass; PLM: predicted lean mass; PPF: predicated percent fat; Ref, reference; VAI: visceral adiposity index; WC: waist circumference; WHHR: waist-to-hip-to-height ratio; WHR: waist-to-hip ratio; WHtR: waist-to-height ratio

^a^: Adjusted for sex and age

^b^: Additionally adjusted for education, occupation, personal income, physical activity, drinking, smoking and self-rated health

^c^: Additionally adjusted for baseline DWRT score

^*^P <0.05, ^**^P <0.01, ^***^P <0.001

Table S8. Associations of baseline sarcopenia and sarcopenic obesity with annual change rate of memory function.

|  | *N* | Adjusted mean differences β (95% CI) in annual change rate of DWRT score | | |
| --- | --- | --- | --- | --- |
|  |  | Model 1^a^ | Model 2^b^ | Model 3^c^ |
| Sarcopenia |  |  |  |  |
| No | 1,790 | Ref. (0) | Ref. (0) | Ref. (0) |
| Yes | 2,151 | 0.61 (-0.34 to 1.55) | 0.26 (-0.68 to 1.21) | -0.79 (-1.57 to -0.01)^*^ |
| Sarcopenic obesity (Weight) |  |  |  |  |
| Without sarcopenia and obesity | 1,190 | Ref. (0) | Ref. (0) | Ref. (0) |
| Only with sarcopenia | 1,398 | 0.27 (-0.88 to 1.43) | -0.08 (-1.23 to 1.08) | -1.17 (-2.12 to -0.23)^*^ |
| Only with obesity | 600 | -0.75 (-2.16 to 0.67) | -0.83 (-2.23 to 0.58) | -0.96 (-2.10 to 0.19) |
| Sarcopenic obesity | 753 | 0.51 (-0.82 to 1.84) | 0.11 (-1.21 to 1.43) | -0.99 (-2.07 to 0.10) |
| P for interaction |  | 0.31 | 0.29 | 0.15 |
| Sarcopenic obesity (BMI) |  |  |  |  |
| Without sarcopenia and obesity | 1,240 | Ref. (0) | Ref. (0) | Ref. (0) |
| Only with sarcopenia | 1,322 | 0.81 (-0.34 to 1.96) | 0.48 (-0.67 to 1.63) | -0.67 (-1.61 to 0.27) |
| Only with obesity | 550 | 0.79 (-0.65 to 2.24) | 0.60 (-0.84 to 2.04) | 0.14 (-1.03 to 1.31) |
| Sarcopenic obesity | 829 | 0.91 (-0.38 to 2.21) | 0.40 (-0.90 to 1.70) | -0.87 (-1.93 to 0.20) |
| P for interaction |  | 0.48 | 0.48 | 0.67 |
| Sarcopenic obesity (WC) |  |  |  |  |
| Without sarcopenia and obesity | 1,367 | Ref. (0) | Ref. (0) | Ref. (0) |
| Only with sarcopenia | 1,454 | 0.85 (-0.24 to 1.94) | 0.54 (-0.55 to 1.64) | -0.51 (-1.41 to 0.38) |
| Only with obesity | 423 | -0.97 (-2.55 to 0.60) | -1.27 (-2.84 to 0.30) | -1.15 (-2.43 to 0.13) |
| Sarcopenic obesity | 697 | -0.75 (-2.13 to 0.62) | -1.49 (-2.88 to -0.10)^*^ | -2.44 (-3.57 to -1.30)^***^ |
| P for interaction |  | 0.54 | 0.45 | 0.36 |
| Sarcopenic obesity (HC) |  |  |  |  |
| Without sarcopenia and obesity | 1,268 | Ref. (0) | Ref. (0) | Ref. (0) |
| Only with sarcopenia | 1,473 | 0.34 (-0.78 to 1.46) | -0.06 (-1.18 to 1.06) | -1.20 (-2.16 to -0.29)^*^ |
| Only with obesity | 522 | -0.75 (-2.21 to 0.72) | -0.86 (-2.32 to 0.59) | -0.97 (-2.16 to 0.22) |
| Sarcopenic obesity | 678 | 0.50 (-0.87 to 1.86) | 0.18 (-1.19 to 1.54) | -0.80 (-1.92 to 0.31) |
| P for interaction |  | 0.36 | 0.27 | 0.09 |
| Sarcopenic obesity (WHR) |  |  |  |  |
| Without sarcopenia and obesity | 655 | Ref. (0) | Ref. (0) | Ref. (0) |
| Only with sarcopenia | 585 | 0.41 (-1.21 to 2.02) | 0.35 (-1.26 to 1.96) | -0.54 (-1.85 to 0.78) |
| Only with obesity | 1,135 | 0.03 (-1.38 to 1.44) | -0.08 (-1.48 to 1.32) | -0.55 (-1.69 to 0.59) |
| Sarcopenic obesity | 1,566 | 0.72 (-0.67 to 2.12) | 0.15 (-1.25 to 1.55) | -1.43 (-2.57 to -0.28)^*^ |
| P for interaction |  | 0.77 | 0.91 | 0.67 |
| Sarcopenic obesity (WHtR) |  |  |  |  |
| Without sarcopenia and obesity | 1,143 | Ref. (0) | Ref. (0) | Ref. (0) |
| Only with sarcopenia | 1,073 | 0.77 (-0.44 to 1.99) | 0.52 (-0.69 to 1.73) | -0.51 (-1.50 to 0.48) |
| Only with obesity | 647 | -0.32 (-1.71 to 1.07) | -0.65 (-2.04 to 0.74) | -0.97 (-2.10 to 0.16) |
| Sarcopenic obesity | 1,078 | 0.15 (-1.12 to 1.42) | -0.59 (-1.88 to 0.69) | -1.95 (-3.00 to -0.90)^***^ |
| P for interaction |  | 0.75 | 0.62 | 0.54 |
| Sarcopenic obesity (LAP) |  |  |  |  |
| Without sarcopenia and obesity | 1,274 | Ref. (0) | Ref. (0) | Ref. (0) |
| Only with sarcopenia | 1,398 | 0.75 (-0.37 to 1.87) | 0.42 (-0.71 to 1.54) | -0.61 (-1.53 to 0.31) |
| Only with obesity | 516 | -0.90 (-2.38 to 0.57) | -1.16 (-2.63 to 0.30) | -0.89 (-2.09 to 0.30) |
| Sarcopenic obesity | 753 | -0.46 (-1.80 to 0.88) | -1.08 (-2.43 to 0.26) | -1.95 (-3.05 to -0.85)^**^ |
| P for interaction |  | 0.76 | 0.73 | 0.58 |
| Sarcopenic obesity (ABSI) |  |  |  |  |
| Without sarcopenia and obesity | 1,346 | Ref. (0) | Ref. (0) | Ref. (0) |
| Only with sarcopenia | 1,412 | 0.96 (-0.14 to 2.05) | 0.69 (-0.40 to 1.79) | -0.38 (-1.27 to 0.52) |
| Only with obesity | 444 | 0.45 (-1.10 to 1.99) | 0.36 (-1.18 to 1.90) | 0.10 (-1.16 to 1.36) |
| Sarcopenic obesity | 739 | 0.16 (-1.23 to 1.55) | -0.49 (-1.89 to 0.92) | -1.75 (-2.90 to -0.60)^**^ |
| P for interaction |  | 0.22 | 0.13 | 0.08 |
| Sarcopenic obesity (VAI) |  |  |  |  |
| Without sarcopenia and obesity | 1,236 | Ref. (0) | Ref. (0) | Ref. (0) |
| Only with sarcopenia | 1,418 | 0.82 (-0.32 to 1.95) | 0.50 (-0.63 to 1.63) | -0.60 (-1.53 to 0.32) |
| Only with obesity | 554 | -0.60 (-2.04 to 0.84) | -0.78 (-2.22 to 0.65) | -0.44 (-1.60 to 0.73) |
| Sarcopenic obesity | 733 | -0.38 (-1.73 to 0.97) | -0.98 (-2.34 to 0.38) | -1.59 (-2.71 to -0.48)^**^ |
| P for interaction |  | 0.54 | 0.48 | 0.48 |
| Sarcopenic obesity (CVAI) |  |  |  |  |
| Without sarcopenia and obesity | 1,356 | Ref. (0) | Ref. (0) | Ref. (0) |
| Only with sarcopenia | 1,344 | 1.00 (-0.11 to 2.10) | 0.69 (-0.42 to 1.79) | -0.42 (-1.32 to 0.49) |
| Only with obesity | 434 | -0.76 (-2.32 to 0.81) | -0.97 (-2.53 to 0.59) | -0.65 (-1.92 to 0.63) |
| Sarcopenic obesity | 807 | -0.84 (-2.20 to 0.52) | -1.46 (-2.83 to -0.09)^*^ | -2.13 (-3.24 to -1.01)^***^ |
| P for interaction |  | 0.29 | 0.24 | 0.20 |
| Sarcopenic obesity (BRI) |  |  |  |  |
| Without sarcopenia and obesity | 1,350 | Ref. (0) | Ref. (0) | Ref. (0) |
| Only with sarcopenia | 1,342 | 0.63 (-0.48 to 1.74) | 0.35 (-0.76 to 1.46) | -0.62 (-1.53 to 0.28) |
| Only with obesity | 440 | -0.90 (-2.45 to 0.65) | -1.21 (-2.76 to 0.34) | -1.08 (-2.34 to 0.19) |
| Sarcopenic obesity | 809 | -0.13 (-1.46 to 1.20) | -0.87 (-2.22 to 0.47) | -1.99 (-3.09 to -0.89)^***^ |
| P for interaction |  | 0.89 | 0.99 | 0.72 |
| Sarcopenic obesity (Conicity index) |  |  |  |  |
| Without sarcopenia and obesity | 1,359 | Ref. (0) | Ref. (0) | Ref. (0) |
| Only with sarcopenia | 1,427 | 0.90 (-0.19 to 1.99) | 0.66 (-0.43 to 1.75) | -0.58 (-1.47 to 0.31) |
| Only with obesity | 431 | 1.40 (-0.17 to 2.96) | 1.37 (-0.19 to 2.93( | 0.82 (-0.45 to 2.09) |
| Sarcopenic obesity | 724 | 1.13 (-0.27 to 2.54) | 0.52 (-0.90 to 1.95) | -0.57 (-1.73 to 0.59) |
| P for interaction |  | 0.26 | 0.14 | 0.33 |
| Sarcopenic obesity (BAI) |  |  |  |  |
| Without sarcopenia and obesity | 1,312 | Ref. (0) | Ref. (0) | Ref. (0) |
| Only with sarcopenia | 1,341 | 0.70 (-0.42 to 1.82) | 0.35 (-0.77 to 1.47) | -0.73 (-1.64 to 0.19) |
| Only with obesity | 478 | 0.21 (-1.29 to 1.72) | -0.13 (-1.63 to 1.37) | -0.02 (-1.25 to 1.20) |
| Sarcopenic obesity | 810 | 0.60 (-0.72 to 1.93) | -0.01 (-1.34 to 1.32) | -0.93 (-2.02 to 0.16) |
| P for interaction |  | 0.76 | 0.81 | 0.82 |
| Sarcopenic obesity (CMI) |  |  |  |  |
| Without sarcopenia and obesity | 1,258 | Ref. (0) | Ref. (0) | Ref. (0) |
| Only with sarcopenia | 1,394 | 0.83 (-0.30 to 1.96) | 0.48 (-0.65 to 1.61) | -0.58 (-1.50 to 0.34) |
| Only with obesity | 532 | -0.73 (-2.18 to 0.73) | -1.02 (-2.47 to 0.43) | -0.59 (-1.77 to 0.59) |
| Sarcopenic obesity | 757 | -0.46 (-1.80 to 0.87) | -1.10 (-2.44 to 0.25) | -1.74 (-2.84 to -0.64)^**^ |
| P for interaction |  | 0.57 | 0.56 | 0.47 |
| Sarcopenic obesity (BSA) |  |  |  |  |
| Without sarcopenia and obesity | 1,142 | Ref. (0) | Ref. (0) | Ref. (0) |
| Only with sarcopenia | 1,444 | 0.25 (-0.90 to 1.41) | -0.08 (-1.24 to 1.08) | -1.22 (-2.17 to -0.27)^*^ |
| Only with obesity | 648 | -0.78 (-2.17 to 0.61) | -0.75 (-2.13 to 0.63) | -0.71 (-1.84 to 0.41) |
| Sarcopenic obesity | 707 | 0.47 (-0.89 to 1.83) | 0.14 (-1.21 to 1.50) | -0.72 (-1.82 to 0.39) |
| P for interaction |  | 0.30 | 0.31 | 0.12 |
| Sarcopenic obesity (WHHR) |  |  |  |  |
| Without sarcopenia and obesity | 1,396 | Ref. (0) | Ref. (0) | Ref. (0) |
| Only with sarcopenia | 1,334 | 0.90 (-0.20 to 2.00) | 0.69 (-0.41 to 1.78) | -0.26 (-1.16 to 0.63) |
| Only with obesity | 394 | -0.68 (-2.30 to 0.93) | -0.95 (-2.56 to 0.67) | -0.99 (-2.30 to 0.33) |
| Sarcopenic obesity | 817 | -0.50 (-1.84 to 0.85) | -1.38 (-2.74 to -0.01)^*^ | -2.70 (-3.81 to -1.59)^***^ |
| P for interaction |  | 0.49 | 0.28 | 0.09 |
| Sarcopenic obesity (PFM) |  |  |  |  |
| Without sarcopenia and obesity | 1,235 | Ref. (0) | Ref. (0) | Ref. (0) |
| Only with sarcopenia | 1,368 | 0.15 (-1.00 to 1.29) | -0.18 (-1.32 to 0.96) | -1.17 (-2.10 to -0.23)^*^ |
| Only with obesity | 555 | -1.23 (-2.67 to 0.21) | -1.34 (-2.77 to 0.09) | -1.29 (-2.46 to -0.12)^*^ |
| Sarcopenic obesity | 783 | 0.35 (-0.97 to 1.67) | -0.11 (-1.43 to 1.21) | -1.24 (-2.32 to -0.16)^*^ |
| P for interaction |  | 0.14 | 0.14 | 0.12 |
| Sarcopenic obesity (PLM) |  |  |  |  |
| Without sarcopenia and obesity | 1,130 | Ref. (0) | Ref. (0) | Ref. (0) |
| Only with sarcopenia | 1,439 | 0.25 (-0.92 to 1.41) | -0.12 (-1.28 to 1.05) | -1.29 (-2.24 to -0.33)^**^ |
| Only with obesity | 660 | -0.88 (-2.27 to 0.50) | -0.88 (-2.26 to 0.49) | -0.99 (-2.11 to 0.14) |
| Sarcopenic obesity | 712 | 0.37 (-0.98 to 1.72) | 0.07 (-1.28 to 1.42) | -0.89 (-1.99 to 0.22) |
| P for interaction |  | 0.30 | 0.26 | 0.08 |
| Sarcopenic obesity (PPF) |  |  |  |  |
| Without sarcopenia and obesity | 1,343 | Ref. (0) | Ref. (0) | Ref. (0) |
| Only with sarcopenia | 1,328 | 0.24 (-0.87 to 1.36) | -0.02 (-1.14 to 1.09) | -0.96 (-1.87 to -0.04)^*^ |
| Only with obesity | 447 | -1.31 (-2.85 to 0.24) | -1.44 (-2.98 to 0.10) | -1.47 (-2.72 to -0.21) |
| Sarcopenic obesity | 823 | 0.30 (-1.03 to 1.63) | -0.30 (-1.63 to 1.03) | -1.61 (-2.70 to -0.53)^**^ |
| P for interaction |  | 0.18 | 0.25 | 0.32 |
| Sarcopenic obesity (CUN-BAE) |  |  |  |  |
| Without sarcopenia and obesity | 1,330 | Ref. (0) | Ref. (0) | Ref. (0) |
| Only with sarcopenia | 1,303 | 0.28 (-0.85 to 1.40) | -0.06 (-1.18 to 1.07) | -0.95 (-1.87 to -0.03)^*^ |
| Only with obesity | 460 | -1.20 (-2.73 to 0.34) | -1.44 (-2.96 to 0.09) | -1.33 (-2.58 to -0.09)^*^ |
| Sarcopenic obesity | 848 | 0.28 (-1.04 to 1.60) | -0.26 (-1.58 to 1.06) | -1.53 (-2.62 to -0.45)^**^ |
| P for interaction |  | 0.23 | 0.22 | 0.36 |

ABSI: a body shape index; BAI: body adiposity index; BMI: body mass index; BRI: body roundness index; BSA: body surface area; CI: confidence interval; CMI: cardiometabolic index; CUN-BAE: Clínica Universidad de Navarra-Body Adiposity Estimator; CVAI: Chinese visceral adiposity index; DWRT: Delayed Word Recall Test; HC, hip circumference; LAP: lipid accumulation product; PFM: predicted fat mass; PLM: predicted lean mass; PPF: predicated percent fat; Ref, reference; VAI: visceral adiposity index; WC: waist circumference; WHHR: waist-to-hip-to-height ratio; WHR: waist-to-hip ratio; WHtR: waist-to-height ratio

^a^: Adjusted for sex and age

^b^: Additionally adjusted for education, occupation, personal income, physical activity, drinking, smoking and self-rated health

^c^: Additionally adjusted for baseline DWRT score

^*^P <0.05, ^**^P <0.01, ^***^P <0.001

Table S9. Associations of baseline sarcopenia and sarcopenic obesity with memory function at baseline by sex.

|  | Adjusted mean differences β (95% CI) in baseline DWRT score | | |
| --- | --- | --- | --- |
|  | Women | Men | P for interaction |
| Sarcopenia |  |  | 0.24 |
| No | Ref. (0) | Ref. (0) |  |
| Yes | -0.22 (-0.34 to -0.11)^***^ | -0.26 (-0.46 to -0.06)^*^ |  |
| Sarcopenic obesity (Weight) |  |  | 0.02 |
| Without sarcopenia and obesity | Ref. (0) | Ref. (0) |  |
| Only with sarcopenia | -0.22 (-0.35 to -0.08)^**^ | -0.37 (-0.61 to -0.13)^**^ |  |
| Only with obesity | -0.01 (-0.18 to 0.16) | 0.05 (-0.24 to 0.34) |  |
| Sarcopenic obesity | -0.24 (-0.39 to -0.09)^**^ | -0.02 (-0.30 to 0.26) |  |
| Sarcopenic obesity (BMI) |  |  | 0.56 |
| Without sarcopenia and obesity | Ref. (0) | Ref. (0) |  |
| Only with sarcopenia | -0.21 (-0.35 to -0.08)^**^ | -0.29 (-0.53 to -0.05)^*^ |  |
| Only with obesity | 0.01 (-0.17 to 0.18) | -0.01 (-0.31 to 0.30) |  |
| Sarcopenic obesity | -0.24 (-0.38 to -0.09)^**^ | -0.21 (-0.49 to 0.07) |  |
| Sarcopenic obesity (WC) |  |  | 0.60 |
| Without sarcopenia and obesity | Ref. (0) | Ref. (0) |  |
| Only with sarcopenia | -0.21 (-0.34 to -0.08)^**^ | -0.28 (-0.50 to -0.07)^**^ |  |
| Only with obesity | 0.04 (-0.13 to 0.22) | -0.12 (-0.54 to 0.29) |  |
| Sarcopenic obesity | -0.21 (-0.36 to -0.05)^**^ | -0.23 (-0.57 to 0.10) |  |
| Sarcopenic obesity (HC) |  |  | 0.21 |
| Without sarcopenia and obesity | Ref. (0) | Ref. (0) |  |
| Only with sarcopenia | -0.21 (-0.34 to -0.08)^**^ | -0.35 (-0.59 to -0.12)^**^ |  |
| Only with obesity | 0.05 (-0.13 to 0.22) | -0.10 (-0.41 to 0.20) |  |
| Sarcopenic obesity | -0.21 (-0.37 to -0.05)^**^ | -0.13 (-0.42 to 0.16) |  |
| Sarcopenic obesity (WHR) |  |  | 0.55 |
| Without sarcopenia and obesity | Ref. (0) | Ref. (0) |  |
| Only with sarcopenia | -0.17 (-0.39 to 0.04) | -0.27 (-0.54 to 0.005) |  |
| Only with obesity | -0.13 (-0.31 to 0.05) | -0.08 (-0.36 to 0.20) |  |
| Sarcopenic obesity | -0.36 (-0.54 to -0.19)^***^ | -0.32 (-0.59 to -0.06)^*^ |  |
| Sarcopenic obesity (WHtR) |  |  | 0.69 |
| Without sarcopenia and obesity | Ref. (0) | Ref. (0) |  |
| Only with sarcopenia | -0.21 (-0.35 to -0.07)^**^ | -0.25 (-0.50 to 0.01) |  |
| Only with obesity | -0.06 (-0.23 to 0.11) | -0.07 (-0.35 to 0.22) |  |
| Sarcopenic obesity | -0.29 (-0.44 to -0.14)^***^ | -0.33 (-0.60 to -0.07)^*^ |  |
| Sarcopenic obesity (LAP) |  |  | 0.70 |
| Without sarcopenia and obesity | Ref. (0) | Ref. (0) |  |
| Only with sarcopenia | -0.19 (-0.33 to -0.06)^**^ | -0.24 (-0.48 to -0.004)^*^ |  |
| Only with obesity | 0.05 (-0.13 to 0.23) | 0.01 (-0.29 to 0.30) |  |
| Sarcopenic obesity | -0.24 (-0.39 to -0.08)^**^ | -0.28 (-0.56 to -0.002)^*^ |  |
| Sarcopenic obesity (ABSI) |  |  | 0.39 |
| Without sarcopenia and obesity | Ref. (0) | Ref. (0) |  |
| Only with sarcopenia | -0.23 (-0.36 to -0.10)^***^ | -0.23 (-0.47 to 0.003) |  |
| Only with obesity | -0.10 (-0.28 to 0.09) | -0.13 (-0.44 to 0.18) |  |
| Sarcopenic obesity | -0.30 (-0.46 to -0.14)^***^ | -0.44 (-0.72 to -0.16)^**^ |  |
| Sarcopenic obesity (VAI) |  |  | 0.37 |
| Without sarcopenia and obesity | Ref. (0) | Ref. (0) |  |
| Only with sarcopenia | -0.21 (-0.35 to -0.08)^**^ | -0.19 (-0.43 to 0.05) |  |
| Only with obesity | 0.02 (-0.15 to 0.19) | 0.20 (-0.09 to 0.50) |  |
| Sarcopenic obesity | -0.23 (-0.38 to -0.07)^**^ | -0.20 (-0.48 to 0.07) |  |
| Sarcopenic obesity (CVAI) |  |  | 0.09 |
| Without sarcopenia and obesity | Ref. (0) | Ref. (0) |  |
| Only with sarcopenia | -0.17 (-0.30 to -0.04)^*^ | -0.37 (-0.60 to -0.13)^**^ |  |
| Only with obesity | 0.16 (-0.03 to 0.36) | -0.17 (-0.48 to 0.13) |  |
| Sarcopenic obesity | -0.21 (-0.36 to -0.05)^*^ | -0.21 (-0.49 to 0.06) |  |
| Sarcopenic obesity (BRI) |  |  | 0.29 |
| Without sarcopenia and obesity | Ref. (0) | Ref. (0) |  |
| Only with sarcopenia | -0.21 (-0.34 to -0.08)^**^ | -0.30 (-0.54 to -0.07)^*^ |  |
| Only with obesity | 0.01 (-0.18 to 0.20) | -0.25 (-0.55 to 0.06) |  |
| Sarcopenic obesity | -0.25 (-0.40 to -0.09)^**^ | -0.39 (-0.66 to -0.12)^**^ |  |
| Sarcopenic obesity (Conicity index) |  |  | 0.008 |
| Without sarcopenia and obesity | Ref. (0) | Ref. (0) |  |
| Only with sarcopenia | -0.22 (-0.35 to -0.09)^***^ | -0.25 (-0.48 to -0.02)^*^ |  |
| Only with obesity | -0.06 (-0.25 to 0.12) | -0.35 (-0.66 to -0.03)^*^ |  |
| Sarcopenic obesity | -0.28 (-0.44 to -0.12)^***^ | -0.62 (-0.91 to -0.34)^***^ |  |
| Sarcopenic obesity (BAI) |  |  | 0.24 |
| Without sarcopenia and obesity | Ref. (0) | Ref. (0) |  |
| Only with sarcopenia | -0.21 (-0.34 to -0.08)^**^ | -0.32 (-0.55 to -0.08)^**^ |  |
| Only with obesity | 0.09 (-0.10 to 0.27) | -0.20 (-0.51 to 0.10) |  |
| Sarcopenic obesity | -0.19 (-0.34 to -0.04)^*^ | -0.33 (-0.61 to -0.06)^*^ |  |
| Sarcopenic obesity (CMI) |  |  | 0.35 |
| Without sarcopenia and obesity | Ref. (0) | Ref. (0) |  |
| Only with sarcopenia | -0.21 (-0.34 to -0.08)^**^ | -0.18 (-0.42 to 0.05) |  |
| Only with obesity | 0.01 (-0.16 to 0.19) | 0.21 (-0.09 to 0.51) |  |
| Sarcopenic obesity | -0.23 (-0.39 to -0.08)^**^ | -0.21 (-0.49 to 0.06) |  |
| Sarcopenic obesity (BSA) |  |  | 0.29 |
| Without sarcopenia and obesity | Ref. (0) | Ref. (0) |  |
| Only with sarcopenia | -0.25 (-0.39 to -0.12)^***^ | -0.32 (-0.56 to -0.08)^**^ |  |
| Only with obesity | -0.001 (-0.17 to 0.17) | 0.07 (-0.22 to 0.36) |  |
| Sarcopenic obesity | -0.17 (-0.32 to -0.01)^*^ | -0.08 (-0.37 to 0.20) |  |
| Sarcopenic obesity (WHHR) |  |  | 0.63 |
| Without sarcopenia and obesity | Ref. (0) | Ref. (0) |  |
| Only with sarcopenia | -0.17 (-0.30 to -0.04)^*^ | -0.21 (-0.44 to 0.03) |  |
| Only with obesity | 0.001 (-0.20 to 0.20) | -0.06 (-0.37 to 0.26) |  |
| Sarcopenic obesity | -0.34 (-0.50 to -0.19)^***^ | -0.40 (-0.68 to -0.13)^**^ |  |
| Sarcopenic obesity (PFM) |  |  | 0.29 |
| Without sarcopenia and obesity | Ref. (0) | Ref. (0) |  |
| Only with sarcopenia | -0.18 (-0.31 to -0.04)^**^ | -0.33 (-0.57 to -0.09)^**^ |  |
| Only with obesity | 0.08 (-0.10 to 0.26) | -0.11 (-0.41 to 0.18) |  |
| Sarcopenic obesity | -0.24 (-0.39 to -0.08)^**^ | -0.24 (-0.52 to 0.04) |  |
| Sarcopenic obesity (PLM) |  |  | 0.10 |
| Without sarcopenia and obesity | Ref. (0) | Ref. (0) |  |
| Only with sarcopenia | -0.26 (-0.40 to -0.12)^***^ | -0.35 (-0.59 to -0.10)^**^ |  |
| Only with obesity | -0.03 (-0.19 to 0.14) | 0.07 (-0.22 to 0.36) |  |
| Sarcopenic obesity | -0.19 (-0.34 to -0.03)^*^ | -0.03 (-0.32 to 0.26) |  |
| Sarcopenic obesity (PPF) |  |  | 0.16 |
| Without sarcopenia and obesity | Ref. (0) | Ref. (0) |  |
| Only with sarcopenia | -0.16 (-0.29 to -0.03)^*^ | -0.31 (-0.55 to -0.08)^**^ |  |
| Only with obesity | 0.10 (-0.09 to 0.29) | -0.26 (-0.56 to 0.05) |  |
| Sarcopenic obesity | -0.28 (-0.43 to -0.12)^***^ | -0.37 (-0.65 to -0.10)^**^ |  |
| Sarcopenic obesity (CUN-BAE) |  |  | 0.28 |
| Without sarcopenia and obesity | Ref. (0) | Ref. (0) |  |
| Only with sarcopenia | -0.16 (-0.29 to -0.03)^*^ | -0.32 (-0.56 to -0.08)^**^ |  |
| Only with obesity | 0.13 (-0.06 to 0.32) | -0.11 (-0.42 to 0.20) |  |
| Sarcopenic obesity | -0.25 (-0.40 to -0.09)^**^ | -0.24 (-0.52 to 0.03) |  |

ABSI: a body shape index; BAI: body adiposity index; BMI: body mass index; BRI: body roundness index; BSA: body surface area; CI: confidence interval; CMI: cardiometabolic index; CUN-BAE: Clínica Universidad de Navarra-Body Adiposity Estimator; CVAI: Chinese visceral adiposity index; DWRT: Delayed Word Recall Test; HC, hip circumference; LAP: lipid accumulation product; PFM: predicted fat mass; PLM: predicted lean mass; PPF: predicated percent fat; Ref, reference; VAI: visceral adiposity index; WC: waist circumference; WHHR: waist-to-hip-to-height ratio; WHR: waist-to-hip ratio; WHtR: waist-to-height ratio

Note: Adjusted for sex, age, education, occupation, personal income, physical activity, drinking, smoking and self-rated health

^*^P <0.05, ^**^P <0.01, ^***^P <0.001

Table S10. Associations of baseline sarcopenia and sarcopenic obesity with memory function at baseline by age.

|  | Adjusted mean differences β (95% CI) in baseline DWRT score | | |
| --- | --- | --- | --- |
|  | ≤60 | >60 | P for interaction |
| Sarcopenia |  |  | 0.17 |
| No | Ref. (0) | Ref. (0) |  |
| Yes | -0.20 (-0.31 to -0.08)^***^ | -0.28 (-0.45 to -0.11)^**^ |  |
| Sarcopenic obesity (Weight) |  |  | 0.19 |
| Without sarcopenia and obesity | Ref. (0) | Ref. (0) |  |
| Only with sarcopenia | -0.17 (-0.31 to -0.02)^*^ | -0.34 (-0.54 to -0.14)^***^ |  |
| Only with obesity | 0.03 (-0.14 to 0.20) | -0.07 (-0.36 to 0.22) |  |
| Sarcopenic obesity | -0.22 (-0.39 to -0.05)^*^ | -0.23 (-0.45 to -0.004)^*^ |  |
| Sarcopenic obesity (BMI) |  |  | 0.26 |
| Without sarcopenia and obesity | Ref. (0) | Ref. (0) |  |
| Only with sarcopenia | -0.15 (-0.30 to -0.01)^*^ | -0.33 (-0.54 to -0.13)^**^ |  |
| Only with obesity | 0.04 (-0.13 to 0.21) | -0.14 (-0.43 to 0.16) |  |
| Sarcopenic obesity | -0.24 (-0.41 to -0.07)^**^ | -0.31 (-0.53 to -0.09)^**^ |  |
| Sarcopenic obesity (WC) |  |  | 0.44 |
| Without sarcopenia and obesity | Ref. (0) | Ref. (0) |  |
| Only with sarcopenia | -0.22 (-0.35 to -0.08)^**^ | -0.25 (-0.45 to -0.05)^*^ |  |
| Only with obesity | -0.04 (-0.22 to 0.15) | 0.05 (-0.27 to 0.36) |  |
| Sarcopenic obesity | -0.18 (-0.37 to 0.001) | -0.30 (-0.53 to -0.08)^**^ |  |
| Sarcopenic obesity (HC) |  |  | 0.36 |
| Without sarcopenia and obesity | Ref. (0) | Ref. (0) |  |
| Only with sarcopenia | -0.22 (-0.36 to -0.08)^**^ | -0.26 (-0.45 to -0.06)^**^ |  |
| Only with obesity | -0.05 (-0.22 to 0.13) | 0.13 (-0.18 to 0.44) |  |
| Sarcopenic obesity | -0.19 (-0.37 to -0.02)^*^ | -0.22 (-0.44 to 0.01) |  |
| Sarcopenic obesity (WHR) |  |  | 0.08 |
| Without sarcopenia and obesity | Ref. (0) | Ref. (0) |  |
| Only with sarcopenia | -0.29 (-0.50 to -0.09)^**^ | -0.06 (-0.36 to 0.23) |  |
| Only with obesity | -0.21 (-0.38 to -0.05)^*^ | 0.03 (-0.26 to 0.32) |  |
| Sarcopenic obesity | -0.36 (-0.53 to -0.19)^***^ | -0.33 (-0.60 to -0.06)^*^ |  |
| Sarcopenic obesity (WHtR) |  |  | 0.49 |
| Without sarcopenia and obesity | Ref. (0) | Ref. (0) |  |
| Only with sarcopenia | -0.22 (-0.37 to -0.07)^**^ | -0.23 (-0.45 to 0.003) |  |
| Only with obesity | -0.13 (-0.29 to 0.04) | -0.02 (-0.30 to 0.25) |  |
| Sarcopenic obesity | -0.27 (-0.44 to -0.11)^**^ | -0.35 (-0.57 to -0.12)^**^ |  |
| Sarcopenic obesity (LAP) |  |  | 0.55 |
| Without sarcopenia and obesity | Ref. (0) | Ref. (0) |  |
| Only with sarcopenia | -0.16 (-0.30 to -0.02)^*^ | -0.26 (-0.46 to -0.06)^*^ |  |
| Only with obesity | 0.01 (-0.17 to 0.19) | 0.03 (-0.26 to 0.32) |  |
| Sarcopenic obesity | -0.27 (-0.44 to -0.09)^**^ | -0.29 (-0.51 to -0.07)^*^ |  |
| Sarcopenic obesity (ABSI) |  |  | 0.11 |
| Without sarcopenia and obesity | Ref. (0) | Ref. (0) |  |
| Only with sarcopenia | -0.25 (-0.39 to -0.12)^***^ | -0.21 (-0.42 to 0.002) |  |
| Only with obesity | -0.22 (-0.41 to -0.03)^*^ | 0.02 (-0.26 to 0.30) |  |
| Sarcopenic obesity | -0.25 (-0.44 to -0.05)^*^ | -0.37 (-0.59 to -0.15)^**^ |  |
| Sarcopenic obesity (VAI) |  |  | 0.30 |
| Without sarcopenia and obesity | Ref. (0) | Ref. (0) |  |
| Only with sarcopenia | -0.20 (-0.34 to -0.06)^**^ | -0.18 (-0.39 to 0.02) |  |
| Only with obesity | -0.01 (-0.19 to 0.16) | 0.22 (-0.06 to 0.51) |  |
| Sarcopenic obesity | -0.20 (-0.38 to -0.02)^*^ | -0.24 (-0.46 to -0.01)^*^ |  |
| Sarcopenic obesity (CVAI) |  |  | 0.56 |
| Without sarcopenia and obesity | Ref. (0) | Ref. (0) |  |
| Only with sarcopenia | -0.20 (-0.34 to -0.07)^**^ | -0.25 (-0.46 to -0.03)^*^ |  |
| Only with obesity | -0.05 (-0.25 to 0.15) | 0.11 (-0.17 to 0.38) |  |
| Sarcopenic obesity | -0.23 (-0.42 to -0.04)^*^ | -0.23 (-0.45 to -0.01)^*^ |  |
| Sarcopenic obesity (BRI) |  |  | 0.24 |
| Without sarcopenia and obesity | Ref. (0) | Ref. (0) |  |
| Only with sarcopenia | -0.24 (-0.38 to -0.10)^***^ | -0.22 (-0.43 to -0.01)^*^ |  |
| Only with obesity | -0.18 (-0.37 to 0.01) | 0.05 (-0.24 to 0.34) |  |
| Sarcopenic obesity | -0.24 (-0.42 to -0.06)^*^ | -0.32 (-0.54 to -0.11)^**^ |  |
| Sarcopenic obesity (Conicity index) |  |  | 0.05 |
| Without sarcopenia and obesity | Ref. (0) | Ref. (0) |  |
| Only with sarcopenia | -0.26 (-0.39 to -0.12)^***^ | -0.24 (-0.45 to -0.03)^*^ |  |
| Only with obesity | -0.22 (-0.41 to -0.02)^*^ | -0.09 (-0.37 to 0.19) |  |
| Sarcopenic obesity | -0.21 (-0.41 to -0.01)^*^ | -0.44 (-0.66 to -0.21)^***^ |  |
| Sarcopenic obesity (BAI) |  |  | 0.33 |
| Without sarcopenia and obesity | Ref. (0) | Ref. (0) |  |
| Only with sarcopenia | -0.22 (-0.35 to -0.08)^**^ | -0.25 (-0.45 to -0.04)^*^ |  |
| Only with obesity | -0.09 (-0.27 to 0.09) | 0.14 (-0.15 to 0.43) |  |
| Sarcopenic obesity | -0.23 (-0.41 to -0.05)^*^ | -0.21 (-0.43 to 0.004) |  |
| Sarcopenic obesity (CMI) |  |  | 0.25 |
| Without sarcopenia and obesity | Ref. (0) | Ref. (0) |  |
| Only with sarcopenia | -0.21 (-0.35 to -0.07)^**^ | -0.18 (-0.38 to 0.03) |  |
| Only with obesity | -0.03 (-0.20 to 0.15) | 0.22 (-0.06 to 0.51) |  |
| Sarcopenic obesity | -0.20 (-0.38 to -0.02)^*^ | -0.25 (-0.47 to -0.03)^*^ |  |
| Sarcopenic obesity (BSA) |  |  | 0.52 |
| Without sarcopenia and obesity | Ref. (0) | Ref. (0) |  |
| Only with sarcopenia | -0.23 (-0.38 to -0.08)^**^ | -0.29 (-0.48 to -0.09)^**^ |  |
| Only with obesity | -0.001 (-0.17 to 0.16) | 0.09 (-0.21 to 0.38) |  |
| Sarcopenic obesity | -0.14 (-0.32 to 0.03) | -0.19 (-0.42 to 0.04) |  |
| Sarcopenic obesity (WHHR) |  |  | 0.78 |
| Without sarcopenia and obesity | Ref. (0) | Ref. (0) |  |
| Only with sarcopenia | -0.16 (-0.30 to -0.03)^*^ | -0.21 (-0.42 to 0.001) |  |
| Only with obesity | -0.09 (-0.29 to 0.12) | -0.02 (-0.31 to 0.27) |  |
| Sarcopenic obesity | -0.37 (-0.56 to -0.18)^***^ | -0.38 (-0.60 to -0.16)^***^ |  |
| Sarcopenic obesity (PFM) |  |  | 0.31 |
| Without sarcopenia and obesity | Ref. (0) | Ref. (0) |  |
| Only with sarcopenia | -0.14 (-0.29 to 0.0002) | -0.31 (-0.51 to -0.10)^**^ |  |
| Only with obesity | 0.05 (-0.13 to 0.22) | -0.08 (-0.37 to 0.22) |  |
| Sarcopenic obesity | -0.25 (-0.42 to -0.08)^**^ | -0.30 (-0.52 to -0.08)^**^ |  |
| Sarcopenic obesity (PLM) |  |  | 0.48 |
| Without sarcopenia and obesity | Ref. (0) | Ref. (0) |  |
| Only with sarcopenia | -0.21 (-0.36 to -0.06)^**^ | -0.33 (-0.53 to -0.13)^**^ |  |
| Only with obesity | 0.02 (-0.14 to 0.19) | -0.03 (-0.33 to 0.26) |  |
| Sarcopenic obesity | -0.15 (-0.32 to 0.02) | -0.19 (-0.42 to 0.04) |  |
| Sarcopenic obesity (PPF) |  |  | 0.54 |
| Without sarcopenia and obesity | Ref. (0) | Ref. (0) |  |
| Only with sarcopenia | -0.16 (-0.30 to -0.02)^*^ | -0.28 (-0.48 to -0.07)^**^ |  |
| Only with obesity | -0.001 (-0.20 to 0.19) | -0.13 (-0.41 to 0.16) |  |
| Sarcopenic obesity | -0.29 (-0.47 to -0.11)^**^ | -0.39 (-0.60 to -0.17)^***^ |  |
| Sarcopenic obesity (CUN-BAE) |  |  | 0.15 |
| Without sarcopenia and obesity | Ref. (0) | Ref. (0) |  |
| Only with sarcopenia | -0.15 (-0.28 to -0.01)^*^ | -0.33 (-0.54 to -0.12)^**^ |  |
| Only with obesity | 0.13 (-0.06 to 0.33) | -0.19 (-0.47 to 0.09) |  |
| Sarcopenic obesity | -0.21 (-0.39 to -0.03)^*^ | -0.38 (-0.60 to -0.16)^***^ |  |

ABSI: a body shape index; BAI: body adiposity index; BMI: body mass index; BRI: body roundness index; BSA: body surface area; CI: confidence interval; CMI: cardiometabolic index; CUN-BAE: Clínica Universidad de Navarra-Body Adiposity Estimator; CVAI: Chinese visceral adiposity index; DWRT: Delayed Word Recall Test; HC, hip circumference; LAP: lipid accumulation product; PFM: predicted fat mass; PLM: predicted lean mass; PPF: predicated percent fat; Ref, reference; VAI: visceral adiposity index; WC: waist circumference; WHHR: waist-to-hip-to-height ratio; WHR: waist-to-hip ratio; WHtR: waist-to-height ratio

Note: Adjusted for sex, age, education, occupation, personal income, physical activity, drinking, smoking and self-rated health

^*^P <0.05, ^**^P <0.01, ^***^P <0.001

Table S11. Associations of baseline sarcopenia and sarcopenic obesity with memory function at baseline by baseline memory status.

|  | Adjusted mean differences β (95% CI) in baseline DWRT score | | |
| --- | --- | --- | --- |
|  | Baseline DWRT score ≥4 | Baseline DWRT score <4 | P for interaction |
| Sarcopenia |  |  | 0.003 |
| No | Ref. (0) | Ref. (0) |  |
| Yes | -0.13 (-0.21 to -0.05)^**^ | 0.12 (-0.10 to 0.34) |  |
| Sarcopenic obesity (Weight) |  |  | 0.02 |
| Without sarcopenia and obesity | Ref. (0) | Ref. (0) |  |
| Only with sarcopenia | -0.16 (-0.26 to -0.06)^**^ | 0.16 (-0.09 to 0.41) |  |
| Only with obesity | -0.04 (-0.17 to 0.08) | 0.08 (-0.31 to 0.48) |  |
| Sarcopenic obesity | -0.13 (-0.24 to -0.02)^*^ | 0.11 (-0.16 to 0.39) |  |
| Sarcopenic obesity (BMI) |  |  | 0.02 |
| Without sarcopenia and obesity | Ref. (0) | Ref. (0) |  |
| Only with sarcopenia | -0.14 (-0.24 to -0.04)^**^ | 0.20 (-0.06 to 0.46) |  |
| Only with obesity | -0.03 (-0.16 to 0.09) | 0.22 (-0.16 to 0.61) |  |
| Sarcopenic obesity | -0.14 (-0.25 to -0.03)^*^ | 0.18 (-0.09 to 0.45) |  |
| Sarcopenic obesity (WC) |  |  | 0.02 |
| Without sarcopenia and obesity | Ref. (0) | Ref. (0) |  |
| Only with sarcopenia | -0.14 (-0.24 to -0.05)^**^ | 0.03 (-0.22 to 0.29) |  |
| Only with obesity | -0.03 (-0.16 to 0.11) | -0.17 (-0.58 to 0.23) |  |
| Sarcopenic obesity | -0.13 (-0.25 to -0.01)^*^ | 0.15 (-0.13 to 0.43) |  |
| Sarcopenic obesity (HC) |  |  | 0.02 |
| Without sarcopenia and obesity | Ref. (0) | Ref. (0) |  |
| Only with sarcopenia | -0.15 (-0.25 to -0.05)^**^ | 0.17 (-0.08 to 0.42) |  |
| Only with obesity | -0.06 (-0.19 to 0.06) | 0.09 (-0.33 to 0.51) |  |
| Sarcopenic obesity | -0.15 (-0.26 to -0.03)^*^ | 0.08 (-0.20 to 0.36) |  |
| Sarcopenic obesity (WHR) |  |  | 0.03 |
| Without sarcopenia and obesity | Ref. (0) | Ref. (0) |  |
| Only with sarcopenia | -0.17 (-0.31 to -0.03)^*^ | 0.03 (-0.35 to 0.42) |  |
| Only with obesity | -0.11 (-0.23 to 0.01) | -0.11 (-0.50 to 0.28) |  |
| Sarcopenic obesity | -0.22 (-0.34 to -0.10)^***^ | 0.05 (-0.30 to 0.40) |  |
| Sarcopenic obesity (WHtR) |  |  | 0.03 |
| Without sarcopenia and obesity | Ref. (0) | Ref. (0) |  |
| Only with sarcopenia | -0.14 (-0.24 to -0.03)^**^ | 0.07 (-0.22 to 0.37) |  |
| Only with obesity | -0.05 (-0.17 to 0.07) | -0.10 (-0.46 to 0.26) |  |
| Sarcopenic obesity | -0.17 (-0.28 to -0.05)^**^ | 0.07 (-0.21 to 0.36) |  |
| Sarcopenic obesity (LAP) |  |  | 0.02 |
| Without sarcopenia and obesity | Ref. (0) | Ref. (0) |  |
| Only with sarcopenia | -0.12 (-0.22 to -0.02)^*^ | 0.13 (-0.13 to 0.39) |  |
| Only with obesity | 0.03 (-0.10 to 0.15) | -0.19 (-0.57 to 0.19) |  |
| Sarcopenic obesity | -0.13 (-0.25 to -0.01)^*^ | -0.03 (-0.30 to 0.24) |  |
| Sarcopenic obesity (ABSI) |  |  | 0.01 |
| Without sarcopenia and obesity | Ref. (0) | Ref. (0) |  |
| Only with sarcopenia | -0.13 (-0.23 to -0.03)^**^ | 0.01 (-0.25 to 0.28) |  |
| Only with obesity | -0.07 (-0.20 to 0.06) | -0.25 (-0.62 to 0.12) |  |
| Sarcopenic obesity | -0.20 (-0.32 to -0.08)^***^ | 0.07 (-0.22 to 0.35) |  |
| Sarcopenic obesity (VAI) |  |  | 0.02 |
| Without sarcopenia and obesity | Ref. (0) | Ref. (0) |  |
| Only with sarcopenia | -0.11 (-0.21 to -0.01)^*^ | 0.11 (-0.15 to 0.37) |  |
| Only with obesity | 0.09 (-0.03 to 0.22) | -0.16 (-0.53 to 0.21) |  |
| Sarcopenic obesity | -0.09 (-0.21 to 0.02) | -0.01 (-0.29 to 0.27) |  |
| Sarcopenic obesity (CVAI) |  |  | 0.03 |
| Without sarcopenia and obesity | Ref. (0) | Ref. (0) |  |
| Only with sarcopenia | -0.14 (-0.23 to -0.04)^**^ | 0.11 (-0.15 to 0.37) |  |
| Only with obesity | 0.03 (-0.11 to 0.16) | -0.16 (-0.55 to 0.23) |  |
| Sarcopenic obesity | -0.10 (-0.22 to 0.02) | 0.02 (-0.26 to 0.29) |  |
| Sarcopenic obesity (BRI) |  |  | 0.02 |
| Without sarcopenia and obesity | Ref. (0) | Ref. (0) |  |
| Only with sarcopenia | -0.15 (-0.24 to -0.05)^**^ | 0.01 (-0.25 to 0.28) |  |
| Only with obesity | -0.05 (-0.19 to 0.08) | -0.26 (-0.64 to 0.11) |  |
| Sarcopenic obesity | -0.15 (-0.26 to -0.03)^*^ | 0.06 (-0.21 to 0.33) |  |
| Sarcopenic obesity (Conicity index) |  |  | 0.02 |
| Without sarcopenia and obesity | Ref. (0) | Ref. (0) |  |
| Only with sarcopenia | -0.15 (-0.25 to -0.06)^**^ | 0.08 (-0.19 to 0.35) |  |
| Only with obesity | -0.08 (-0.21 to 0.06) | -0.11 (-0.48 to 0.25) |  |
| Sarcopenic obesity | -0.16 (-0.28 to -0.04)^*^ | 0.07 (-0.23 to 0.36) |  |
| Sarcopenic obesity (BAI) |  |  | 0.02 |
| Without sarcopenia and obesity | Ref. (0) | Ref. (0) |  |
| Only with sarcopenia | -0.14 (-0.24 to -0.04)^**^ | 0.21 (-0.06 to 0.47) |  |
| Only with obesity | -0.001 (-0.13 to 0.13) | 0.17 (-0.21 to 0.55) |  |
| Sarcopenic obesity | -0.12 (-0.23 to -0.002)^*^ | 0.13 (-0.15 to 0.41) |  |
| Sarcopenic obesity (CMI) |  |  | 0.01 |
| Without sarcopenia and obesity | Ref. (0) | Ref. (0) |  |
| Only with sarcopenia | -0.10 (-0.20 to -0.01)^*^ | 0.11 (-0.15 to 0.37) |  |
| Only with obesity | 0.09 (-0.04 to 0.22) | -0.22 (-0.60 to 0.15) |  |
| Sarcopenic obesity | -0.11 (-0.22 to 0.01) | -0.06 (-0.33 to 0.22) |  |
| Sarcopenic obesity (BSA) |  |  | 0.02 |
| Without sarcopenia and obesity | Ref. (0) | Ref. (0) |  |
| Only with sarcopenia | -0.18 (-0.28 to -0.08)^***^ | 0.18 (-0.07 to 0.43) |  |
| Only with obesity | -0.05 (-0.17 to 0.07) | 0.24 (-0.17 to 0.64) |  |
| Sarcopenic obesity | -0.10 (-0.22 to 0.02) | 0.17 (-0.11 to 0.45) |  |
| Sarcopenic obesity (WHHR) |  |  | 0.006 |
| Without sarcopenia and obesity | Ref. (0) | Ref. (0) |  |
| Only with sarcopenia | -0.10 (-0.20 to -0.01)^*^ | 0.10 (-0.16 to 0.37) |  |
| Only with obesity | -0.01 (-0.15 to 0.13) | 0.00 (-0.38 to 0.38) |  |
| Sarcopenic obesity | -0.21 (-0.32 to -0.09)^***^ | 0.14 (-0.14 to 0.41) |  |
| Sarcopenic obesity (PFM) |  |  | 0.02 |
| Without sarcopenia and obesity | Ref. (0) | Ref. (0) |  |
| Only with sarcopenia | -0.13 (-0.23 to -0.03)^*^ | 0.18 (-0.08 to 0.44) |  |
| Only with obesity | 0.00 (-0.13 to 0.12) | 0.09 (-0.30 to 0.48) |  |
| Sarcopenic obesity | -0.14 (-0.25 to -0.03)^*^ | 0.10 (-0.18 to 0.37) |  |
| Sarcopenic obesity (PLM) |  |  | 0.02 |
| Without sarcopenia and obesity | Ref. (0) | Ref. (0) |  |
| Only with sarcopenia | -0.17 (-0.27 to -0.07)^**^ | 0.16 (-0.09 to 0.41) |  |
| Only with obesity | -0.04 (-0.16 to 0.08) | 0.14 (-0.25 to 0.53) |  |
| Sarcopenic obesity | -0.10 (-0.22 to 0.01) | 0.16 (-0.13 to 0.45) |  |
| Sarcopenic obesity (PPF) |  |  | 0.03 |
| Without sarcopenia and obesity | Ref. (0) | Ref. (0) |  |
| Only with sarcopenia | -0.12 (-0.22 to -0.03)^*^ | 0.10 (-0.16 to 0.36) |  |
| Only with obesity | -0.02 (-0.16 to 0.11) | -0.11 (-0.50 to 0.28) |  |
| Sarcopenic obesity | -0.17 (-0.28 to -0.05)^**^ | 0.08 (-0.19 to 0.34) |  |
| Sarcopenic obesity (CUN-BAE) |  |  | 0.02 |
| Without sarcopenia and obesity | Ref. (0) | Ref. (0) |  |
| Only with sarcopenia | -0.13 (-0.23 to -0.03)^**^ | 0.19 (-0.07 to 0.45) |  |
| Only with obesity | -0.001 (-0.14 to 0.13) | 0.20 (-0.20 to 0.60) |  |
| Sarcopenic obesity | -0.14 (-0.26 to -0.03)^*^ | 0.17 (-0.10 to 0.45) |  |

ABSI: a body shape index; BAI: body adiposity index; BMI: body mass index; BRI: body roundness index; BSA: body surface area; CI: confidence interval; CMI: cardiometabolic index; CUN-BAE: Clínica Universidad de Navarra-Body Adiposity Estimator; CVAI: Chinese visceral adiposity index; DWRT: Delayed Word Recall Test; HC, hip circumference; LAP: lipid accumulation product; PFM: predicted fat mass; PLM: predicted lean mass; PPF: predicated percent fat; Ref, reference; VAI: visceral adiposity index; WC: waist circumference; WHHR: waist-to-hip-to-height ratio; WHR: waist-to-hip ratio; WHtR: waist-to-height ratio

Note: Adjusted for sex, age, education, occupation, personal income, physical activity, drinking, smoking and self-rated health

^*^P <0.05, ^**^P <0.01, ^***^P <0.001

Table S12. Associations of baseline sarcopenia and sarcopenic obesity with memory function at follow-up by sex.

|  | Adjusted mean differences β (95% CI) in follow-up DWRT score | | |
| --- | --- | --- | --- |
|  | Women | Men | P for interaction |
| Sarcopenia |  |  | 0.08 |
| No | Ref. (0) | Ref. (0) |  |
| Yes | -0.04 (-0.15 to 0.08) | -0.29 (-0.49 to -0.09)^**^ |  |
| Sarcopenic obesity (Weight) |  |  | 0.02 |
| Without sarcopenia and obesity | Ref. (0) | Ref. (0) |  |
| Only with sarcopenia | -0.04 (-0.18 to 0.10) | -0.52 (-0.77 to -0.28)^***^ |  |
| Only with obesity | 0.01 (-0.17 to 0.18) | -0.21 (-0.49 to 0.07) |  |
| Sarcopenic obesity | -0.02 (-0.18 to 0.14) | -0.12 (-0.39 to 0.16) |  |
| Sarcopenic obesity (BMI) |  |  | 0.01 |
| Without sarcopenia and obesity | Ref. (0) | Ref. (0) |  |
| Only with sarcopenia | -0.01 (-0.15 to 0.13) | -0.48 (-0.72 to -0.24)^***^ |  |
| Only with obesity | 0.09 (-0.08 to 0.27) | -0.32 (-0.61 to -0.03)^*^ |  |
| Sarcopenic obesity | -0.01 (-0.17 to 0.15) | -0.24 (-0.52 to 0.03) |  |
| Sarcopenic obesity (WC) |  |  | 0.05 |
| Without sarcopenia and obesity | Ref. (0) | Ref. (0) |  |
| Only with sarcopenia | -0.0003 (-0.14 to 0.14) | -0.36 (-0.58 to -0.15)^***^ |  |
| Only with obesity | -0.12 (-0.30 to 0.06) | -0.30 (-0.69 to 0.09) |  |
| Sarcopenic obesity | -0.21 (-0.38 to -0.05)^*^ | -0.19 (-0.53 to 0.16) |  |
| Sarcopenic obesity (HC) |  |  | 0.21 |
| Without sarcopenia and obesity | Ref. (0) | Ref. (0) |  |
| Only with sarcopenia | -0.05 (-0.19 to 0.08) | -0.40 (-0.63 to -0.16)^***^ |  |
| Only with obesity | 0.03 (-0.15 to 0.21) | -0.13 (-0.42 to 0.17) |  |
| Sarcopenic obesity | 0.02 (-0.14 to 0.19) | -0.18 (-0.47 to 0.11) |  |
| Sarcopenic obesity (WHR) |  |  | 0.049 |
| Without sarcopenia and obesity | Ref. (0) | Ref. (0) |  |
| Only with sarcopenia | 0.04 (-0.17 to 0.26) | -0.45 (-0.72 to -0.18)^**^ |  |
| Only with obesity | -0.06 (-0.24 to 0.12) | -0.19 (-0.46 to 0.09) |  |
| Sarcopenic obesity | -0.13 (-0.31 to 0.05) | -0.31 (-0.57 to -0.05)^*^ |  |
| Sarcopenic obesity (WHtR) |  |  | 0.03 |
| Without sarcopenia and obesity | Ref. (0) | Ref. (0) |  |
| Only with sarcopenia | 0.02 (-0.13 to 0.16) | -0.41 (-0.67 to -0.15)^**^ |  |
| Only with obesity | -0.13 (-0.30 to 0.04) | -0.20 (-0.47 to 0.08) |  |
| Sarcopenic obesity | -0.22 (-0.38 to -0.06)^**^ | -0.33 (-0.60 to -0.07)^*^ |  |
| Sarcopenic obesity (LAP) |  |  | 0.002 |
| Without sarcopenia and obesity | Ref. (0) | Ref. (0) |  |
| Only with sarcopenia | -0.02 (-0.15 to 0.12) | -0.47 (-0.71 to -0.24)^***^ |  |
| Only with obesity | -0.18 (-0.36 to 0.01) | -0.22 (-0.50 to 0.07) |  |
| Sarcopenic obesity | -0.24 (-0.40 to -0.08)^**^ | -0.17 (-0.45 to 0.11) |  |
| Sarcopenic obesity (ABSI) |  |  | 0.14 |
| Without sarcopenia and obesity | Ref. (0) | Ref. (0) |  |
| Only with sarcopenia | -0.002 (-0.13 to 0.13) | -0.20 (-0.43 to 0.03) |  |
| Only with obesity | -0.07 (-0.26 to 0.12) | 0.19 (-0.11 to 0.48) |  |
| Sarcopenic obesity | -0.19 (-0.36 to -0.02)^*^ | -0.32 (-0.62 to -0.03)^*^ |  |
| Sarcopenic obesity (VAI) |  |  | 0.10 |
| Without sarcopenia and obesity | Ref. (0) | Ref. (0) |  |
| Only with sarcopenia | -0.02 (-0.16 to 0.12) | -0.40 (-0.64 to -0.16)^***^ |  |
| Only with obesity | -0.11 (-0.29 to 0.06) | -0.22 (-0.51 to 0.07) |  |
| Sarcopenic obesity | -0.19 (-0.36 to -0.03)^*^ | -0.29 (-0.57 to -0.02)^*^ |  |
| Sarcopenic obesity (CVAI) |  |  | 0.06 |
| Without sarcopenia and obesity | Ref. (0) | Ref. (0) |  |
| Only with sarcopenia | 0.03 (-0.11 to 0.16) | -0.35 (-0.59 to -0.11)^**^ |  |
| Only with obesity | -0.04 (-0.23 to 0.16) | -0.13 (-0.42 to 0.17) |  |
| Sarcopenic obesity | -0.21 (-0.38 to -0.04)^*^ | -0.29 (-0.57 to -0.02)^*^ |  |
| Sarcopenic obesity (BRI) |  |  | 0.01 |
| Without sarcopenia and obesity | Ref. (0) | Ref. (0) |  |
| Only with sarcopenia | 0.01 (-0.12 to 0.15) | -0.44 (-0.68 to -0.20)^***^ |  |
| Only with obesity | -0.11 (-0.30 to 0.09) | -0.37 (-0.66 to -0.07)^*^ |  |
| Sarcopenic obesity | -0.23 (-0.39 to -0.07)^**^ | -0.34 (-0.61 to -0.06)^*^ |  |
| Sarcopenic obesity (Conicity index) |  |  | 0.046 |
| Without sarcopenia and obesity | Ref. (0) | Ref. (0) |  |
| Only with sarcopenia | 0.001 (-0.13 to 0.13) | -0.12 (-0.34 to 0.10) |  |
| Only with obesity | 0.15 (-0.04 to 0.34) | 0.24 (-0.07 to 0.54) |  |
| Sarcopenic obesity | 0.01 (-0.16 to 0.18) | -0.56 (-0.86 to -0.25)^***^ |  |
| Sarcopenic obesity (BAI) |  |  | 0.045 |
| Without sarcopenia and obesity | Ref. (0) | Ref. (0) |  |
| Only with sarcopenia | 0.01 (-0.12 to 0.15) | -0.41 (-0.65 to -0.17)^***^ |  |
| Only with obesity | 0.04 (-0.15 to 0.22) | -0.24 (-0.54 to 0.05) |  |
| Sarcopenic obesity | -0.10 (-0.26 to 0.06) | -0.29 (-0.56 to -0.01)^*^ |  |
| Sarcopenic obesity (CMI) |  |  | 0.04 |
| Without sarcopenia and obesity | Ref. (0) | Ref. (0) |  |
| Only with sarcopenia | -0.01 (-0.15 to 0.12) | -0.42 (-0.66 to -0.18)^***^ |  |
| Only with obesity | -0.16 (-0.34 to 0.02) | -0.25 (-0.55 to 0.04) |  |
| Sarcopenic obesity | -0.24 (-0.40 to -0.08)^**^ | -0.29 (-0.56 to -0.02)^*^ |  |
| Sarcopenic obesity (BSA) |  |  | 0.14 |
| Without sarcopenia and obesity | Ref. (0) | Ref. (0) |  |
| Only with sarcopenia | -0.08 (-0.22 to 0.06) | -0.46 (-0.70 to -0.22)^***^ |  |
| Only with obesity | 0.01 (-0.15 to 0.18) | -0.08 (-0.36 to 0.19) |  |
| Sarcopenic obesity | 0.05 (-0.11 to 0.21) | -0.08 (-0.37 to 0.20) |  |
| Sarcopenic obesity (WHHR) |  |  | 0.19 |
| Without sarcopenia and obesity | Ref. (0) | Ref. (0) |  |
| Only with sarcopenia | 0.001 (-0.13 to 0.14) | -0.30 (-0.53 to -0.07)^*^ |  |
| Only with obesity | -0.17 (-0.37 to 0.03) | -0.19 (-0.50 to 0.12) |  |
| Sarcopenic obesity | -0.26 (-0.42 to -0.09)^**^ | -0.43 (-0.71 to -0.15)^**^ |  |
| Sarcopenic obesity (PFM) |  |  | 0.06 |
| Without sarcopenia and obesity | Ref. (0) | Ref. (0) |  |
| Only with sarcopenia | -0.03 (-0.17 to 0.11) | -0.45 (-0.69 to -0.21)^***^ |  |
| Only with obesity | -0.001 (-0.18 to 0.17) | -0.22 (-0.51 to 0.06) |  |
| Sarcopenic obesity | -0.06 (-0.22 to 0.10) | -0.23 (-0.50 to 0.04) |  |
| Sarcopenic obesity (PLM) |  |  | 0.13 |
| Without sarcopenia and obesity | Ref. (0) | Ref. (0) |  |
| Only with sarcopenia | -0.08 (-0.22 to 0.06) | -0.47 (-0.72 to -0.23)^***^ |  |
| Only with obesity | -0.02 (-0.19 to 0.15) | -0.18 (-0.46 to 0.09) |  |
| Sarcopenic obesity | 0.01 (-0.15 to 0.18) | -0.17 (-0.45 to 0.12) |  |
| Sarcopenic obesity (PPF) |  |  | 0.10 |
| Without sarcopenia and obesity | Ref. (0) | Ref. (0) |  |
| Only with sarcopenia | -0.02 (-0.16 to 0.11) | -0.38 (-0.62 to -0.15)^**^ |  |
| Only with obesity | -0.03 (-0.23 to 0.16) | -0.32 (-0.62 to -0.02)^*^ |  |
| Sarcopenic obesity | -0.09 (-0.25 to 0.07) | -0.39 (-0.67 to -0.12)^**^ |  |
| Sarcopenic obesity (CUN-BAE) |  |  | 0.03 |
| Without sarcopenia and obesity | Ref. (0) | Ref. (0) |  |
| Only with sarcopenia | -0.01 (-0.15 to 0.13) | -0.45 (-0.69 to -0.21)^***^ |  |
| Only with obesity | -0.03 (-0.22 to 0.16) | -0.32 (-0.62 to -0.03)^*^ |  |
| Sarcopenic obesity | -0.11 (-0.27 to 0.05) | -0.29 (-0.56 to -0.01)^*^ |  |

ABSI: a body shape index; BAI: body adiposity index; BMI: body mass index; BRI: body roundness index; BSA: body surface area; CI: confidence interval; CMI: cardiometabolic index; CUN-BAE: Clínica Universidad de Navarra-Body Adiposity Estimator; CVAI: Chinese visceral adiposity index; DWRT: Delayed Word Recall Test; HC, hip circumference; LAP: lipid accumulation product; PFM: predicted fat mass; PLM: predicted lean mass; PPF: predicated percent fat; Ref, reference; VAI: visceral adiposity index; WC: waist circumference; WHHR: waist-to-hip-to-height ratio; WHR: waist-to-hip ratio; WHtR: waist-to-height ratio

Note: Adjusted for sex, age, education, occupation, personal income, physical activity, drinking, smoking, self-rated health and baseline DWRT score

^*^P <0.05, ^**^P <0.01, ^***^P <0.001

Table S13. Associations of baseline sarcopenia and sarcopenic obesity with memory function at follow-up by age.

|  | Adjusted mean differences β (95% CI) in follow-up DWRT score | | |
| --- | --- | --- | --- |
|  | ≤60 | >60 | P for interaction |
| Sarcopenia |  |  | 0.05 |
| No | Ref. (0) | Ref. (0) |  |
| Yes | -0.06 (-0.18 to 0.05) | -0.16 (-0.35 to 0.02) |  |
| Sarcopenic obesity (Weight) |  |  | 0.32 |
| Without sarcopenia and obesity | Ref. (0) | Ref. (0) |  |
| Only with sarcopenia | -0.12 (-0.27 to 0.02) | -0.23 (-0.45 to -0.01)^*^ |  |
| Only with obesity | -0.04 (-0.21 to 0.12) | -0.12 (-0.42 to 0.18) |  |
| Sarcopenic obesity | -0.001 (-0.17 to 0.16) | -0.15 (-0.39 to 0.10) |  |
| Sarcopenic obesity (BMI) |  |  | 0.14 |
| Without sarcopenia and obesity | Ref. (0) | Ref. (0) |  |
| Only with sarcopenia | -0.08 (-0.22 to 0.07) | -0.23 (-0.45 to -0.01)^*^ |  |
| Only with obesity | 0.02 (-0.15 to 0.20) | -0.20 (-0.50 to 0.11) |  |
| Sarcopenic obesity | -0.02 (-0.19 to 0.15) | -0.22 (-0.46 to 0.01) |  |
| Sarcopenic obesity (WC) |  |  | 0.13 |
| Without sarcopenia and obesity | Ref. (0) | Ref. (0) |  |
| Only with sarcopenia | -0.09 (-0.22 to 0.05) | -0.15 (-0.37 to 0.06) |  |
| Only with obesity | -0.19 (-0.38 to -0.01)^*^ | -0.21 (-0.54 to 0.12) |  |
| Sarcopenic obesity | -0.17 (-0.35 to 0.02) | -0.36 (-0.61 to -0.11)^**^ |  |
| Sarcopenic obesity (HC) |  |  | 0.20 |
| Without sarcopenia and obesity | Ref. (0) | Ref. (0) |  |
| Only with sarcopenia | -0.10 (-0.25 to 0.04) | -0.19 (-0.40 to 0.02) |  |
| Only with obesity | -0.06 (-0.24 to 0.11) | 0.06 (-0.25 to 0.37) |  |
| Sarcopenic obesity | -0.04 (-0.21 to 0.14) | -0.05 (-0.30 to 0.20) |  |
| Sarcopenic obesity (WHR) |  |  | 0.11 |
| Without sarcopenia and obesity | Ref. (0) | Ref. (0) |  |
| Only with sarcopenia | -0.13 (-0.33 to 0.06) | -0.23 (-0.54 to 0.09) |  |
| Only with obesity | -0.12 (-0.29 to 0.04) | -0.22 (-0.53 to 0.08) |  |
| Sarcopenic obesity | -0.15 (-0.32 to 0.02) | -0.34 (-0.62 to -0.06)^*^ |  |
| Sarcopenic obesity (WHtR) |  |  | 0.10 |
| Without sarcopenia and obesity | Ref. (0) | Ref. (0) |  |
| Only with sarcopenia | -0.09 (-0.24 to 0.06) | -0.14 (-0.39 to 0.11) |  |
| Only with obesity | -0.16 (-0.33 to 0.005) | -0.23 (-0.52 to 0.05) |  |
| Sarcopenic obesity | -0.16 (-0.33 to 0.01) | -0.37 (-0.61 to -0.13)^**^ |  |
| Sarcopenic obesity (LAP) |  |  | 0.18 |
| Without sarcopenia and obesity | Ref. (0) | Ref. (0) |  |
| Only with sarcopenia | -0.10 (-0.24 to 0.04) | -0.17 (-0.39 to 0.05) |  |
| Only with obesity | -0.19 (-0.37 to -0.02)^*^ | -0.22 (-0.53 to 0.08) |  |
| Sarcopenic obesity | -0.15 (-0.33 to 0.03) | -0.34 (-0.58 to -0.10)^**^ |  |
| Sarcopenic obesity (ABSI) |  |  | 0.28 |
| Without sarcopenia and obesity | Ref. (0) | Ref. (0) |  |
| Only with sarcopenia | -0.05 (-0.18 to 0.09) | -0.06 (-0.28 to 0.16) |  |
| Only with obesity | -0.08 (-0.27 to 0.11) | 0.12 (-0.18 to 0.42) |  |
| Sarcopenic obesity | -0.20 (-0.39 to 0.001) | -0.22 (-0.47 to 0.02) |  |
| Sarcopenic obesity (VAI) |  |  | 0.20 |
| Without sarcopenia and obesity | Ref. (0) | Ref. (0) |  |
| Only with sarcopenia | -0.06 (-0.20 to 0.08) | -0.22 (-0.44 to 0.003) |  |
| Only with obesity | -0.12 (-0.29 to 0.05) | -0.28 (-0.58 to 0.02) |  |
| Sarcopenic obesity | -0.20 (-0.38 to -0.02)^*^ | -0.32 (-0.56 to -0.08)^**^ |  |
| Sarcopenic obesity (CVAI) |  |  | 0.19 |
| Without sarcopenia and obesity | Ref. (0) | Ref. (0) |  |
| Only with sarcopenia | -0.05 (-0.19 to 0.08) | -0.14 (-0.37 to 0.09) |  |
| Only with obesity | -0.07 (-0.27 to 0.13) | -0.18 (-0.47 to 0.11) |  |
| Sarcopenic obesity | -0.15 (-0.34 to 0.04) | -0.35 (-0.59 to -0.11)^**^ |  |
| Sarcopenic obesity (BRI) |  |  | 0.17 |
| Without sarcopenia and obesity | Ref. (0) | Ref. (0) |  |
| Only with sarcopenia | -0.08 (-0.21 to 0.06) | -0.18 (-0.40 to 0.05) |  |
| Only with obesity | -0.19 (-0.38 to 0.002) | -0.29 (-0.59 to 0.02) |  |
| Sarcopenic obesity | -0.18 (-0.36 to 0.004) | -0.37 (-0.61 to -0.14)^**^ |  |
| Sarcopenic obesity (Conicity index) |  |  | 0.42 |
| Without sarcopenia and obesity | Ref. (0) | Ref. (0) |  |
| Only with sarcopenia | -0.002 (-0.13 to 0.13) | -0.09 (-0.32 to 0.13) |  |
| Only with obesity | 0.13 (-0.07 to 0.32) | 0.21 (-0.09 to 0.51) |  |
| Sarcopenic obesity | -0.15 (-0.35 to 0.05) | -0.07 (-0.32 to 0.18) |  |
| Sarcopenic obesity (BAI) |  |  | 0.17 |
| Without sarcopenia and obesity | Ref. (0) | Ref. (0) |  |
| Only with sarcopenia | -0.06 (-0.19 to 0.08) | -0.19 (-0.41 to 0.03) |  |
| Only with obesity | -0.01 (-0.19 to 0.17) | -0.20 (-0.50 to 0.11) |  |
| Sarcopenic obesity | -0.08 (-0.26 to 0.10) | -0.27 (-0.51 to -0.04)^*^ |  |
| Sarcopenic obesity (CMI) |  |  | 0.17 |
| Without sarcopenia and obesity | Ref. (0) | Ref. (0) |  |
| Only with sarcopenia | -0.05 (-0.19 to 0.09) | -0.22 (-0.44 to -0.004)^*^ |  |
| Only with obesity | -0.15 (-0.33 to 0.02) | -0.33 (-0.63 to -0.03)^*^ |  |
| Sarcopenic obesity | -0.24 (-0.42 to -0.06)^**^ | -0.35 (-0.59 to -0.11)^**^ |  |
| Sarcopenic obesity (BSA) |  |  | 0.22 |
| Without sarcopenia and obesity | Ref. (0) | Ref. (0) |  |
| Only with sarcopenia | -0.16 (-0.31 to -0.01)^*^ | -0.18 (-0.39 to 0.04) |  |
| Only with obesity | -0.05 (-0.21 to 0.11) | 0.10 (-0.20 to 0.40) |  |
| Sarcopenic obesity | 0.04 (-0.13 to 0.21) | -0.04 (-0.29 to 0.21) |  |
| Sarcopenic obesity (WHHR) |  |  | 0.30 |
| Without sarcopenia and obesity | Ref. (0) | Ref. (0) |  |
| Only with sarcopenia | -0.06 (-0.19 to 0.07) | -0.11 (-0.34 to 0.11) |  |
| Only with obesity | -0.22 (-0.42 to -0.02)^*^ | -0.20 (-0.50 to 0.11) |  |
| Sarcopenic obesity | -0.25 (-0.44 to -0.05)^*^ | -0.37 (-0.60 to -0.13)^**^ |  |
| Sarcopenic obesity (PFM) |  |  | 0.26 |
| Without sarcopenia and obesity | Ref. (0) | Ref. (0) |  |
| Only with sarcopenia | -0.11 (-0.25 to 0.04) | -0.19 (-0.41 to 0.03) |  |
| Only with obesity | -0.08 (-0.25 to 0.09) | -0.09 (-0.39 to 0.21) |  |
| Sarcopenic obesity | -0.05 (-0.22 to 0.12) | -0.21 (-0.45 to 0.03) |  |
| Sarcopenic obesity (PLM) |  |  | 0.36 |
| Without sarcopenia and obesity | Ref. (0) | Ref. (0) |  |
| Only with sarcopenia | -0.14 (-0.29 to 0.01) | -0.21 (-0.42 to 0.003) |  |
| Only with obesity | -0.07 (-0.23 to 0.09) | -0.03 (-0.34 to 0.27) |  |
| Sarcopenic obesity | -0.01 (-0.18 to 0.15) | -0.09 (-0.35 to 0.16) |  |
| Sarcopenic obesity (PPF) |  |  | 0.045 |
| Without sarcopenia and obesity | Ref. (0) | Ref. (0) |  |
| Only with sarcopenia | -0.09 (-0.23 to 0.04) | -0.21 (-0.44 to 0.01) |  |
| Only with obesity | -0.08 (-0.27 to 0.11) | -0.31 (-0.61 to -0.01)^*^ |  |
| Sarcopenic obesity | -0.05 (-0.23 to 0.13) | -0.34 (-0.58 to -0.11)^**^ |  |
| Sarcopenic obesity (CUN-BAE) |  |  | 0.12 |
| Without sarcopenia and obesity | Ref. (0) | Ref. (0) |  |
| Only with sarcopenia | -0.09 (-0.23 to 0.04) | -0.22 (-0.45 to 0.01) |  |
| Only with obesity | -0.10 (-0.29 to 0.09) | -0.25 (-0.55 to 0.04) |  |
| Sarcopenic obesity | -0.07 (-0.25 to 0.11) | -0.30 (-0.54 to -0.07)^*^ |  |

ABSI: a body shape index; BAI: body adiposity index; BMI: body mass index; BRI: body roundness index; BSA: body surface area; CI: confidence interval; CMI: cardiometabolic index; CUN-BAE: Clínica Universidad de Navarra-Body Adiposity Estimator; CVAI: Chinese visceral adiposity index; DWRT: Delayed Word Recall Test; HC, hip circumference; LAP: lipid accumulation product; PFM: predicted fat mass; PLM: predicted lean mass; PPF: predicated percent fat; Ref, reference; VAI: visceral adiposity index; WC: waist circumference; WHHR: waist-to-hip-to-height ratio; WHR: waist-to-hip ratio; WHtR: waist-to-height ratio

Note: Adjusted for sex, age, education, occupation, personal income, physical activity, drinking, smoking, self-rated health and baseline DWRT score

^*^P <0.05, ^**^P <0.01, ^***^P <0.001

Table S14. Associations of baseline sarcopenia and sarcopenic obesity with memory function at follow-up by baseline memory status.

|  | Adjusted mean differences β (95% CI) in follow-up DWRT score | | |
| --- | --- | --- | --- |
|  | Baseline DWRT score ≥4 | Baseline DWRT score <4 | P for interaction |
| Sarcopenia |  |  | 0.22 |
| No | Ref. (0) | Ref. (0) |  |
| Yes | -0.11 (-0.21 to -0.01)^*^ | -0.07 (-0.50 to 0.36) |  |
| Sarcopenic obesity (Weight) |  |  | 0.70 |
| Without sarcopenia and obesity | Ref. (0) | Ref. (0) |  |
| Only with sarcopenia | -0.17 (-0.29 to -0.05)^**^ | -0.07 (-0.58 to 0.44) |  |
| Only with obesity | -0.05 (-0.20 to 0.09) | -0.08 (-0.85 to 0.68) |  |
| Sarcopenic obesity | -0.05 (-0.19 to 0.09) | -0.14 (-0.70 to 0.42) |  |
| Sarcopenic obesity (BMI) |  |  | 0.44 |
| Without sarcopenia and obesity | Ref. (0) | Ref. (0) |  |
| Only with sarcopenia | -0.13 (-0.25 to -0.01)^*^ | 0.04 (-0.50 to 0.58) |  |
| Only with obesity | -0.04 (-0.19 to 0.12) | 0.45 (-0.30 to 1.19) |  |
| Sarcopenic obesity | -0.10 (-0.24 to 0.04) | 0.12 (-0.43 to 0.67) |  |
| Sarcopenic obesity (WC) |  |  | 0.48 |
| Without sarcopenia and obesity | Ref. (0) | Ref. (0) |  |
| Only with sarcopenia | -0.12 (-0.23 to 0.002) | 0.03 (-0.47 to 0.53) |  |
| Only with obesity | -0.19 (-0.36 to -0.03)^*^ | 0.13 (-0.68 to 0.94) |  |
| Sarcopenic obesity | -0.25 (-0.40 to -0.10)^***^ | -0.19 (-0.76 to 0.39) |  |
| Sarcopenic obesity (HC) |  |  | 0.22 |
| Without sarcopenia and obesity | Ref. (0) | Ref. (0) |  |
| Only with sarcopenia | -0.15 (-0.27 to -0.03)^*^ | 0.05 (-0.45 to 0.54) |  |
| Only with obesity | -0.05 (-0.20 to 0.11) | 0.58 (-0.19 to 1.36) |  |
| Sarcopenic obesity | -0.06 (-0.20 to 0.09) | 0.14 (-0.44 to 0.72) |  |
| Sarcopenic obesity (WHR) |  |  | 0.58 |
| Without sarcopenia and obesity | Ref. (0) | Ref. (0) |  |
| Only with sarcopenia | -0.16 (-0.33 to 0.02) | -0.11 (-0.90 to 0.68) |  |
| Only with obesity | -0.13 (-0.28 to 0.02) | -0.19 (-0.94 to 0.56) |  |
| Sarcopenic obesity | -0.21 (-0.36 to -0.06)^**^ | -0.23 (-0.92 to 0.46) |  |
| Sarcopenic obesity (WHtR) |  |  | 0.34 |
| Without sarcopenia and obesity | Ref. (0) | Ref. (0) |  |
| Only with sarcopenia | -0.10 (-0.23 to 0.03) | 0.11 (-0.48 to 0.69) |  |
| Only with obesity | -0.16 (-0.31 to -0.02)^*^ | -0.09 (-0.79 to 0.61) |  |
| Sarcopenic obesity | -0.25 (-0.39 to -0.11)^***^ | -0.29 (-0.87 to 0.28) |  |
| Sarcopenic obesity (LAP) |  |  | 0.65 |
| Without sarcopenia and obesity | Ref. (0) | Ref. (0) |  |
| Only with sarcopenia | -0.13 (-0.25 to -0.01)^*^ | -0.02 (-0.54 to 0.50) |  |
| Only with obesity | -0.20 (-0.36 to -0.05)^*^ | -0.20 (-0.95 to 0.54) |  |
| Sarcopenic obesity | -0.24 (-0.39 to -0.10)^**^ | -0.31 (-0.87 to 0.24) |  |
| Sarcopenic obesity (ABSI) |  |  | 0.47 |
| Without sarcopenia and obesity | Ref. (0) | Ref. (0) |  |
| Only with sarcopenia | -0.06 (-0.18 to 0.06) | 0.03 (-0.49 to 0.55) |  |
| Only with obesity | -0.02 (-0.19 to 0.14) | 0.20 (-0.55 to 0.94) |  |
| Sarcopenic obesity | -0.25 (-0.40 to -0.09)^**^ | -0.08 (-0.66 to 0.50) |  |
| Sarcopenic obesity (VAI) |  |  | 0.65 |
| Without sarcopenia and obesity | Ref. (0) | Ref. (0) |  |
| Only with sarcopenia | -0.12 (-0.24 to 0.002) | -0.04 (-0.56 to 0.48) |  |
| Only with obesity | -0.16 (-0.32 to -0.01)^*^ | -0.15 (-0.87 to 0.57) |  |
| Sarcopenic obesity | -0.24 (-0.39 to -0.09)^**^ | -0.28 (-0.85 to 0.29) |  |
| Sarcopenic obesity (CVAI) |  |  | 0.21 |
| Without sarcopenia and obesity | Ref. (0) | Ref. (0) |  |
| Only with sarcopenia | -0.08 (-0.20 to 0.04) | 0.01 (-0.50 to 0.52) |  |
| Only with obesity | -0.07 (-0.24 to 0.10) | -0.28 (-1.04 to 0.48) |  |
| Sarcopenic obesity | -0.23 (-0.38 to -0.08)^**^ | -0.43 (-0.98 to 0.12) |  |
| Sarcopenic obesity (BRI) |  |  | 0.49 |
| Without sarcopenia and obesity | Ref. (0) | Ref. (0) |  |
| Only with sarcopenia | -0.11 (-0.22 to 0.01) | -0.06 (-0.58 to 0.46) |  |
| Only with obesity | -0.19 (-0.35 to -0.02)^*^ | -0.29 (-1.04 to 0.45) |  |
| Sarcopenic obesity | -0.26 (-0.41 to -0.12)^***^ | -0.30 (-0.85 to 0.24) |  |
| Sarcopenic obesity (Conicity index) |  |  | 0.45 |
| Without sarcopenia and obesity | Ref. (0) | Ref. (0) |  |
| Only with sarcopenia | -0.04 (-0.16 to 0.08) | -0.03 (-0.56 to 0.50) |  |
| Only with obesity | 0.17 (0.004 to 0.34)^*^ | 0.08 (-0.64 to 0.80) |  |
| Sarcopenic obesity | -0.13 (-0.28 to 0.03) | -0.07 (-0.69 to 0.55) |  |
| Sarcopenic obesity (BAI) |  |  | 0.16 |
| Without sarcopenia and obesity | Ref. (0) | Ref. (0) |  |
| Only with sarcopenia | -0.10 (-0.22 to 0.02) | 0.13 (-0.40 to 0.66) |  |
| Only with obesity | -0.08 (-0.24 to 0.08) | 0.64 (-0.10 to 1.37) |  |
| Sarcopenic obesity | -0.19 (-0.34 to -0.05)^**^ | 0.18 (-0.38 to 0.75) |  |
| Sarcopenic obesity (CMI) |  |  | 0.60 |
| Without sarcopenia and obesity | Ref. (0) | Ref. (0) |  |
| Only with sarcopenia | -0.12 (-0.24 to 0.003) | -0.04 (-0.56 to 0.48) |  |
| Only with obesity | -0.20 (-0.35 to -0.04)^*^ | -0.27 (-1.00 to 0.46) |  |
| Sarcopenic obesity | -0.26 (-0.41 to -0.12)^***^ | -0.39 (-0.95 to 0.18) |  |
| Sarcopenic obesity (BSA) |  |  | 0.55 |
| Without sarcopenia and obesity | Ref. (0) | Ref. (0) |  |
| Only with sarcopenia | -0.17 (-0.30 to -0.05)^**^ | -0.19 (-0.69 to 0.32) |  |
| Only with obesity | -0.01 (-0.15 to 0.14) | -0.18 (-0.95 to 0.59) |  |
| Sarcopenic obesity | 0.001 (-0.14 to 0.15) | 0.001 (-0.56 to 0.57) |  |
| Sarcopenic obesity (WHHR) |  |  | 0.02 |
| Without sarcopenia and obesity | Ref. (0) | Ref. (0) |  |
| Only with sarcopenia | -0.09 (-0.21 to 0.03) | 0.07 (-0.45 to 0.59) |  |
| Only with obesity | -0.17 (-0.34 to 0.004) | -0.33 (-1.08 to 0.42) |  |
| Sarcopenic obesity | -0.28 (-0.42 to -0.13)^***^ | -0.51 (-1.06 to 0.04) |  |
| Sarcopenic obesity (PFM) |  |  | 0.54 |
| Without sarcopenia and obesity | Ref. (0) | Ref. (0) |  |
| Only with sarcopenia | -0.13 (-0.26 to -0.01)^*^ | -0.13 (-0.66 to 0.40) |  |
| Only with obesity | -0.06 (-0.21 to 0.09) | -0.26 (-1.01 to 0.49) |  |
| Sarcopenic obesity | -0.11 (-0.26 to 0.03) | -0.19 (-0.75 to 0.37) |  |
| Sarcopenic obesity (PLM) |  |  | 0.29 |
| Without sarcopenia and obesity | Ref. (0) | Ref. (0) |  |
| Only with sarcopenia | -0.17 (-0.30 to -0.05)^**^ | -0.27 (-0.78 to 0.24) |  |
| Only with obesity | -0.04 (-0.19 to 0.11) | -0.62 (-1.37 to 0.13) |  |
| Sarcopenic obesity | -0.03 (-0.18 to 0.11) | -0.20 (-0.78 to 0.38) |  |
| Sarcopenic obesity (PPF) |  |  | 0.50 |
| Without sarcopenia and obesity | Ref. (0) | Ref. (0) |  |
| Only with sarcopenia | -0.12 (-0.24 to -0.005)^*^ | 0.06 (-0.46 to 0.58) |  |
| Only with obesity | -0.14 (-0.31 to 0.02) | 0.28 (-0.49 to 1.06) |  |
| Sarcopenic obesity | -0.19 (-0.33 to -0.04)^*^ | -0.05 (-0.59 to 0.49) |  |
| Sarcopenic obesity (CUN-BAE) |  |  | 0.43 |
| Without sarcopenia and obesity | Ref. (0) | Ref. (0) |  |
| Only with sarcopenia | -0.13 (-0.25 to -0.01)^*^ | 0.15 (-0.37 to 0.66) |  |
| Only with obesity | -0.14 (-0.30 to 0.02) | 0.68 (-0.13 to 1.49) |  |
| Sarcopenic obesity | -0.18 (-0.32 to -0.03)^*^ | 0.09 (-0.46 to 0.63) |  |

ABSI: a body shape index; BAI: body adiposity index; BMI: body mass index; BRI: body roundness index; BSA: body surface area; CI: confidence interval; CMI: cardiometabolic index; CUN-BAE: Clínica Universidad de Navarra-Body Adiposity Estimator; CVAI: Chinese visceral adiposity index; DWRT: Delayed Word Recall Test; HC, hip circumference; LAP: lipid accumulation product; PFM: predicted fat mass; PLM: predicted lean mass; PPF: predicated percent fat; Ref, reference; VAI: visceral adiposity index; WC: waist circumference; WHHR: waist-to-hip-to-height ratio; WHR: waist-to-hip ratio; WHtR: waist-to-height ratio

Note: Adjusted for sex, age, education, occupation, personal income, physical activity, drinking, smoking, self-rated health and baseline DWRT score

^*^P <0.05, ^**^P <0.01, ^***^P <0.001

Table S15. Associations of baseline sarcopenia and sarcopenic obesity with annual change of memory function by sex.

|  | Adjusted mean differences β (95% CI) in annual change of DWRT score | | |
| --- | --- | --- | --- |
|  | Women | Men | P for interaction |
| Sarcopenia |  |  | 0.09 |
| No | Ref. (0) | Ref. (0) |  |
| Yes | -0.01 (-0.05 to 0.02) | -0.08 (-0.14 to -0.02)^**^ |  |
| Sarcopenic obesity (Weight) |  |  | 0.02 |
| Without sarcopenia and obesity | Ref. (0) | Ref. (0) |  |
| Only with sarcopenia | -0.02 (-0.06 to 0.02) | -0.15 (-0.23 to -0.08)^***^ |  |
| Only with obesity | -0.01 (-0.06 to 0.05) | -0.07 (-0.15 to 0.01) |  |
| Sarcopenic obesity | -0.01 (-0.06 to 0.04) | -0.03 (-0.11 to 0.05) |  |
| Sarcopenic obesity (BMI) |  |  | 0.02 |
| Without sarcopenia and obesity | Ref. (0) | Ref. (0) |  |
| Only with sarcopenia | -0.01 (-0.05 to 0.03) | -0.14 (-0.21 to -0.07)^***^ |  |
| Only with obesity | 0.02 (-0.03 to 0.07) | -0.10 (-0.18 to -0.01)^*^ |  |
| Sarcopenic obesity | -0.001 (-0.05 to 0.04) | -0.07 (-0.15 to 0.01) |  |
| Sarcopenic obesity (WC) |  |  | 0.12 |
| Without sarcopenia and obesity | Ref. (0) | Ref. (0) |  |
| Only with sarcopenia | -0.01 (-0.05 to 0.03) | -0.10 (-0.16 to -0.04)^**^ |  |
| Only with obesity | -0.04 (-0.10 to 0.01) | -0.07 (-0.19 to 0.04) |  |
| Sarcopenic obesity | -0.06 (-0.11 to -0.02)^**^ | -0.06 (-0.16 to 0.05) |  |
| Sarcopenic obesity (HC) |  |  | 0.28 |
| Without sarcopenia and obesity | Ref. (0) | Ref. (0) |  |
| Only with sarcopenia | -0.02 (-0.06 to 0.02) | -0.11 (-0.18 to -0.04)^**^ |  |
| Only with obesity | -0.00004 (-0.05 to 0.05) | -0.04 (-0.12 to 0.05) |  |
| Sarcopenic obesity | 0.001 (-0.05 to 0.05) | -0.05 (-0.14 to 0.03) |  |
| Sarcopenic obesity (WHR) |  |  | 0.07 |
| Without sarcopenia and obesity | Ref. (0) | Ref. (0) |  |
| Only with sarcopenia | 0.01 (-0.05 to 0.08) | -0.13 (-0.21 to -0.05)^**^ |  |
| Only with obesity | -0.01 (-0.07 to 0.04) | -0.06 (-0.14 to 0.03) |  |
| Sarcopenic obesity | -0.04 (-0.09 to 0.02) | -0.09 (-0.17 to -0.01)^*^ |  |
| Sarcopenic obesity (WHtR) |  |  | 0.05 |
| Without sarcopenia and obesity | Ref. (0) | Ref. (0) |  |
| Only with sarcopenia | 0.002 (-0.04 to 0.04) | -0.11 (-0.19 to -0.04)^**^ |  |
| Only with obesity | -0.04 (-0.09 to 0.01) | -0.05 (-0.13 to 0.03) |  |
| Sarcopenic obesity | -0.06 (-0.11 to -0.02)^**^ | -0.09 (-0.17 to -0.01)^*^ |  |
| Sarcopenic obesity (LAP) |  |  | 0.005 |
| Without sarcopenia and obesity | Ref. (0) | Ref. (0) |  |
| Only with sarcopenia | -0.01 (-0.05 to 0.03) | -0.13 (-0.20 to -0.06)^***^ |  |
| Only with obesity | -0.06 (-0.11 to -0.001)^*^ | -0.07 (-0.15 to 0.02) |  |
| Sarcopenic obesity | -0.07 (-0.12 to -0.03)^**^ | -0.05 (-0.13 to 0.03) |  |
| Sarcopenic obesity (ABSI) |  |  | 0.23 |
| Without sarcopenia and obesity | Ref. (0) | Ref. (0) |  |
| Only with sarcopenia | 0.001 (-0.04 to 0.04) | -0.06 (-0.12 to 0.01) |  |
| Only with obesity | -0.01 (-0.07 to 0.04) | 0.05 (-0.04 to 0.13) |  |
| Sarcopenic obesity | -0.06 (-0.11 to -0.01)^*^ | -0.10 (-0.18 to -0.01)^*^ |  |
| Sarcopenic obesity (VAI) |  |  | 0.15 |
| Without sarcopenia and obesity | Ref. (0) | Ref. (0) |  |
| Only with sarcopenia | -0.01 (-0.05 to 0.03) | -0.11 (-0.18 to -0.04)^**^ |  |
| Only with obesity | -0.03 (-0.09 to 0.02) | -0.07 (-0.16 to 0.01) |  |
| Sarcopenic obesity | -0.06 (-0.11 to -0.01)^*^ | -0.09 (-0.17 to -0.01)^*^ |  |
| Sarcopenic obesity (CVAI) |  |  | 0.07 |
| Without sarcopenia and obesity | Ref. (0) | Ref. (0) |  |
| Only with sarcopenia | 0.004 (-0.04 to 0.04) | -0.10 (-0.17 to -0.03)^**^ |  |
| Only with obesity | -0.02 (-0.08 to 0.04) | -0.03 (-0.12 to 0.06) |  |
| Sarcopenic obesity | -0.06 (-0.11 to -0.01)^*^ | -0.08 (-0.16 to 0.002) |  |
| Sarcopenic obesity (BRI) |  |  | 0.02 |
| Without sarcopenia and obesity | Ref. (0) | Ref. (0) |  |
| Only with sarcopenia | 0.001 (-0.04 to 0.04) | -0.12 (-0.19 to -0.05)^***^ |  |
| Only with obesity | -0.03 (-0.09 to 0.02) | -0.09 (-0.18 to -0.003)^*^ |  |
| Sarcopenic obesity | -0.07 (-0.12 to -0.02)^**^ | -0.09 (-0.17 to -0.005)^*^ |  |
| Sarcopenic obesity (Conicity index) |  |  | 0.09 |
| Without sarcopenia and obesity | Ref. (0) | Ref. (0) |  |
| Only with sarcopenia | 0.00s (-0.04 to 0.04) | -0.04 (-0.10 to 0.03) |  |
| Only with obesity | 0.05 (-0.004 to 0.11) | 0.05 (-0.04 to 0.14) |  |
| Sarcopenic obesity | -0.0001 (-0.05 to 0.05) | -0.16 (-0.25 to -0.07)^***^ |  |
| Sarcopenic obesity (BAI) |  |  | 0.08 |
| Without sarcopenia and obesity | Ref. (0) | Ref. (0) |  |
| Only with sarcopenia | 0.001 (-0.04 to 0.04) | -0.11 (-0.18 to -0.04)^**^ |  |
| Only with obesity | 0.01 (-0.05 to 0.06) | -0.05 (-0.14 to 0.03) |  |
| Sarcopenic obesity | -0.03 (-0.08 to 0.01) | -0.07 (-0.16 to 0.01) |  |
| Sarcopenic obesity (CMI) |  |  | 0.06 |
| Without sarcopenia and obesity | Ref. (0) | Ref. (0) |  |
| Only with sarcopenia | -0.01 (-0.05 to 0.03) | -0.12 (-0.19 to -0.05)^***^ |  |
| Only with obesity | -0.04 (-0.10 to 0.01) | -0.08 (-0.17 to 0.004) |  |
| Sarcopenic obesity | -0.07 (-0.12 to -0.02)^**^ | -0.09 (-0.17 to -0.01)^*^ |  |
| Sarcopenic obesity (BSA) |  |  | 0.15 |
| Without sarcopenia and obesity | Ref. (0) | Ref. (0) |  |
| Only with sarcopenia | -0.03 (-0.07 to 0.02) | -0.13 (-0.20 to -0.06)^***^ |  |
| Only with obesity | -0.01 (-0.06 to 0.04) | -0.03 (-0.11 to 0.05) |  |
| Sarcopenic obesity | 0.001 (-0.04 to 0.05) | -0.03 (-0.11 to 0.06) |  |
| Sarcopenic obesity (WHHR) |  |  | 0.29 |
| Without sarcopenia and obesity | Ref. (0) | Ref. (0) |  |
| Only with sarcopenia | 0.002 (-0.04 to 0.04) | -0.08 (-0.15 to -0.01)^*^ |  |
| Only with obesity | -0.05 (-0.10 to 0.01) | -0.05 (-0.14 to 0.04) |  |
| Sarcopenic obesity | -0.08 (-0.13 to -0.03)^**^ | -0.13 (-0.21 to -0.05)^**^ |  |
| Sarcopenic obesity (PFM) |  |  | 0.10 |
| Without sarcopenia and obesity | Ref. (0) | Ref. (0) |  |
| Only with sarcopenia | -0.01 (-0.05 to 0.03) | -0.13 (-0.20 to -0.06)^***^ |  |
| Only with obesity | -0.01 (-0.06 to 0.04) | -0.06 (-0.15 to 0.02) |  |
| Sarcopenic obesity | -0.02 (-0.07 to 0.03) | -0.06 (-0.14 to 0.02) |  |
| Sarcopenic obesity (PLM) |  |  | 0.13 |
| Without sarcopenia and obesity | Ref. (0) | Ref. (0) |  |
| Only with sarcopenia | -0.03 (-0.07 to 0.02) | -0.14 (-0.21 to -0.07)^***^ |  |
| Only with obesity | -0.02 (-0.07 to 0.03) | -0.06 (-0.14 to 0.02) |  |
| Sarcopenic obesity | -0.01 (-0.05 to 0.04) | -0.05 (-0.13 to 0.04) |  |
| Sarcopenic obesity (PPF) |  |  | 0.20 |
| Without sarcopenia and obesity | Ref. (0) | Ref. (0) |  |
| Only with sarcopenia | -0.01 (-0.05 to 0.03) | -0.11 (-0.18 to -0.04)^**^ |  |
| Only with obesity | -0.02 (-0.07 to 0.04) | -0.08 (-0.17 to 0.01) |  |
| Sarcopenic obesity | -0.03 (-0.08 to 0.02) | -0.11 (-0.19 to -0.03)^*^ |  |
| Sarcopenic obesity (CUN-BAE) |  |  | 0.04 |
| Without sarcopenia and obesity | Ref. (0) | Ref. (0) |  |
| Only with sarcopenia | -0.01 (-0.05 to 0.03) | -0.13 (-0.20 to -0.06)^***^ |  |
| Only with obesity | -0.02 (-0.08 to 0.04) | -0.10 (-0.18 to -0.01)^*^ |  |
| Sarcopenic obesity | -0.03 (-0.08 to 0.01) | -0.08 (-0.16 to -0.0002)^*^ |  |

ABSI: a body shape index; BAI: body adiposity index; BMI: body mass index; BRI: body roundness index; BSA: body surface area; CI: confidence interval; CMI: cardiometabolic index; CUN-BAE: Clínica Universidad de Navarra-Body Adiposity Estimator; CVAI: Chinese visceral adiposity index; DWRT: Delayed Word Recall Test; HC, hip circumference; LAP: lipid accumulation product; PFM: predicted fat mass; PLM: predicted lean mass; PPF: predicated percent fat; Ref, reference; VAI: visceral adiposity index; WC: waist circumference; WHHR: waist-to-hip-to-height ratio; WHR: waist-to-hip ratio; WHtR: waist-to-height ratio

Note: Adjusted for sex, age, education, occupation, personal income, physical activity, drinking, smoking, self-rated health and baseline DWRT score

^*^P <0.05, ^**^P <0.01, ^***^P <0.001

Table S16. Associations of baseline sarcopenia and sarcopenic obesity with annual change of memory function by age.

|  | Adjusted mean differences β (95% CI) in annual change of DWRT score | | |
| --- | --- | --- | --- |
|  | ≤60 | >60 | P for interaction |
| Sarcopenia |  |  | 0.06 |
| No | Ref. (0) | Ref. (0) |  |
| Yes | -0.02 (-0.05 to 0.02) | -0.05 (-0.10 to 0.01) |  |
| Sarcopenic obesity (Weight) |  |  | 0.34 |
| Without sarcopenia and obesity | Ref. (0) | Ref. (0) |  |
| Only with sarcopenia | -0.04 (-0.08 to 0.004) | -0.07 (-0.13 to -0.005)^*^ |  |
| Only with obesity | -0.02 (-0.07 to 0.03) | -0.04 (-0.13 to 0.05) |  |
| Sarcopenic obesity | -0.01 (-0.06 to 0.04) | -0.04 (-0.11 to 0.03) |  |
| Sarcopenic obesity (BMI) |  |  | 0.16 |
| Without sarcopenia and obesity | Ref. (0) | Ref. (0) |  |
| Only with sarcopenia | -0.02 (-0.07 to 0.02) | -0.07 (-0.14 to -0.01)^*^ |  |
| Only with obesity | 0.002 (-0.05 to 0.05) | -0.06 (-0.15 to 0.03) |  |
| Sarcopenic obesity | -0.01 (-0.06 to 0.04) | -0.06 (-0.13 to 0.01) |  |
| Sarcopenic obesity (WC) |  |  | 0.17 |
| Without sarcopenia and obesity | Ref. (0) | Ref. (0) |  |
| Only with sarcopenia | -0.03 (-0.07 to 0.01) | -0.05 (-0.11 to 0.01) |  |
| Only with obesity | -0.06 (-0.12 to -0.005)^*^ | -0.07 (-0.16 to 0.03) |  |
| Sarcopenic obesity | -0.05 (-0.11 to 0.002) | -0.10 (-0.18 to -0.03)^**^ |  |
| Sarcopenic obesity (HC) |  |  | 0.19 |
| Without sarcopenia and obesity | Ref. (0) | Ref. (0) |  |
| Only with sarcopenia | -0.03 (-0.07 to 0.01) | -0.06 (-0.12 to 0.01) |  |
| Only with obesity | -0.03 (-0.08 to 0.02) | 0.02 (-0.08 to 0.11) |  |
| Sarcopenic obesity | -0.02 (-0.07 to 0.03) | -0.01 (-0.09 to 0.06) |  |
| Sarcopenic obesity (WHR) |  |  | 0.09 |
| Without sarcopenia and obesity | Ref. (0) | Ref. (0) |  |
| Only with sarcopenia | -0.04 (-0.10 to 0.02) | -0.06 (-0.15 to 0.04) |  |
| Only with obesity | -0.03 (-0.08 to 0.02) | -0.06 (-0.15 to 0.03) |  |
| Sarcopenic obesity | -0.04 (-0.09 to 0.01) | -0.10 (-0.18 to -0.02)^*^ |  |
| Sarcopenic obesity (WHtR) |  |  | 0.13 |
| Without sarcopenia and obesity | Ref. (0) | Ref. (0) |  |
| Only with sarcopenia | -0.03 (-0.07 to 0.02) | -0.04 (-0.12 to 0.03) |  |
| Only with obesity | -0.05 (-0.10 to 0.003) | -0.07 (-0.15 to 0.02) |  |
| Sarcopenic obesity | -0.05 (-0.10 to 0.001) | -0.11 (-0.18 to -0.03)^**^ |  |
| Sarcopenic obesity (LAP) |  |  | 0.24 |
| Without sarcopenia and obesity | Ref. (0) | Ref. (0) |  |
| Only with sarcopenia | -0.03 (-0.07 to 0.01) | -0.05 (-0.12 to 0.01) |  |
| Only with obesity | -0.06 (-0.11 to -0.01)^*^ | -0.07 (-0.16 to 0.02) |  |
| Sarcopenic obesity | -0.05 (-0.11 to -0.001)^*^ | -0.10 (-0.17 to -0.03)^**^ |  |
| Sarcopenic obesity (ABSI) |  |  | 0.31 |
| Without sarcopenia and obesity | Ref. (0) | Ref. (0) |  |
| Only with sarcopenia | -0.01 (-0.05 to 0.03) | -0.02 (-0.08 to 0.05) |  |
| Only with obesity | -0.02 (-0.07 to 0.04) | 0.03 (-0.06 to 0.12) |  |
| Sarcopenic obesity | -0.06 (-0.11 to 0.003) | -0.07 (-0.14 to -0.002)^*^ |  |
| Sarcopenic obesity (VAI) |  |  | 0.17 |
| Without sarcopenia and obesity | Ref. (0) | Ref. (0) |  |
| Only with sarcopenia | -0.02 (-0.06 to 0.03) | -0.07 (-0.13 to -0.002)^*^ |  |
| Only with obesity | -0.04 (-0.09 to 0.01) | -0.09 (-0.18 to -0.003)^*^ |  |
| Sarcopenic obesity | -0.06 (-0.12 to -0.01)^*^ | -0.10 (-0.17 to -0.02)^**^ |  |
| Sarcopenic obesity (CVAI) |  |  | 0.20 |
| Without sarcopenia and obesity | Ref. (0) | Ref. (0) |  |
| Only with sarcopenia | -0.02 (-0.06 to 0.02) | -0.04 (-0.11 to 0.02) |  |
| Only with obesity | -0.02 (-0.08 to 0.04) | -0.06 (-0.14 to 0.03) |  |
| Sarcopenic obesity | -0.05 (-0.10 to 0.01) | -0.10 (-0.18 to -0.03)^**^ |  |
| Sarcopenic obesity (BRI) |  |  | 0.17 |
| Without sarcopenia and obesity | Ref. (0) | Ref. (0) |  |
| Only with sarcopenia | -0.02 (-0.06 to 0.02) | -0.05 (-0.12 to 0.01) |  |
| Only with obesity | -0.05 (-0.11 to 0.01) | -0.09 (-0.18 to 0.003) |  |
| Sarcopenic obesity | -0.05 (-0.11 to 0.003) | -0.11 (-0.18 to -0.04)^**^ |  |
| Sarcopenic obesity (Conicity index) |  |  | 0.43 |
| Without sarcopenia and obesity | Ref. (0) | Ref. (0) |  |
| Only with sarcopenia | 0.001 (-0.04 to 0.04) | -0.03 (-0.10 to 0.03) |  |
| Only with obesity | 0.05 (-0.01 to 0.10) | 0.05 (-0.04 to 0.14) |  |
| Sarcopenic obesity | -0.04 (-0.10 to 0.01) | -0.03 (-0.10 to 0.05) |  |
| Sarcopenic obesity (BAI) |  |  | 0.21 |
| Without sarcopenia and obesity | Ref. (0) | Ref. (0) |  |
| Only with sarcopenia | -0.02 (-0.06 to 0.02) | -0.06 (-0.12 to 0.01) |  |
| Only with obesity | -0.001 (-0.05 to 0.05) | -0.05 (-0.14 to 0.04) |  |
| Sarcopenic obesity | -0.03 (-0.08 to 0.03) | -0.07 (-0.14 to -0.005)^*^ |  |
| Sarcopenic obesity (CMI) |  |  | 0.13 |
| Without sarcopenia and obesity | Ref. (0) | Ref. (0) |  |
| Only with sarcopenia | -0.01 (-0.05 to 0.03) | -0.07 (-0.13 to -0.01)^*^ |  |
| Only with obesity | -0.04 (-0.09 to 0.01) | -0.11 (-0.19 to -0.02)^*^ |  |
| Sarcopenic obesity | -0.07 (-0.13 to -0.02)^**^ | -0.10 (-0.17 to -0.03)^**^ |  |
| Sarcopenic obesity (BSA) |  |  | 0.19 |
| Without sarcopenia and obesity | Ref. (0) | Ref. (0) |  |
| Only with sarcopenia | -0.05 (-0.09 to -0.01)^*^ | -0.05 (-0.11 to 0.01) |  |
| Only with obesity | -0.03 (-0.08 to 0.02) | 0.03 (-0.06 to 0.12) |  |
| Sarcopenic obesity | 0.002 (-0.05 to 0.05) | -0.02 (-0.09 to 0.06) |  |
| Sarcopenic obesity (WHHR) |  |  | 0.36 |
| Without sarcopenia and obesity | Ref. (0) | Ref. (0) |  |
| Only with sarcopenia | -0.01 (-0.05 to 0.02) | -0.03 (-0.10 to 0.04) |  |
| Only with obesity | -0.06 (-0.12 to 0.004) | -0.06 (-0.15 to 0.03) |  |
| Sarcopenic obesity | -0.08 (-0.14 to -0.02)^**^ | -0.11 (-0.18 to -0.04)^**^ |  |
| Sarcopenic obesity (PFM) |  |  | 0.28 |
| Without sarcopenia and obesity | Ref. (0) | Ref. (0) |  |
| Only with sarcopenia | -0.03 (-0.07 to 0.01) | -0.06 (-0.12 to 0.01) |  |
| Only with obesity | -0.03 (-0.08 to 0.02) | -0.03 (-0.12 to 0.06) |  |
| Sarcopenic obesity | -0.02 (-0.07 to 0.03) | -0.06 (-0.13 to 0.01) |  |
| Sarcopenic obesity (PLM) |  |  | 0.38 |
| Without sarcopenia and obesity | Ref. (0) | Ref. (0) |  |
| Only with sarcopenia | -0.04 (-0.09 to -0.0003)^*^ | -0.06 (-0.13 to 0.0001) |  |
| Only with obesity | -0.03 (-0.08 to 0.02) | -0.02 (-0.11 to 0.07) |  |
| Sarcopenic obesity | -0.01 (-0.06 to 0.04) | -0.03 (-0.11 to 0.04) |  |
| Sarcopenic obesity (PPF) |  |  | 0.05 |
| Without sarcopenia and obesity | Ref. (0) | Ref. (0) |  |
| Only with sarcopenia | -0.03 (-0.07 to 0.01) | -0.06 (-0.13 to 0.004) |  |
| Only with obesity | -0.02 (-0.08 to 0.03) | -0.09 (-0.18 to 0.001) |  |
| Sarcopenic obesity | -0.02 (-0.07 to 0.04) | -0.10 (-0.17 to -0.03)^**^ |  |
| Sarcopenic obesity (CUN-BAE) |  |  | 0.14 |
| Without sarcopenia and obesity | Ref. (0) | Ref. (0) |  |
| Only with sarcopenia | -0.03 (-0.07 to 0.01) | -0.07 (-0.14 to 0.001) |  |
| Only with obesity | -0.04 (-0.10 to 0.02) | -0.07 (-0.16 to 0.01) |  |
| Sarcopenic obesity | -0.02 (-0.08 to 0.03) | -0.09 (-0.16 to -0.02)^*^ |  |

ABSI: a body shape index; BAI: body adiposity index; BMI: body mass index; BRI: body roundness index; BSA: body surface area; CI: confidence interval; CMI: cardiometabolic index; CUN-BAE: Clínica Universidad de Navarra-Body Adiposity Estimator; CVAI: Chinese visceral adiposity index; DWRT: Delayed Word Recall Test; HC, hip circumference; LAP: lipid accumulation product; PFM: predicted fat mass; PLM: predicted lean mass; PPF: predicated percent fat; Ref, reference; VAI: visceral adiposity index; WC: waist circumference; WHHR: waist-to-hip-to-height ratio; WHR: waist-to-hip ratio; WHtR: waist-to-height ratio

Note: Adjusted for sex, age, education, occupation, personal income, physical activity, drinking, smoking, self-rated health and baseline DWRT score

^*^P <0.05, ^**^P <0.01, ^***^P <0.001

Table S17. Associations of baseline sarcopenia and sarcopenic obesity with annual change of memory function by baseline memory status.

|  | Adjusted mean differences β (95% CI) in annual change of DWRT score | | |
| --- | --- | --- | --- |
|  | Baseline DWRT score ≥4 | Baseline DWRT score <4 | P for interaction |
| Sarcopenia |  |  | 0.07 |
| No | Ref. (0) | Ref. (0) |  |
| Yes | -0.03 (-0.06 to 0.0003) | -0.06 (-0.19 to 0.07) |  |
| Sarcopenic obesity (Weight) |  |  | 0.35 |
| Without sarcopenia and obesity | Ref. (0) | Ref. (0) |  |
| Only with sarcopenia | -0.05 (-0.09 to -0.01)^**^ | -0.06 (-0.22 to 0.09) |  |
| Only with obesity | -0.02 (-0.07 to 0.02) | -0.07 (-0.31 to 0.16) |  |
| Sarcopenic obesity | -0.01 (-0.06 to 0.03) | -0.10 (-0.27 to 0.07) |  |
| Sarcopenic obesity (BMI) |  |  | 0.21 |
| Without sarcopenia and obesity | Ref. (0) | Ref. (0) |  |
| Only with sarcopenia | -0.04 (-0.07 to -0.002)^*^ | -0.02 (-0.19 to 0.14) |  |
| Only with obesity | -0.01 (-0.06 to 0.03) | 0.13 (-0.10 to 0.36) |  |
| Sarcopenic obesity | -0.03 (-0.07 to 0.01) | -0.003 (-0.17 to 0.17) |  |
| Sarcopenic obesity (WC) |  |  | 0.14 |
| Without sarcopenia and obesity | Ref. (0) | Ref. (0) |  |
| Only with sarcopenia | -0.03 (-0.07 to -0.0001)^*^ | -0.01 (-0.17 to 0.14) |  |
| Only with obesity | -0.06 (-0.11 to -0.01)^*^ | 0.07 (-0.18 to 0.32) |  |
| Sarcopenic obesity | -0.07 (-0.12 to -0.03)^**^ | -0.09 (-0.27 to 0.08) |  |
| Sarcopenic obesity (HC) |  |  | 0.07 |
| Without sarcopenia and obesity | Ref. (0) | Ref. (0) |  |
| Only with sarcopenia | -0.04 (-0.08 to -0.01)^*^ | -0.02 (-0.17 to 0.13) |  |
| Only with obesity | -0.02 (-0.07 to 0.03) | 0.18 (-0.06 to 0.42) |  |
| Sarcopenic obesity | -0.02 (-0.06 to 0.02) | 0.01 (-0.17 to 0.19) |  |
| Sarcopenic obesity (WHR) |  |  | 0.21 |
| Without sarcopenia and obesity | Ref. (0) | Ref. (0) |  |
| Only with sarcopenia | -0.04 (-0.09 to 0.01) | -0.08 (-0.32 to 0.16) |  |
| Only with obesity | -0.03 (-0.08 to 0.01) | -0.10 (-0.33 to 0.12) |  |
| Sarcopenic obesity | -0.06 (-0.10 to -0.01)^*^ | -0.14 (-0.36 to 0.07) |  |
| Sarcopenic obesity (WHtR) |  |  | 0.12 |
| Without sarcopenia and obesity | Ref. (0) | Ref. (0) |  |
| Only with sarcopenia | -0.03 (-0.07 to 0.01) | 0.02 (-0.15 to 0.20) |  |
| Only with obesity | -0.05 (-0.09 to -0.01)^*^ | 0.02 (-0.19 to 0.23) |  |
| Sarcopenic obesity | -0.07 (-0.11 to -0.03)^***^ | -0.10 (-0.28 to 0.07) |  |
| Sarcopenic obesity (LAP) |  |  | 0.23 |
| Without sarcopenia and obesity | Ref. (0) | Ref. (0) |  |
| Only with sarcopenia | -0.04 (-0.07 to -0.003)^*^ | -0.02 (-0.17 to 0.14) |  |
| Only with obesity | -0.06 (-0.11 to -0.02)^**^ | -0.02 (-0.24 to 0.21) |  |
| Sarcopenic obesity | -0.07 (-0.11 to -0.03)^**^ | -0.14 (-0.30 to 0.03) |  |
| Sarcopenic obesity (ABSI) |  |  | 0.21 |
| Without sarcopenia and obesity | Ref. (0) | Ref. (0) |  |
| Only with sarcopenia | -0.01 (-0.05 to 0.02) | -0.02 (-0.18 to 0.14) |  |
| Only with obesity | -0.005 (-0.05 to 0.04) | 0.07 (-0.16 to 0.29) |  |
| Sarcopenic obesity | -0.07 (-0.12 to -0.03)^**^ | -0.06 (-0.24 to 0.12) |  |
| Sarcopenic obesity (VAI) |  |  | 0.27 |
| Without sarcopenia and obesity | Ref. (0) | Ref. (0) |  |
| Only with sarcopenia | -0.03 (-0.07 to 0.001) | -0.03 (-0.19 to 0.13) |  |
| Only with obesity | -0.05 (-0.10 to -0.01)^*^ | -0.02 (-0.24 to 0.20) |  |
| Sarcopenic obesity | -0.07 (-0.11 to -0.03)^**^ | -0.13 (-0.30 to 0.04) |  |
| Sarcopenic obesity (CVAI) |  |  | 0.07 |
| Without sarcopenia and obesity | Ref. (0) | Ref. (0) |  |
| Only with sarcopenia | -0.02 (-0.06 to 0.01) | -0.01 (-0.17 to 0.14) |  |
| Only with obesity | -0.02 (-0.07 to 0.02) | -0.03 (-0.27 to 0.20) |  |
| Sarcopenic obesity | -0.07 (-0.11 to -0.02)^**^ | -0.16 (-0.32 to 0.01) |  |
| Sarcopenic obesity (BRI) |  |  | 0.26 |
| Without sarcopenia and obesity | Ref. (0) | Ref. (0) |  |
| Only with sarcopenia | -0.03 (-0.06 to 0.01) | -0.04 (-0.20 to 0.12) |  |
| Only with obesity | -0.05 (-0.10 to -0.01)^*^ | -0.04 (-0.26 to 0.19) |  |
| Sarcopenic obesity | -0.07 (-0.12 to -0.03)^***^ | -0.11 (-0.27 to 0.06) |  |
| Sarcopenic obesity (Conicity index) |  |  | 0.28 |
| Without sarcopenia and obesity | Ref. (0) | Ref. (0) |  |
| Only with sarcopenia | -0.01 (-0.04 to 0.03) | -0.06 (-0.22 to 0.10) |  |
| Only with obesity | 0.05 (0.003 to 0.10)^*^ | 0.001 (-0.22 to 0.22) |  |
| Sarcopenic obesity | -0.04 (-0.08 to 0.01) | -0.06 (-0.24 to 0.13) |  |
| Sarcopenic obesity (BAI) |  |  | 0.007 |
| Without sarcopenia and obesity | Ref. (0) | Ref. (0) |  |
| Only with sarcopenia | -0.03 (-0.06 to 0.01) | 0.03 (-0.13 to 0.19) |  |
| Only with obesity | -0.03 (-0.07 to 0.02) | 0.27 (0.05 to 0.49)^*^ |  |
| Sarcopenic obesity | -0.06 (-0.10 to -0.01)^*^ | 0.05 (-0.12 to 0.22) |  |
| Sarcopenic obesity (CMI) |  |  | 0.25 |
| Without sarcopenia and obesity | Ref. (0) | Ref. (0) |  |
| Only with sarcopenia | -0.03 (-0.07 to 0.002) | -0.04 (-0.19 to 0.12) |  |
| Only with obesity | -0.06 (-0.10 to -0.01)^*^ | -0.07 (-0.29 to 0.16) |  |
| Sarcopenic obesity | -0.07 (-0.12 to -0.03)^***^ | -0.16 (-0.33 to 0.01) |  |
| Sarcopenic obesity (BSA) |  |  | 0.28 |
| Without sarcopenia and obesity | Ref. (0) | Ref. (0) |  |
| Only with sarcopenia | -0.05 (-0.09 to -0.01)^**^ | -0.09 (-0.25 to 0.06) |  |
| Only with obesity | -0.01 (-0.05 to 0.03) | -0.10 (-0.33 to 0.14) |  |
| Sarcopenic obesity | -0.003 (-0.05 to 0.04) | -0.06 (-0.24 to 0.11) |  |
| Sarcopenic obesity (WHHR) |  |  | 0.003 |
| Without sarcopenia and obesity | Ref. (0) | Ref. (0) |  |
| Only with sarcopenia | -0.02 (-0.06 to 0.01) | 0.00 (-0.16 to 0.15) |  |
| Only with obesity | -0.04 (-0.09 to 0.01) | -0.11 (-0.34 to 0.11) |  |
| Sarcopenic obesity | -0.08 (-0.12 to -0.04)^***^ | -0.21 (-0.38 to -0.05)^*^ |  |
| Sarcopenic obesity (PFM) |  |  | 0.23 |
| Without sarcopenia and obesity | Ref. (0) | Ref. (0) |  |
| Only with sarcopenia | -0.04 (-0.08 to -0.003)^*^ | -0.08 (-0.24 to 0.08) |  |
| Only with obesity | -0.02 (-0.07 to 0.02) | -0.11 (-0.33 to 0.12) |  |
| Sarcopenic obesity | -0.03 (-0.07 to 0.01) | -0.10 (-0.27 to 0.07) |  |
| Sarcopenic obesity (PLM) |  |  | 0.09 |
| Without sarcopenia and obesity | Ref. (0) | Ref. (0) |  |
| Only with sarcopenia | -0.05 (-0.09 to -0.01)^**^ | -0.12 (-0.27 to 0.04) |  |
| Only with obesity | -0.02 (-0.06 to 0.02) | -0.23 (-0.46 to -0.002)^*^ |  |
| Sarcopenic obesity | -0.01 (-0.05 to 0.03) | -0.13 (-0.31 to 0.05) |  |
| Sarcopenic obesity (PPF) |  |  | 0.17 |
| Without sarcopenia and obesity | Ref. (0) | Ref. (0) |  |
| Only with sarcopenia | -0.04 (-0.07 to -0.0005)^*^ | -0.003 (-0.16 to 0.16) |  |
| Only with obesity | -0.04 (-0.09 to 0.01) | 0.12 (-0.12 to 0.35) |  |
| Sarcopenic obesity | -0.05 (-0.09 to -0.01)^*^ | -0.05 (-0.21 to 0.12) |  |
| Sarcopenic obesity (CUN-BAE) |  |  | 0.18 |
| Without sarcopenia and obesity | Ref. (0) | Ref. (0) |  |
| Only with sarcopenia | -0.04 (-0.08 to -0.004)^*^ | 0.01 (-0.15 to 0.17) |  |
| Only with obesity | -0.05 (-0.10 to -0.001)^*^ | 0.20 (-0.05 to 0.44) |  |
| Sarcopenic obesity | -0.05 (-0.09 to -0.01)^*^ | -0.02 (-0.18 to 0.15) |  |

ABSI: a body shape index; BAI: body adiposity index; BMI: body mass index; BRI: body roundness index; BSA: body surface area; CI: confidence interval; CMI: cardiometabolic index; CUN-BAE: Clínica Universidad de Navarra-Body Adiposity Estimator; CVAI: Chinese visceral adiposity index; DWRT: Delayed Word Recall Test; HC, hip circumference; LAP: lipid accumulation product; PFM: predicted fat mass; PLM: predicted lean mass; PPF: predicated percent fat; Ref, reference; VAI: visceral adiposity index; WC: waist circumference; WHHR: waist-to-hip-to-height ratio; WHR: waist-to-hip ratio; WHtR: waist-to-height ratio

Note: Adjusted for sex, age, education, occupation, personal income, physical activity, drinking, smoking, self-rated health and baseline DWRT score

^*^P <0.05, ^**^P <0.01, ^***^P <0.001

Table S18. Associations of baseline sarcopenia and sarcopenic obesity with annual change rate of memory function by sex.

|  | Adjusted mean differences β (95% CI) in annual change rate of DWRT score | | |
| --- | --- | --- | --- |
|  | Women | Men | P for interaction |
| Sarcopenia |  |  | 0.32 |
| No | Ref. (0) | Ref. (0) |  |
| Yes | -0.36 (-1.26 to 0.54) | -2.00 (-3.53 to -0.48)^*^ |  |
| Sarcopenic obesity (Weight) |  |  | 0.20 |
| Without sarcopenia and obesity | Ref. (0) | Ref. (0) |  |
| Only with sarcopenia | -0.40 (-1.50 to 0.69) | -3.40 (-5.29 to -1.52)^***^ |  |
| Only with obesity | -0.48 (-1.84 to 0.88) | -1.60 (-3.75 to 0.55) |  |
| Sarcopenic obesity | -0.71 (-1.96 to 0.55) | -1.24 (-3.40 to 0.91) |  |
| Sarcopenic obesity (BMI) |  |  | 0.11 |
| Without sarcopenia and obesity | Ref. (0) | Ref. (0) |  |
| Only with sarcopenia | 0.12 (-0.97 to 1.21) | -2.96 (-4.81 to -1.12)^**^ |  |
| Only with obesity | 0.91 (-0.47 to 2.28) | -1.48 (-3.73 to 0.77) |  |
| Sarcopenic obesity | -0.40 (-1.63 to 0.84) | -1.63 (-3.75 to 0.49) |  |
| Sarcopenic obesity (WC) |  |  | 0.07 |
| Without sarcopenia and obesity | Ref. (0) | Ref. (0) |  |
| Only with sarcopenia | 0.20 (-0.86 to 1.27) | -2.35 (-3.99 to -0.70)^**^ |  |
| Only with obesity | -0.90 (-2.33 to 0.53) | -0.93 (-3.95 to 2.08) |  |
| Sarcopenic obesity | -2.26 (-3.54 to -0.99)^***^ | -1.08 (-3.78 to 1.63) |  |
| Sarcopenic obesity (HC) |  |  | 0.22 |
| Without sarcopenia and obesity | Ref. (0) | Ref. (0) |  |
| Only with sarcopenia | -0.53 (-1.60 to 0.53) | -3.14 (-4.93 to -1.35)^***^ |  |
| Only with obesity | -0.77 (-2.17 to 0.62) | -1.12 (-3.38 to 1.14) |  |
| Sarcopenic obesity | -0.70 (-1.99 to 0.59) | -0.56 (-2.79 to 1.67) |  |
| Sarcopenic obesity (WHR) |  |  | 0.04 |
| Without sarcopenia and obesity | Ref. (0) | Ref. (0) |  |
| Only with sarcopenia | 1.03 (-0.66 to 2.72) | -3.12 (-5.21 to -1.02)^**^ |  |
| Only with obesity | 0.40 (-0.99 to 1.79) | -1.94 (-4.05 to 0.16) |  |
| Sarcopenic obesity | -0.47 (-1.88 to 0.94) | -2.65 (-4.66 to -0.63)^*^ |  |
| Sarcopenic obesity (WHtR) |  |  | 0.006 |
| Without sarcopenia and obesity | Ref. (0) | Ref. (0) |  |
| Only with sarcopenia | 0.44 (-0.70 to 1.58) | -3.39 (-5.39 to -1.39)^***^ |  |
| Only with obesity | -0.44 (-1.77 to 0.90) | -1.96 (-4.09 to 0.17) |  |
| Sarcopenic obesity | -1.76 (-2.99 to -0.53)^**^ | -2.17 (-4.21 to -0.13)^*^ |  |
| Sarcopenic obesity (LAP) |  |  | 0.02 |
| Without sarcopenia and obesity | Ref. (0) | Ref. (0) |  |
| Only with sarcopenia | 0.18 (-0.88 to 1.24) | -3.01 (-4.85 to -1.16)^**^ |  |
| Only with obesity | -0.47 (-1.90 to 0.95) | -1.45 (-3.68 to 0.77) |  |
| Sarcopenic obesity | -1.94 (-3.23 to -0.65)^**^ | -1.54 (-3.69 to 0.60) |  |
| Sarcopenic obesity (ABSI) |  |  | 0.46 |
| Without sarcopenia and obesity | Ref. (0) | Ref. (0) |  |
| Only with sarcopenia | 0.11 (-0.92 to 1.14) | -1.75 (-3.53 to 0.03) |  |
| Only with obesity | 0.11 (-1.39 to 1.61) | 0.25 (-2.05 to 2.55) |  |
| Sarcopenic obesity | -1.49 (-2.82 to -0.15)^*^ | -2.35 (-4.61 to -0.09)^*^ |  |
| Sarcopenic obesity (VAI) |  |  | 0.15 |
| Without sarcopenia and obesity | Ref. (0) | Ref. (0) |  |
| Only with sarcopenia | 0.08 (-0.99 to 1.16) | -2.76 (-4.61 to -0.91)^**^ |  |
| Only with obesity | -0.08 (-1.46 to 1.29) | -1.07 (-3.32 to 1.18) |  |
| Sarcopenic obesity | -1.44 (-2.75 to -0.13)^*^ | -1.64 (-3.76 to 0.47) |  |
| Sarcopenic obesity (CVAI) |  |  | 0.049 |
| Without sarcopenia and obesity | Ref. (0) | Ref. (0) |  |
| Only with sarcopenia | 0.28 (-0.76 to 1.32) | -2.56 (-4.40 to -0.72)^**^ |  |
| Only with obesity | -0.18 (-1.73 to 1.37) | -1.18 (-3.45 to 1.09) |  |
| Sarcopenic obesity | -1.98 (-3.30 to -0.66)^**^ | -2.02 (-4.14 to 0.11) |  |
| Sarcopenic obesity (BRI) |  |  | <0.001 |
| Without sarcopenia and obesity | Ref. (0) | Ref. (0) |  |
| Only with sarcopenia | 0.32 (-0.73 to 1.36) | -3.55 (-5.37 to -1.72)^***^ |  |
| Only with obesity | -0.16 (-1.68 to 1.36) | -2.90 (-5.20 to -0.60)^*^ |  |
| Sarcopenic obesity | -1.95 (-3.24 to -0.67)^**^ | -1.68 (-3.81 to 0.45) |  |
| Sarcopenic obesity (Conicity index) |  |  | 0.70 |
| Without sarcopenia and obesity | Ref. (0) | Ref. (0) |  |
| Only with sarcopenia | -0.25 (-1.29 to 0.79) | -1.44 (-3.18 to 0.30) |  |
| Only with obesity | 0.78 (-0.72 to 2.29) | 0.79 (-1.61 to 3.18) |  |
| Sarcopenic obesity | 0.10 (-1.24 to 1.43) | -2.88 (-5.27 to -0.50)^*^ |  |
| Sarcopenic obesity (BAI) |  |  | 0.06 |
| Without sarcopenia and obesity | Ref. (0) | Ref. (0) |  |
| Only with sarcopenia | -0.001 (-1.06 to 1.06) | -2.93 (-4.76 to -1.10)^**^ |  |
| Only with obesity | 0.32 (-1.13 to 1.77) | -0.92 (-3.22 to 1.39) |  |
| Sarcopenic obesity | -0.81 (-2.08 to 0.45) | -1.14 (-3.27 to 0.99) |  |
| Sarcopenic obesity (CMI) |  |  | 0.08 |
| Without sarcopenia and obesity | Ref. (0) | Ref. (0) |  |
| Only with sarcopenia | 0.13 (-0.94 to 1.20) | -2.84 (-4.69 to -0.99)^**^ |  |
| Only with obesity | -0.22 (-1.62 to 1.17) | -1.35 (-3.61 to 0.91) |  |
| Sarcopenic obesity | -1.63 (-2.93 to -0.34)^*^ | -1.73 (-3.83 to 0.37) |  |
| Sarcopenic obesity (BSA) |  |  | 0.73 |
| Without sarcopenia and obesity | Ref. (0) | Ref. (0) |  |
| Only with sarcopenia | -0.67 (-1.77 to 0.43) | -2.71 (-4.59 to -0.83)^**^ |  |
| Only with obesity | -0.53 (-1.86 to 0.79) | -0.69 (-2.83 to 1.45) |  |
| Sarcopenic obesity | -0.32 (-1.60 to 0.97) | -1.44 (-3.66 to 0.77) |  |
| Sarcopenic obesity (WHHR) |  |  | 0.36 |
| Without sarcopenia and obesity | Ref. (0) | Ref. (0) |  |
| Only with sarcopenia | 0.32 (-0.72 to 1.35) | -1.92 (-3.72 to -0.12)^*^ |  |
| Only with obesity | -0.41 (-1.99 to 1.17) | -2.20 (-4.59 to 0.19) |  |
| Sarcopenic obesity | -2.22 (-3.52 to -0.92)^***^ | -3.92 (-6.08 to -1.77)^***^ |  |
| Sarcopenic obesity (PFM) |  |  | 0.09 |
| Without sarcopenia and obesity | Ref. (0) | Ref. (0) |  |
| Only with sarcopenia | -0.39 (-1.47 to 0.69) | -3.38 (-5.24 to -1.53)^***^ |  |
| Only with obesity | -0.94 (-2.32 to 0.44) | -1.64 (-3.85 to 0.57) |  |
| Sarcopenic obesity | -1.12 (-2.37 to 0.14) | -1.18 (-3.29 to 0.94) |  |
| Sarcopenic obesity (PLM) |  |  | 0.58 |
| Without sarcopenia and obesity | Ref. (0) | Ref. (0) |  |
| Only with sarcopenia | -0.65 (-1.76 to 0.45) | -3.11 (-4.99 to -1.22)^**^ |  |
| Only with obesity | -0.80 (-2.12 to 0.53) | -1.15 (-3.27 to 0.98) |  |
| Sarcopenic obesity | -0.61 (-1.89 to 0.67) | -1.28 (-3.49 to 0.93) |  |
| Sarcopenic obesity (PPF) |  |  | 0.23 |
| Without sarcopenia and obesity | Ref. (0) | Ref. (0) |  |
| Only with sarcopenia | -0.31 (-1.37 to 0.74) | -2.85 (-4.67 to -1.02)^**^ |  |
| Only with obesity | -1.07 (-2.57 to 0.43) | -1.92 (-4.24 to 0.41) |  |
| Sarcopenic obesity | -1.28 (-2.55 to -0.01)^*^ | -2.06 (-4.18 to 0.07) |  |
| Sarcopenic obesity (CUN-BAE) |  |  | 0.04 |
| Without sarcopenia and obesity | Ref. (0) | Ref. (0) |  |
| Only with sarcopenia | -0.26 (-1.32 to 0.81) | -3.12 (-4.97 to -1.28)^***^ |  |
| Only with obesity | -1.19 (-2.68 to 0.30) | -1.30 (-3.57 to 0.97) |  |
| Sarcopenic obesity | -1.45 (-2.71 to -0.20)^*^ | -1.22 (-3.35 to 0.90) |  |

ABSI: a body shape index; BAI: body adiposity index; BMI: body mass index; BRI: body roundness index; BSA: body surface area; CI: confidence interval; CMI: cardiometabolic index; CUN-BAE: Clínica Universidad de Navarra-Body Adiposity Estimator; CVAI: Chinese visceral adiposity index; DWRT: Delayed Word Recall Test; HC, hip circumference; LAP: lipid accumulation product; PFM: predicted fat mass; PLM: predicted lean mass; PPF: predicated percent fat; Ref, reference; VAI: visceral adiposity index; WC: waist circumference; WHHR: waist-to-hip-to-height ratio; WHR: waist-to-hip ratio; WHtR: waist-to-height ratio

Note: Adjusted for sex, age, education, occupation, personal income, physical activity, drinking, smoking, self-rated health and baseline DWRT score

^*^P <0.05, ^**^P <0.01, ^***^P <0.001

Table S19. Associations of baseline sarcopenia and sarcopenic obesity with annual change rate of memory function by age.

|  | Adjusted mean differences β (95% CI) in annual change rate of DWRT score | | |
| --- | --- | --- | --- |
|  | ≤60 | >60 | P for interaction |
| Sarcopenia |  |  | 0.36 |
| No | Ref. (0) | Ref. (0) |  |
| Yes | -0.63 (-1.54 to 0.28) | -1.01 (-2.44 to 0.43) |  |
| Sarcopenic obesity (Weight) |  |  | 0.80 |
| Without sarcopenia and obesity | Ref. (0) | Ref. (0) |  |
| Only with sarcopenia | -0.96 (-2.10 to 0.18) | -1.49 (-3.21 to 0.22) |  |
| Only with obesity | -0.74 (-2.03 to 0.54) | -1.38 (-3.76 to 1.00) |  |
| Sarcopenic obesity | -0.75 (-2.07 to 0.56) | -1.35 (-3.29 to 0.59) |  |
| Sarcopenic obesity (BMI) |  |  | 0.39 |
| Without sarcopenia and obesity | Ref. (0) | Ref. (0) |  |
| Only with sarcopenia | -0.40 (-1.52 to 0.71) | -1.31 (-3.06 to 0.44) |  |
| Only with obesity | 0.65 (-0.68 to 1.97) | -1.30 (-3.68 to 1.09) |  |
| Sarcopenic obesity | -0.50 (-1.83 to 0.83) | -1.58 (-3.44 to 0.28) |  |
| Sarcopenic obesity (WC) |  |  | 0.68 |
| Without sarcopenia and obesity | Ref. (0) | Ref. (0) |  |
| Only with sarcopenia | -0.45 (-1.51 to 0.61) | -0.70 (-2.36 to 0.96) |  |
| Only with obesity | -1.18 (-2.63 to 0.27) | -1.33 (-3.91 to 1.25) |  |
| Sarcopenic obesity | -2.08 (-3.52 to -0.64)^**^ | -2.81 (-4.76 to -0.85)^**^ |  |
| Sarcopenic obesity (HC) |  |  | 0.12 |
| Without sarcopenia and obesity | Ref. (0) | Ref. (0) |  |
| Only with sarcopenia | -0.88 (-1.98 to 0.21) | -1.36 (-3.03 to 0.31) |  |
| Only with obesity | -1.45 (-2.79 to -0.12)^*^ | 0.43 (-2.03 to 2.89) |  |
| Sarcopenic obesity | -1.43 (-2.81 to -0.05)^*^ | 0.15 (-1.80 to 2.10) |  |
| Sarcopenic obesity (WHR) |  |  | 0.049 |
| Without sarcopenia and obesity | Ref. (0) | Ref. (0) |  |
| Only with sarcopenia | -1.03 (-2.57 to 0.52) | -0.15 (-2.64 to 2.35) |  |
| Only with obesity | -0.39 (-1.67 to 0.89) | -1.35 (-3.73 to 1.03) |  |
| Sarcopenic obesity | -0.80 (-2.14 to 0.53) | -2.53 (-4.74 to -0.32)^*^ |  |
| Sarcopenic obesity (WHtR) |  |  | 0.43 |
| Without sarcopenia and obesity | Ref. (0) | Ref. (0) |  |
| Only with sarcopenia | -0.68 (-1.83 to 0.47) | -0.43 (-2.37 to 1.52) |  |
| Only with obesity | -0.98 (-2.28 to 0.32) | -1.22 (-3.47 to 1.03) |  |
| Sarcopenic obesity | -1.40 (-2.71 to -0.08)^*^ | -2.46 (-4.35 to -0.58)^*^ |  |
| Sarcopenic obesity (LAP) |  |  | 0.84 |
| Without sarcopenia and obesity | Ref. (0) | Ref. (0) |  |
| Only with sarcopenia | -0.42 (-1.51 to 0.66) | -0.86 (-2.58 to 0.86) |  |
| Only with obesity | -0.89 (-2.25 to 0.48) | -0.90 (-3.29 to 1.50) |  |
| Sarcopenic obesity | -1.90 (-3.30 to -0.49)^**^ | -2.02 (-3.91 to -0.12)^*^ |  |
| Sarcopenic obesity (ABSI) |  |  | 0.77 |
| Without sarcopenia and obesity | Ref. (0) | Ref. (0) |  |
| Only with sarcopenia | -0.45 (-1.48 to 0.59) | -0.20 (-1.95 to 1.55) |  |
| Only with obesity | -0.23 (-1.71 to 1.25) | 0.73 (-1.64 to 3.09) |  |
| Sarcopenic obesity | -1.46 (-3.01 to 0.08) | -1.67 (-3.58 to 0.24) |  |
| Sarcopenic obesity (VAI) |  |  | 0.81 |
| Without sarcopenia and obesity | Ref. (0) | Ref. (0) |  |
| Only with sarcopenia | -0.39 (-1.48 to 0.70) | -0.96 (-2.69 to 0.78) |  |
| Only with obesity | -0.36 (-1.69 to 0.96) | -0.83 (-3.21 to 1.56) |  |
| Sarcopenic obesity | -1.54 (-2.96 to -0.12)^*^ | -1.81 (-3.71 to 0.09) |  |
| Sarcopenic obesity (CVAI) |  |  | 0.63 |
| Without sarcopenia and obesity | Ref. (0) | Ref. (0) |  |
| Only with sarcopenia | -0.34 (-1.37 to 0.70) | -0.79 (-2.61 to 1.03) |  |
| Only with obesity | -0.32 (-1.88 to 1.25) | -1.53 (-3.83 to 0.77) |  |
| Sarcopenic obesity | -1.74 (-3.24 to -0.25)^*^ | -2.62 (-4.52 to -0.72)^**^ |  |
| Sarcopenic obesity (BRI) |  |  | 0.62 |
| Without sarcopenia and obesity | Ref. (0) | Ref. (0) |  |
| Only with sarcopenia | -0.41 (-1.46 to 0.65) | -1.19 (-2.95 to 0.56) |  |
| Only with obesity | -0.79 (-2.27 to 0.70) | -2.06 (-4.46 to 0.34) |  |
| Sarcopenic obesity | -1.84 (-3.28 to -0.40)^*^ | -2.37 (-4.23 to -0.51)^*^ |  |
| Sarcopenic obesity (Conicity index) |  |  | 0.51 |
| Without sarcopenia and obesity | Ref. (0) | Ref. (0) |  |
| Only with sarcopenia | -0.43 (-1.46 to 0.60) | -0.64 (-2.39 to 1.11) |  |
| Only with obesity | 0.25 (-1.26 to 1.77) | 1.75 (-0.60 to 4.09) |  |
| Sarcopenic obesity | -1.08 (-2.63 to 0.47) | 0.15 (-1.82 to 2.12) |  |
| Sarcopenic obesity (BAI) |  |  | 0.81 |
| Without sarcopenia and obesity | Ref. (0) | Ref. (0) |  |
| Only with sarcopenia | -0.55 (-1.63 to 0.52) | -1.06 (-2.80 to 0.68) |  |
| Only with obesity | -0.12 (-1.52 to 1.29) | 0.01 (-2.41 to 2.43) |  |
| Sarcopenic obesity | -0.91 (-2.31 to 0.49) | -0.92 (-2.78 to 0.94) |  |
| Sarcopenic obesity (CMI) |  |  | 0.72 |
| Without sarcopenia and obesity | Ref. (0) | Ref. (0) |  |
| Only with sarcopenia | -0.30 (-1.39 to 0.79) | -1.03 (-2.75 to 0.70) |  |
| Only with obesity | -0.49 (-1.84 to 0.85) | -1.02 (-3.41 to 1.38) |  |
| Sarcopenic obesity | -1.81 (-3.21 to -0.41)^*^ | -1.84 (-3.73 to 0.05) |  |
| Sarcopenic obesity (BSA) |  |  | 0.71 |
| Without sarcopenia and obesity | Ref. (0) | Ref. (0) |  |
| Only with sarcopenia | -1.23 (-2.38 to -0.08)^*^ | -1.05 (-2.75 to 0.64) |  |
| Only with obesity | -0.84 (-2.10 to 0.41) | -0.05 (-2.45 to 2.35) |  |
| Sarcopenic obesity | -0.48 (-1.81 to 0.85) | -0.95 (-2.94 to 1.04) |  |
| Sarcopenic obesity (WHHR) |  |  | 0.85 |
| Without sarcopenia and obesity | Ref. (0) | Ref. (0) |  |
| Only with sarcopenia | -0.33 (-1.36 to 0.70) | -0.16 (-1.93 to 1.61) |  |
| Only with obesity | -1.02 (-2.60 to 0.56) | -1.22 (-3.62 to 1.19) |  |
| Sarcopenic obesity | -2.38 (-3.90 to -0.87)^**^ | -2.88 (-4.71 to -1.05)^**^ |  |
| Sarcopenic obesity (PFM) |  |  | 0.81 |
| Without sarcopenia and obesity | Ref. (0) | Ref. (0) |  |
| Only with sarcopenia | -1.02 (-2.12 to 0.09) | -1.34 (-3.09 to 0.40) |  |
| Only with obesity | -1.37 (-2.69 to -0.04)^*^ | -1.09 (-3.46 to 1.27) |  |
| Sarcopenic obesity | -1.09 (-2.44 to 0.25) | -1.41 (-3.31 to 0.48) |  |
| Sarcopenic obesity (PLM) |  |  | 0.88 |
| Without sarcopenia and obesity | Ref. (0) | Ref. (0) |  |
| Only with sarcopenia | -1.18 (-2.35 to -0.02)^*^ | -1.27 (-2.95 to 0.42) |  |
| Only with obesity | -0.98 (-2.22 to 0.27) | -0.72 (-3.14 to 1.70) |  |
| Sarcopenic obesity | -0.74 (-2.06 to 0.57) | -1.10 (-3.10 to 0.90) |  |
| Sarcopenic obesity (PPF) |  |  | 0.23 |
| Without sarcopenia and obesity | Ref. (0) | Ref. (0) |  |
| Only with sarcopenia | -0.96 (-2.01 to 0.10) | -1.27 (-3.05 to 0.51) |  |
| Only with obesity | -1.20 (-2.69 to 0.29) | -2.35 (-4.71 to 0.01) |  |
| Sarcopenic obesity | -0.74 (-2.17 to 0.68) | -2.54 (-4.40 to -0.68)^**^ |  |
| Sarcopenic obesity (CUN-BAE) |  |  | 0.46 |
| Without sarcopenia and obesity | Ref. (0) | Ref. (0) |  |
| Only with sarcopenia | -0.98 (-2.04 to 0.08) | -1.10 (-2.92 to 0.72) |  |
| Only with obesity | -1.47 (-2.96 to 0.02) | -1.48 (-3.78 to 0.83) |  |
| Sarcopenic obesity | -0.88 (-2.28 to 0.53) | -2.13 (-4.01 to -0.25)^*^ |  |

ABSI: a body shape index; BAI: body adiposity index; BMI: body mass index; BRI: body roundness index; BSA: body surface area; CI: confidence interval; CMI: cardiometabolic index; CUN-BAE: Clínica Universidad de Navarra-Body Adiposity Estimator; CVAI: Chinese visceral adiposity index; DWRT: Delayed Word Recall Test; HC, hip circumference; LAP: lipid accumulation product; PFM: predicted fat mass; PLM: predicted lean mass; PPF: predicated percent fat; Ref, reference; VAI: visceral adiposity index; WC: waist circumference; WHHR: waist-to-hip-to-height ratio; WHR: waist-to-hip ratio; WHtR: waist-to-height ratio

Note: Adjusted for sex, age, education, occupation, personal income, physical activity, drinking, smoking, self-rated health and baseline DWRT score

^*^P <0.05, ^**^P <0.01, ^***^P <0.001

Table S20. Associations of baseline sarcopenia and sarcopenic obesity with annual change rate of memory function by baseline memory status.

|  | Adjusted mean differences β (95% CI) in annual change rate of DWRT score | | |
| --- | --- | --- | --- |
|  | Baseline DWRT score ≥4 | Baseline DWRT score <4 | P for interaction |
| Sarcopenia |  |  | <0.001 |
| No | Ref. (0) | Ref. (0) |  |
| Yes | -0.52 (-1.04 to -0.001)^*^ | -4.44 (-11.69 to 2.80) |  |
| Sarcopenic obesity (Weight) |  |  | <0.001 |
| Without sarcopenia and obesity | Ref. (0) | Ref. (0) |  |
| Only with sarcopenia | -0.92 (-1.56 to -0.29)^**^ | -4.85 (-13.40 to 3.71) |  |
| Only with obesity | -0.38 (-1.14 to 0.38) | -4.54 (-17.27 to 8.18) |  |
| Sarcopenic obesity | -0.19 (-0.92 to 0.55) | -7.36 (-16.91 to 2.19) |  |
| Sarcopenic obesity (BMI) |  |  | <0.001 |
| Without sarcopenia and obesity | Ref. (0) | Ref. (0) |  |
| Only with sarcopenia | -0.67 (-1.31 to -0.04)^*^ | -0.25 (-9.34 to 8.84) |  |
| Only with obesity | -0.21 (-1.00 to 0.57) | 10.66 (-1.56 to 22.88) |  |
| Sarcopenic obesity | -0.44 (-1.16 to 0.28) | -0.88 (-10.28 to 8.53) |  |
| Sarcopenic obesity (WC) |  |  | <0.001 |
| Without sarcopenia and obesity | Ref. (0) | Ref. (0) |  |
| Only with sarcopenia | -0.60 (-1.20 to 0.003) | -2.40 (-10.65 to 5.84) |  |
| Only with obesity | -1.12 (-1.97 to -0.27)^*^ | 2.32 (-11.37 to 16.01) |  |
| Sarcopenic obesity | -1.30 (-2.07 to -0.53)^***^ | -7.54 (-17.22 to 2.14) |  |
| Sarcopenic obesity (HC) |  |  | <0.001 |
| Without sarcopenia and obesity | Ref. (0) | Ref. (0) |  |
| Only with sarcopenia | -0.78 (-1.40 to -0.17)^*^ | -4.48 (-12.72 to 3.77) |  |
| Only with obesity | -0.38 (-1.17 to 0.42) | 1.31 (-12.07 to 14.69) |  |
| Sarcopenic obesity | -0.32 (-1.07 to 0.43) | -3.17 (-13.01 to 6.67) |  |
| Sarcopenic obesity (WHR) |  |  | <0.001 |
| Without sarcopenia and obesity | Ref. (0) | Ref. (0) |  |
| Only with sarcopenia | -0.63 (-1.51 to 0.25) | -1.09 (-14.13 to 11.94) |  |
| Only with obesity | -0.55 (-1.31 to 0.21) | 1.64 (-10.81 to 14.10) |  |
| Sarcopenic obesity | -1.00 (-1.76 to -0.23)^*^ | -3.99 (-15.56 to 7.58) |  |
| Sarcopenic obesity (WHtR) |  |  | <0.001 |
| Without sarcopenia and obesity | Ref. (0) | Ref. (0) |  |
| Only with sarcopenia | -0.57 (-1.24 to 0.09) | -0.91 (-10.66 to 8.83) |  |
| Only with obesity | -0.94 (-1.69 to -0.18)^*^ | 1.49 (-10.41 to 13.38) |  |
| Sarcopenic obesity | -1.25 (-1.96 to -0.54)^***^ | -6.19 (-15.88 to 3.50) |  |
| Sarcopenic obesity (LAP) |  |  | <0.001 |
| Without sarcopenia and obesity | Ref. (0) | Ref. (0) |  |
| Only with sarcopenia | -0.65 (-1.26 to -0.03)^*^ | -1.73 (-10.33 to 6.86) |  |
| Only with obesity | -1.09 (-1.89 to -0.29)^**^ | 1.81 (-10.91 to 14.53) |  |
| Sarcopenic obesity | -1.25 (-1.99 to -0.50)^**^ | -7.80 (-17.17 to 1.57) |  |
| Sarcopenic obesity (ABSI) |  |  | <0.001 |
| Without sarcopenia and obesity | Ref. (0) | Ref. (0) |  |
| Only with sarcopenia | -0.26 (-0.86 to 0.34) | -2.78 (-11.37 to 5.82) |  |
| Only with obesity | -0.21 (-1.06 to 0.63) | 4.08 (-8.44 to 16.60) |  |
| Sarcopenic obesity | -1.40 (-2.18 to -0.62)^***^ | -3.97 (-13.76 to 5.81) |  |
| Sarcopenic obesity (VAI) |  |  | <0.001 |
| Without sarcopenia and obesity | Ref. (0) | Ref. (0) |  |
| Only with sarcopenia | -0.58 (-1.20 to 0.04) | -1.91 (-10.60 to 6.78) |  |
| Only with obesity | -0.86 (-1.64 to -0.08)^*^ | 2.48 (-9.78 to 14.73) |  |
| Sarcopenic obesity | -1.23 (-1.98 to -0.48)^**^ | -6.94 (-16.51 to 2.63) |  |
| Sarcopenic obesity (CVAI) |  |  | <0.001 |
| Without sarcopenia and obesity | Ref. (0) | Ref. (0) |  |
| Only with sarcopenia | -0.39 (-1.00 to 0.22) | -2.34 (-10.80 to 6.11) |  |
| Only with obesity | -0.45 (-1.31 to 0.40) | -0.27 (-13.50 to 12.96) |  |
| Sarcopenic obesity | -1.20 (-1.96 to -0.43)^**^ | -8.67 (-17.95 to 0.61) |  |
| Sarcopenic obesity (BRI) |  |  | <0.001 |
| Without sarcopenia and obesity | Ref. (0) | Ref. (0) |  |
| Only with sarcopenia | -0.53 (-1.14 to 0.08) | -3.60 (-12.22 to 5.02) |  |
| Only with obesity | -1.08 (-1.93 to -0.24)^*^ | -0.45 (-13.23 to 12.33) |  |
| Sarcopenic obesity | -1.39 (-2.13 to -0.64)^***^ | -6.00 (-15.21 to 3.21) |  |
| Sarcopenic obesity (Conicity index) |  |  | <0.001 |
| Without sarcopenia and obesity | Ref. (0) | Ref. (0) |  |
| Only with sarcopenia | -0.13 (-0.73 to 0.47) | -5.73 (-14.57 to 3.11) |  |
| Only with obesity | 0.76 (-0.10 to 1.61) | -1.94 (-14.07 to 10.19) |  |
| Sarcopenic obesity | -0.87 (-1.66 to -0.08)^*^ | -3.84 (-14.14 to 6.46) |  |
| Sarcopenic obesity (BAI) |  |  | <0.001 |
| Without sarcopenia and obesity | Ref. (0) | Ref. (0) |  |
| Only with sarcopenia | -0.53 (-1.15 to 0.08) | -1.66 (-10.46 to 7.14) |  |
| Only with obesity | -0.41 (-1.23 to 0.41) | 8.73 (-3.82 to 21.28) |  |
| Sarcopenic obesity | -0.83 (-1.56 to -0.09)^*^ | -0.93 (-10.65 to 8.79) |  |
| Sarcopenic obesity (CMI) |  |  | <0.001 |
| Without sarcopenia and obesity | Ref. (0) | Ref. (0) |  |
| Only with sarcopenia | -0.55 (-1.17 to 0.07) | -2.23 (-10.83 to 6.37) |  |
| Only with obesity | -0.93 (-1.73 to -0.14)^*^ | 1.11 (-11.27 to 13.50) |  |
| Sarcopenic obesity | -1.31 (-2.06 to -0.57)^***^ | -7.90 (-17.46 to 1.66) |  |
| Sarcopenic obesity (BSA) |  |  | <0.001 |
| Without sarcopenia and obesity | Ref. (0) | Ref. (0) |  |
| Only with sarcopenia | -0.85 (-1.49 to -0.21)^**^ | -5.68 (-14.14 to 2.78) |  |
| Only with obesity | -0.10 (-0.85 to 0.65) | -4.81 (-17.57 to 7.95) |  |
| Sarcopenic obesity | -0.03 (-0.78 to 0.72) | -5.94 (-15.60 to 3.71) |  |
| Sarcopenic obesity (WHHR) |  |  | <0.001 |
| Without sarcopenia and obesity | Ref. (0) | Ref. (0) |  |
| Only with sarcopenia | -0.40 (-1.00 to 0.20) | -0.51 (-9.23 to 8.20) |  |
| Only with obesity | -0.88 (-1.76 to 0.01) | -1.52 (-13.84 to 10.80) |  |
| Sarcopenic obesity | -1.46 (-2.22 to -0.71)^***^ | -11.24 (-20.58 to -1.90)^*^ |  |
| Sarcopenic obesity (PFM) |  |  | <0.001 |
| Without sarcopenia and obesity | Ref. (0) | Ref. (0) |  |
| Only with sarcopenia | -0.73 (-1.36 to -0.10)^*^ | -7.32 (-16.08 to 1.45) |  |
| Only with obesity | -0.40 (-1.18 to 0.38) | -10.68 (-23.38 to 2.02) |  |
| Sarcopenic obesity | -0.51 (-1.24 to 0.22) | -8.59 (-17.98 to 0.80) |  |
| Sarcopenic obesity (PLM) |  |  | <0.001 |
| Without sarcopenia and obesity | Ref. (0) | Ref. (0) |  |
| Only with sarcopenia | -0.88 (-1.52 to -0.24)^**^ | -7.84 (-16.33 to 0.66) |  |
| Only with obesity | -0.26 (-1.01 to 0.49) | -12.28 (-24.69 to 0.13) |  |
| Sarcopenic obesity | -0.16 (-0.91 to 0.58) | -8.67 (-18.49 to 1.16) |  |
| Sarcopenic obesity (PPF) |  |  | <0.001 |
| Without sarcopenia and obesity | Ref. (0) | Ref. (0) |  |
| Only with sarcopenia | -0.69 (-1.30 to -0.08)^*^ | -4.02 (-12.68 to 4.65) |  |
| Only with obesity | -0.88 (-1.72 to -0.04)^*^ | 0.29 (-13.12 to 13.70) |  |
| Sarcopenic obesity | -0.89 (-1.63 to -0.14)^*^ | -4.85 (-14.00 to 4.31) |  |
| Sarcopenic obesity (CUN-BAE) |  |  | <0.001 |
| Without sarcopenia and obesity | Ref. (0) | Ref. (0) |  |
| Only with sarcopenia | -0.72 (-1.33 to -0.10)^*^ | -3.24 (-12.07 to 5.59) |  |
| Only with obesity | -0.87 (-1.70 to -0.04)^*^ | 3.33 (-10.10 to 16.76) |  |
| Sarcopenic obesity | -0.84 (-1.57 to -0.10)^*^ | -3.66 (-13.07 to 5.75) |  |

ABSI: a body shape index; BAI: body adiposity index; BMI: body mass index; BRI: body roundness index; BSA: body surface area; CI: confidence interval; CMI: cardiometabolic index; CUN-BAE: Clínica Universidad de Navarra-Body Adiposity Estimator; CVAI: Chinese visceral adiposity index; DWRT: Delayed Word Recall Test; HC, hip circumference; LAP: lipid accumulation product; PFM: predicted fat mass; PLM: predicted lean mass; PPF: predicated percent fat; Ref, reference; VAI: visceral adiposity index; WC: waist circumference; WHHR: waist-to-hip-to-height ratio; WHR: waist-to-hip ratio; WHtR: waist-to-height ratio

Note: Adjusted for sex, age, education, occupation, personal income, physical activity, drinking, smoking, self-rated health and baseline DWRT score

^*^P <0.05, ^**^P <0.01, ^***^P <0.001


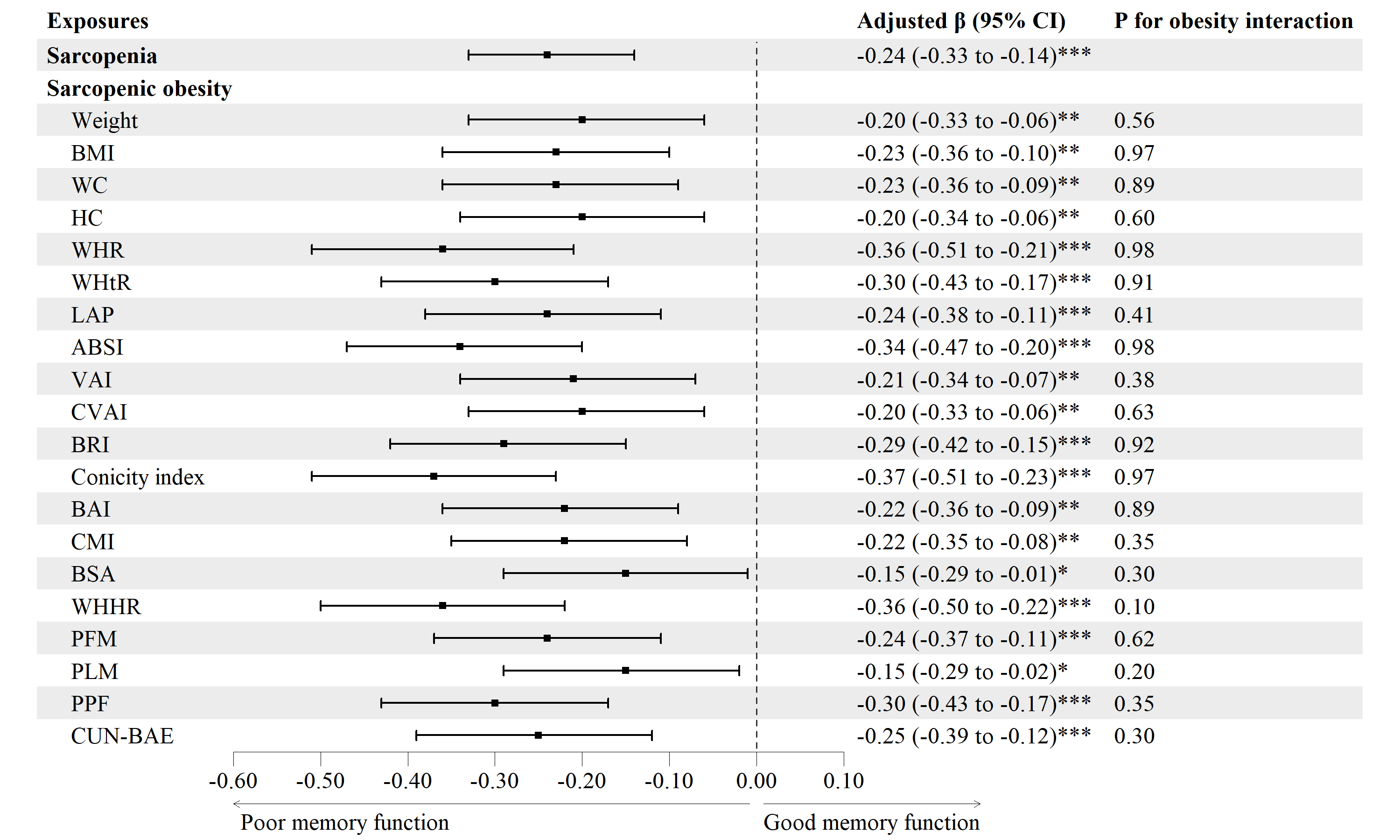


Figure S1. Associations of sarcopenia and sarcopenic obesity with memory function at baseline.

Note: ABSI: a body shape index; BAI: body adiposity index; BMI: body mass index; BRI: body roundness index; BSA: body surface area; CMI: cardiometabolic index; CUN-BAE: Clínica Universidad de Navarra-Body Adiposity Estimator; CVAI: Chinese visceral adiposity index; DWRT: Delayed Word Recall Test; HC, hip circumference; LAP: lipid accumulation product; PFM: predicted fat mass; PLM: predicted lean mass; PPF: predicated percent fat; Ref, reference; VAI: visceral adiposity index; WC: waist circumference; WHHR: waist-to-hip-to-height ratio; WHR: waist-to-hip ratio; WHtR: waist-to-height ratio

Adjusted β (95% CI): Adjusted for sex, age, education, occupation, personal income, physical activity, drinking, smoking and self-rated health

*P <0.05, **P <0.01, ***P <0.001


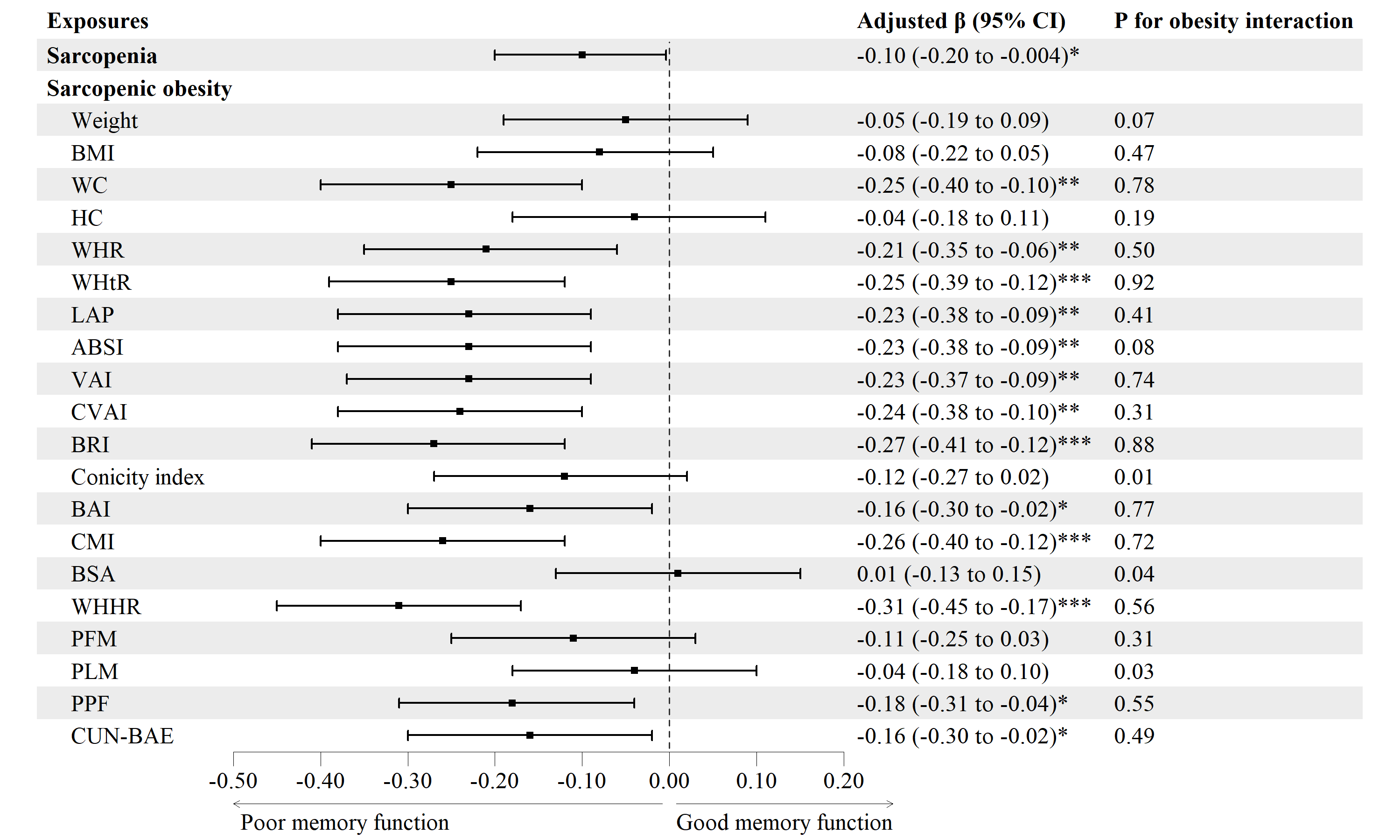


Figure S2. Associations of sarcopenia and sarcopenic obesity with memory function at follow-up.

Note: ABSI: a body shape index; BAI: body adiposity index; BMI: body mass index; BRI: body roundness index; BSA: body surface area; CMI: cardiometabolic index; CUN-BAE: Clínica Universidad de Navarra-Body Adiposity Estimator; CVAI: Chinese visceral adiposity index; DWRT: Delayed Word Recall Test; HC, hip circumference; LAP: lipid accumulation product; PFM: predicted fat mass; PLM: predicted lean mass; PPF: predicated percent fat; Ref, reference; VAI: visceral adiposity index; WC: waist circumference; WHHR: waist-to-hip-to-height ratio; WHR: waist-to-hip ratio; WHtR: waist-to-height ratio

Adjusted β (95% CI): Adjusted for sex, age, education, occupation, personal income, physical activity, drinking, smoking, self-rated health and baseline DWRT score

*P <0.05, **P <0.01, ***P <0.001


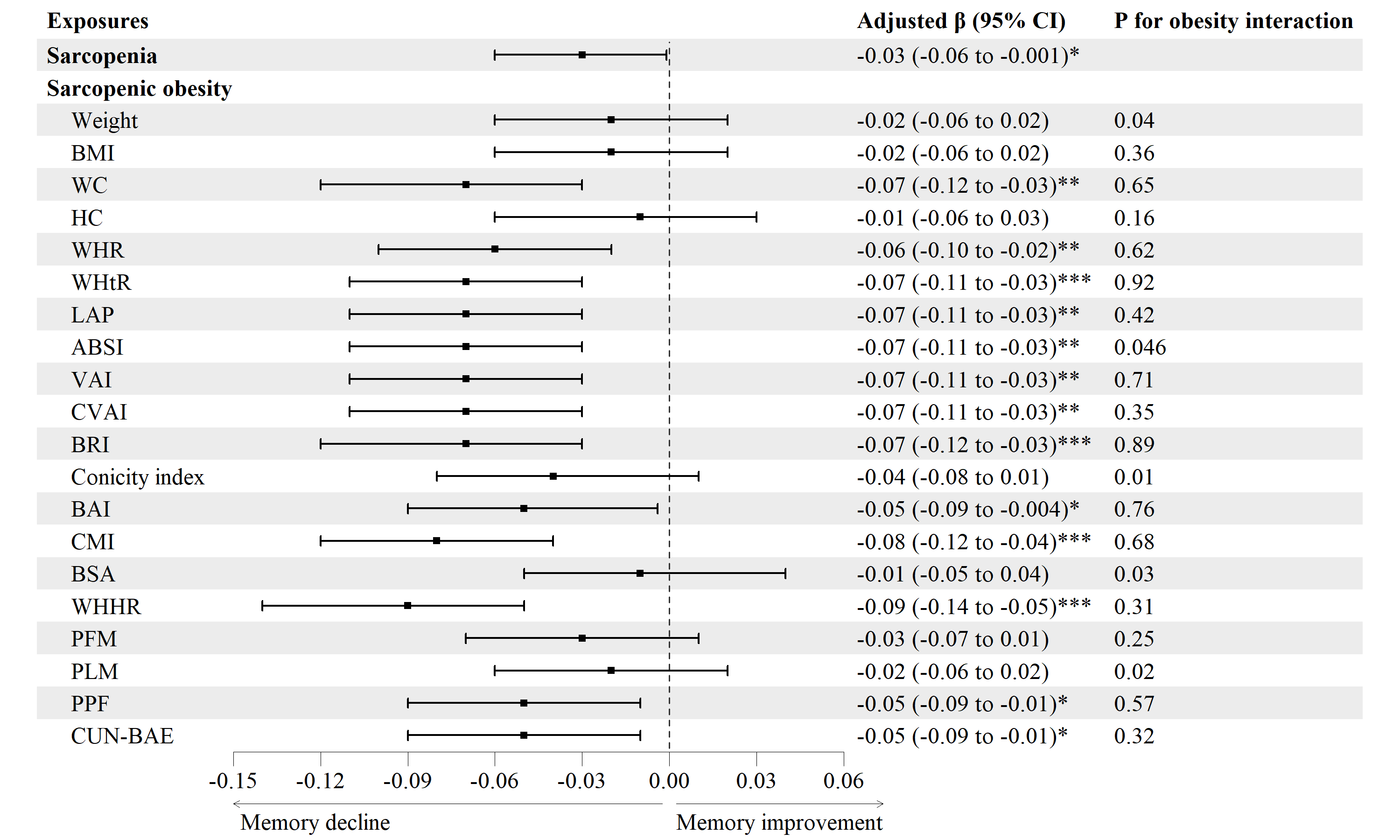


Figure S3. Associations of sarcopenia and sarcopenic obesity with mean annual change of memory function.

Note: ABSI: a body shape index; BAI: body adiposity index; BMI: body mass index; BRI: body roundness index; BSA: body surface area; CMI: cardiometabolic index; CUN-BAE: Clínica Universidad de Navarra-Body Adiposity Estimator; CVAI: Chinese visceral adiposity index; DWRT: Delayed Word Recall Test; HC, hip circumference; LAP: lipid accumulation product; PFM: predicted fat mass; PLM: predicted lean mass; PPF: predicated percent fat; Ref, reference; VAI: visceral adiposity index; WC: waist circumference; WHHR: waist-to-hip-to-height ratio; WHR: waist-to-hip ratio; WHtR: waist-to-height ratio

Adjusted β (95% CI): Adjusted for sex, age, education, occupation, personal income, physical activity, drinking, smoking, self-rated health and baseline DWRT score

*P <0.05, **P <0.01, ***P <0.001


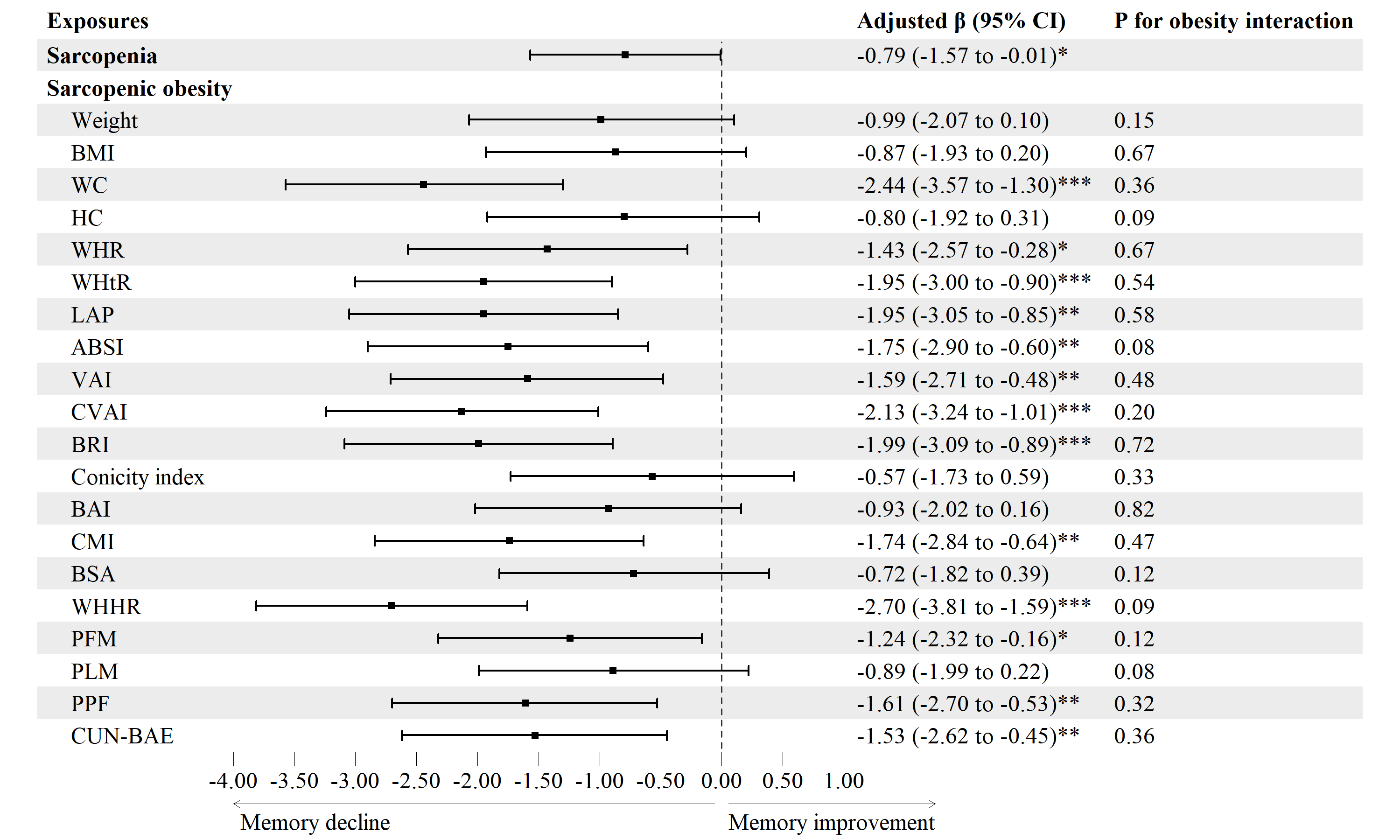


Figure S4. Associations of sarcopenia and sarcopenic obesity with mean annual change rate of memory function.

Note: ABSI: a body shape index; BAI: body adiposity index; BMI: body mass index; BRI: body roundness index; BSA: body surface area; CMI: cardiometabolic index; CUN-BAE: Clínica Universidad de Navarra-Body Adiposity Estimator; CVAI: Chinese visceral adiposity index; DWRT: Delayed Word Recall Test; HC, hip circumference; LAP: lipid accumulation product; PFM: predicted fat mass; PLM: predicted lean mass; PPF: predicated percent fat; Ref, reference; VAI: visceral adiposity index; WC: waist circumference; WHHR: waist-to-hip-to-height ratio; WHR: waist-to-hip ratio; WHtR: waist-to-height ratio

Adjusted β (95% CI): Adjusted for sex, age, education, occupation, personal income, physical activity, drinking, smoking, self-rated health and baseline DWRT score

*P <0.05, **P <0.01, ***P <0.001

Figure S5. Flowchart showing selection of SNPs related to sarcopenia-related traits used as instruments in analysis of effects on working memory: (A) Hand grip strength (left) (B) Hand grip strength (right) (C) Muscle weakness (D) Walking pace (E) Appendicular lean mass (F) Whole-body lean mass

Figure S6. Flowchart showing selection of SNPs related to sarcopenia-related traits used as instruments in analysis of effects on memory loss: (A) Hand grip strength (left) (B) Hand grip strength (right) (C) Muscle weakness (D) Walking pace (E) Appendicular lean mass (F) Whole-body lean mass
